# Jusanin, a New Flavonoid from *Artemisia commutata* with an In Silico Inhibitory Potential against the SARS-CoV-2 Main Protease

**DOI:** 10.3390/molecules27051636

**Published:** 2022-03-01

**Authors:** Yerlan M. Suleimen, Rani A. Jose, Raigul N. Suleimen, Christoph Arenz, Margarita Y. Ishmuratova, Suzanne Toppet, Wim Dehaen, Bshra A. Alsfouk, Eslam B. Elkaeed, Ibrahim H. Eissa, Ahmed M. Metwaly

**Affiliations:** 1 The International Centre for Interdisciplinary Solutions on Antibiotics and Secondary Metabolites, Republican Collection of Microorganisms, Nur-Sultan 010000, Kazakhstan; syerlan75@yandex.kz; 2 The Laboratory of Engineering Profile of NMR Spectroscopy, Sh. Ualikhanov Kokshetau University, Kokshetau 020000, Kazakhstan; 3 Molecular Design & Synthesis, Department of Chemistry, Catholic University of Leuven, B-3001 Leuven, Belgium; alphmanie@gmail.com (R.A.J.); tsuzanne@kuleuven.be (S.T.); wim.dehaen@kuleuven.be (W.D.); 4 Department of Chemistry, St. Dominic’s College, Mahatma Gandhi University, Kanjirappally 686512, India; 5 Department of Technical Physics, Faculty of Physics and Technology, L.N. Gumilyov Eurasian National University, Nur-Sultan 010010, Kazakhstan; 6 Institut für Chemie der Humboldt-Universität zu, D-12489 Berlin, Germany; arenzchr@hu-berlin.de; 7 Department of Botany, E.A. Buketov Karaganda University, Karaganda 100024, Kazakhstan; margarita.ishmur@mail.ru; 8 Department of Pharmaceutical Sciences, College of Pharmacy, Princess Nourah bint Abdulrahman University, P.O. Box 84428, Riyadh 11671, Saudi Arabia; baalsfouk@pnu.edu.sa; 9 Department of Pharmaceutical Sciences, College of Pharmacy, AlMaarefa University, Riyadh 13713, Saudi Arabia; ikaeed@mcst.edu.sa; 10 Pharmaceutical Medicinal Chemistry & Drug Design Department, Faculty of Pharmacy (Boys), Al-Azhar University, Cairo 11884, Egypt; ibrahimeissa@azhar.edu.eg; 11 Pharmacognosy and Medicinal Plants Department, Faculty of Pharmacy (Boys), Al-Azhar University, Cairo 11884, Egypt; 12 Biopharmaceutical Products Research Department, Genetic Engineering and Biotechnology Research Institute, City of Scientific Research and Technological Applications, Alexandria 21934, Egypt

**Keywords:** *Artemisia commutata*, new flavonoid, Jusanin, COVID-19 main protease, molecular similarity, DFT, molecular docking, ADMET, toxicity, molecular dynamic simulations

## Abstract

A new flavonoid, Jusanin, (**1**) has been isolated from the aerial parts of *Artemisia commutata*. The chemical structure of Jusanin has been elucidated using 1D, 2D NMR, and HR-Ms spectroscopic methods to be 5,2′,4′-trihydroxy-6,7,5′-trimethoxyflavone. Being new in nature, the inhibition potential of **1** has been estimated against SARS-CoV-2 using different in silico techniques. Firstly, molecular similarity and fingerprint studies have been conducted for Jusanin against co-crystallized ligands of eight different SARS-CoV-2 essential proteins. The studies indicated the similarity between **1** and **X77**, the co-crystallized ligand SARS-CoV-2 main protease (PDB ID: 6W63). To confirm the obtained results, a DFT study was carried out and indicated the similarity of (total energy, HOMO, LUMO, gap energy, and dipole moment) between **1** and **X77**. Accordingly, molecular docking studies of **1** against the target enzyme have been achieved and showed that **1** bonded correctly in the protein’s active site with a binding energy of −19.54 Kcal/mol. Additionally, in silico ADMET in addition to the toxicity evaluation of Jusanin against seven models have been preceded and indicated the general safety and the likeness of Jusanin to be a drug. Finally, molecular dynamics simulation studies were applied to investigate the dynamic behavior of the M^pro^-Jusanin complex and confirmed the correct binding at 100 ns. In addition to 1, three other metabolites have been isolated and identified to be сapillartemisin A (**2**), methyl-3-[S-hydroxyprenyl]-cumarate (**3**), and β-sitosterol (**4**).

## 1. Introduction

Natural products served for thousands of years as the major source for the basic needs of treatment and food in human life [1,2]. Scientists investigated plants [3,4] and microbes [5,6] to deeply understand the causatives of these powers. Various metabolites have been isolated and exhibited promising activities as alkaloids [7], flavonoids [8,9], isochromenes [10], α-pyrones [11], diterpenes [12], sesquiterpenes [13,14,15], steroids [16], and saponins [17,18]. 

*Artemisia commutata* Besser is a perennial herb found in northern and eastern Kazakhstan. The plant is also found in Europe, Mongolia, Altai, western and eastern Siberia, Primorsky Krai, Amur Region, and Sakhalin in the Far East, as well as in the Volga-Kama region of the European part of Russia [19].

The genus *Artemisia* was and still is an interesting target for the scientist to study. The isolation and identification of sesquiterpene lactones have been recorded severally in a lot of *Artemisia* species such as *А. tschernieviana* and *A. sublessingiana* [20]. Furthermore, the presence of flavonoids was also reported in *A. albida* [21] and *A. santolinifolia* [22]. The isolation of promising secondary metabolites was reported before as epi-аshantin from *A. sieversiana* [23], anhydroaustricin from *A. albida* [24], matricarin from *A. austriaca* [25], cirsineol and cubreva lactone in *A. umbrosa* [26]. Recently, the in silico anti-SARS-CoV-2 papain-like protease activities of two derivatives of 2-Phenoxychromone of *Artemisia* spp. has been reported [27]. Additionally, chemical and biological properties of essential oils of diverse *Artemisia* species were discussed in various records as *A. kasakorum* [28] *A. lercheana*, *A. sieversiana* [29], *A.* species [30], *A. umbrosa* [31], five *Artemisia* species [32], *A. gurganica* [33], *A. proceriformis* [34], *A. terrae-albae* [35], *A. keiskeana* [36], *A. littoricola*, *A. mandshurica* [37], and *A. commutata* [38].

The WHO stated on the COVID-19 dashboard on 27 December 2021, that the global confirmed infections had reached 279,114,972 and the number of deaths passed 5,397,580 [39]. These alarming numbers require massive and continuous work in the field of drug discovery to face the COVID-19 pandemic.

The computer-aided (in silico, computational or cheminformatics) drug design and discovery is a widely employed approach. The ability to determine both the physical and chemical properties of a molecule enabled the identification of molecular similarities and hence the prediction of activity. Additionally, molecular docking and dynamic simulations studies can determine the interaction of a specific molecule with a certain protein successfully [40]. Our team presented several reports in which they utilized computational chemistry to find a cure against COVID-19 [41,42,43,44,45].

Interestingly, the inhibitory effect of flavonoids has been reported before. For instance, rutin was predicted to active against COVID-19 3CL proteinase through exhibiting the best binding affinity among 2030 other natural compounds [46].

We herein report the isolation and structure elucidation of the new flavonoid, Jusanin, (jusan is the original kazak name of wormwood) from the aerial parts of *A. commutata*. Because Jusanin is a new compound, its potential anti-COVID-19 effect was investigated. Additionally, ADMET, toxicity, and DFT properties of Jusanin have been studied. Finally, molecular dynamics simulation studies confirmed the expected affinity of Jusanin. Besides Jusanin, three known metabolites have been isolated and identified as сapillartemisin A (**2**), methyl-3-[S-hydroxyprenyl]-cumarate (**3**), and β-sitosterol (**4**).

## 2. Results and Discussion

### 2.1. Isolation of Jusanin

To study the composition of *A. commutata* [47], 0.94 kg of the aboveground parts were collected in the East Kazakhstan region (Western Altai Mountains). Raw materials afforded 20 g of chloroform extract.

**Figure 1:**
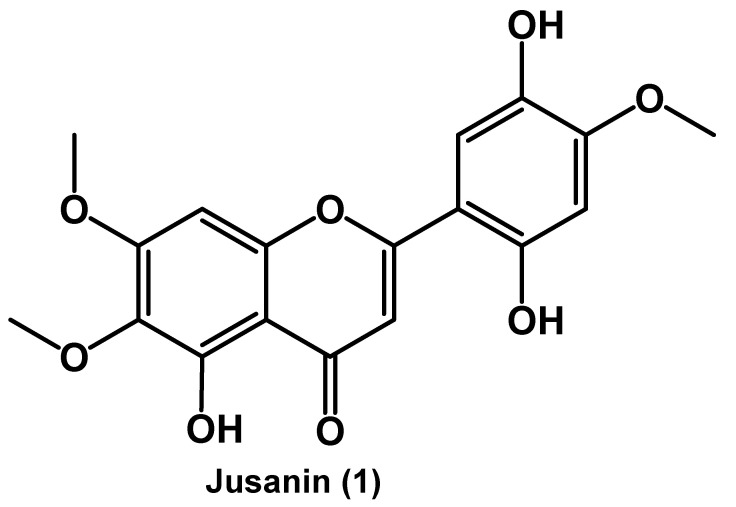
The chemical structure of Jusanin (**1**).

The chloroform extract was subjected to a further isolation by silica gel column using heptane-ethyl acetate-methanol as a mobile phase in a manner of increasing polarity. Fraction 5 was further purified utilizing Sephadex LH-20 to afford the crystalline substance (**1**) a yellow color with a m.p. more than 350 °C. Based on the spectral data of UV, ^1^H, and ^13^C NMR, with two-dimensional experiments, HSQC, Dept, COSY, and HMBC (Table 1), compound **1** was identified to be the new compound 5,2′,4′-trihydroxy-6,7,5′-trimethoxyflavone, Jusanin (Figure 1). The molecular formula C_18_H_16_O_8_ was decided by the HR-ESI-MS experiment, (+ve mode), that displayed a pseudo molecular ion peak [M + H]^+^ at *m*/*z* 361.0934 (calcd. for C_18_H_17_O_8_, 361.0923).

**Figure 2:**
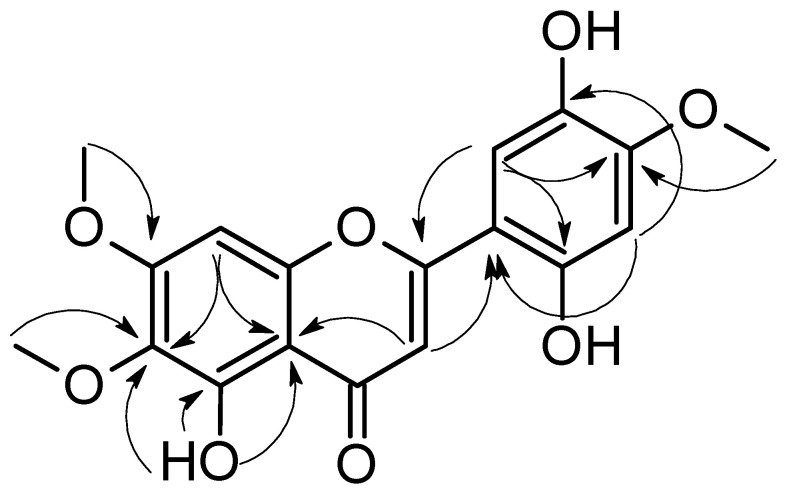
Key HMBC correlations in Jusanin.

Compound **1** was isolated as a yellow powder with a m.p. more than 350 °C. The ^1^H NMR spectrum of **1** showed four singlet signals of aromatic protons at δH 7.11 (s, H-3), δH 6.97 (s, H-8), δH 6.58 (s, H-3′), and δH 7.45 (s, H-6′). Furthermore, three signals of methoxy groups were detected at δH 3.74 (s), δH 3.82 (s), and δH 3.94 (s) ppm. The characteristic chelated signal of the OH at C-5 appeared as a sharp singlet at δH 13.06 (due to intramolecular hydrogen bonding) (see Table 1). The ^13^С, HSQC, and HMBC spectra of **1** indicated the presence of 15 carbon atoms in addition to 3 methoxy groups. The chemical structure of **1** was further confirmed by the heteronuclear multiple bond correlation (HMBC) experiment (Figure 2). Two HMBC correlations were detected between the proton at δH 7.11 (H, s, H-3) and C-10 (δC 105.3) and C-1′ (δC 107.2). Moreover, the OH at C-5 was engaged in three HMBC correlations with C-5 (δC 152.6), C-6 (δC 131.7), and C-10 (δC 105.3). The ring B was confirmed through the HMBC correlations between the proton at δH 6.58 (H, s, H-3′) and C-1′ (δC 107.2) as well as C-5′ (δC 139.7) in addition to the correlations between the proton at δH 7.45 (H, s, H-6′) C-2′ (δC 152.4), and C-4′ (δC 142.0). The HMBC spectrum indicated the attachment of the methoxy groups at δH 3.74 (s), δH 3.82 (s), and δH 3.94 (s) ppm to the carbon atoms C-6 (δC 60.0), C-7 (δC 56.7), and C-4′ (δC 56.5), respectively.

**Figure 3:**
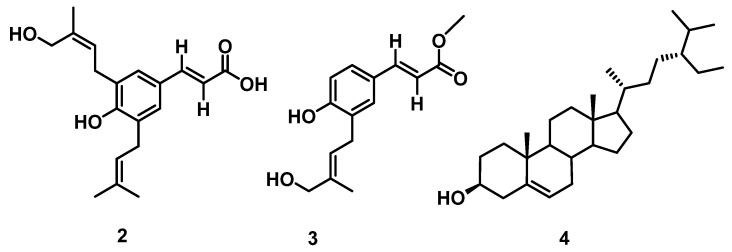
Chemical structures of compounds **2**–**4**.

Further chromatographic techniques led to the isolation of **2** (200 mg), **3** (20 mg), and **4** (50 mg). The spectral data of **2**–**4** were found to correspond to capillartemisin A [48], methyl-3-[S-hydroxyprenyl]-cumarate [49], and β-sitosterol [50], respectively (Figure 3).

### 2.2. Molecular Similarity Study

The basic principle to understand the molecular similarity technique is the well-known link between the bioactivity of a ligand and interactions of that ligand with a protein target. These interactions are based on physical and chemical properties as the ability to donate or accept hydrogen bond interactions as well as the hydrophobic interactions. In consequence, if two molecules have similar chemical structures, they are expected to have similar steric configuration in addition to similar atoms that act as H-bond donors, acceptors, or hydrophobic centers. Accordingly, these two compounds are predicted to exhibit similar bioactivity too [51].

**Figure 4:**
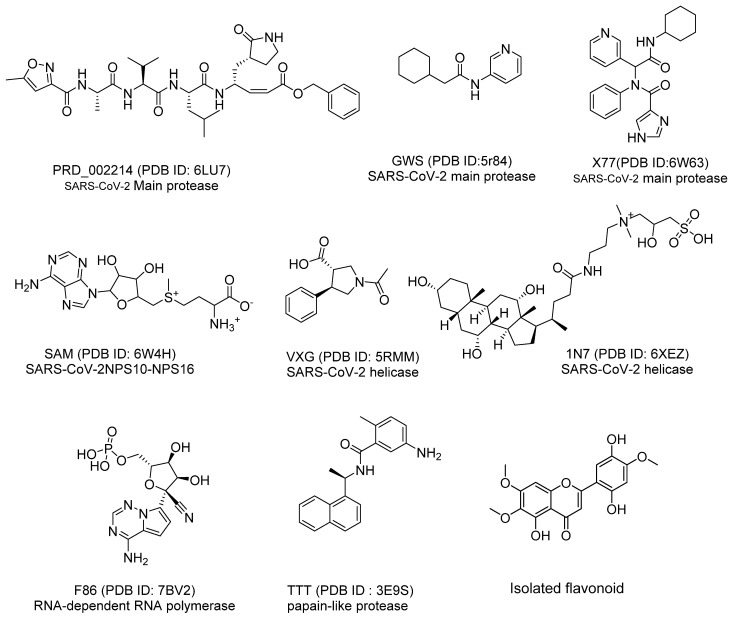
The chemical structures of co-crystallized ligands of SARS-Cov-2 proteins and Jusanin (the isolated flavonoid).

The chemical structure of Jusanin was compared to the chemical structures of eight co-crystallized ligands of eight essential SARS-CoV-2 proteins (Figure 4). This experiment was designed to check if Jusanin has a similarity with one of these ligands and hence it may show a potential inhibitory impact anti-COVID-19 effect.

Discovery studio software was utilized to explore the following molecular properties in Jusanin and the nine ligands; partition coefficient (ALog p) [52], molecular weight (M. Wt) [53], hydrogen bond (H-bond) donors (HBA) [54], H-bond acceptors (HBD) [55], number of rotatable bonds [56], number of rings, and also aromatic rings [57], as well as molecular fractional polar surface area (MFPSA) [58].

**Figure 5:**
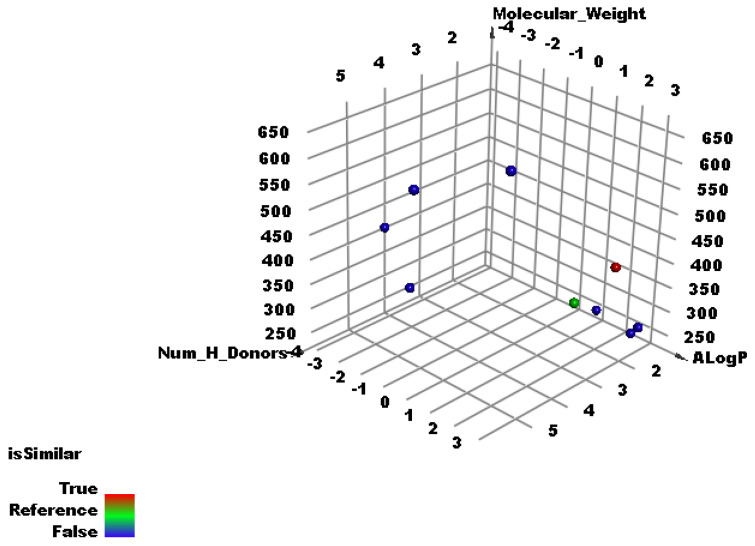
The results of the similarity analysis of Jusanin.

As shown in Figure 5 which clarifies the correlation between Alog p, molecular weight, and number of hydrogen bond donors, the co-crystalized ligand (**X77**) of the SARS-Cov-2 main protease (PDB ID: 6W63) (red ball) has good similarity with Jusanin (green ball). The minimum distance between the red and green ball is 0.92. and on the other hand, the co-crystallized ligands of the other SARSCoV-2 proteins (blue balls) did not show similarity with Jusanin (green ball).

The results revealed a high degree of similarity between the Jusanin and **X77** (Table 2 and Figure 5).

### 2.3. Structural Fingerprints Study

In order to verify the obtained molecular similarity results, a fingerprint study was carried out using Discovery Studio. The fingerprint technique calculates the 2D molecular features of two different compounds or more in a binary format. The fingerprint study examined the presence or absence of the coming parameters: charge [59], hybridization [60], H-bond acceptor and donor [61], positive and negative ionizable [62], halogen [63], aromatic [64], and ALogP [65].

In structural keys, the chemical structure of a molecule is encoded into a binary bit string (0′s and 1′s), each bit of which corresponds to a “pre-defined” structural feature (e.g., substructure or fragment). If the molecule has a pre-defined feature, the bit position corresponding to this feature is set to 1 (ON). Otherwise, it is set to 0 (OFF) [66].

Fingerprint approach depends on the Tanimoto coefficient. In this work, there are three parameters controlling the binary bit string. SA refers to the bits number that computed in the co-crystallized ligands of SARSCoV-2 proteins and Jusanin. SB refers to the bits number that computed in the co-crystallized ligands of SARSCoV-2 proteins but not in Jusanin. SC refers to the bits number that computed in Jusanin but not in the co-crystallized ligands of SARSCoV-2 proteins.

The results of the fingerprint study revealed that **X77** has the highest similarity value (0.512) with Jusanin. In addition, **X77** showed high SA value with acceptable rage of SB and SC values indicating the presence of a high number of bits in both **X77** and Jusanin. These findings verified the significant fingerprint similarity of Jusanin with **X77** (Table 3).

### 2.4. Flexible Alignment Studies

**Figure 6:**
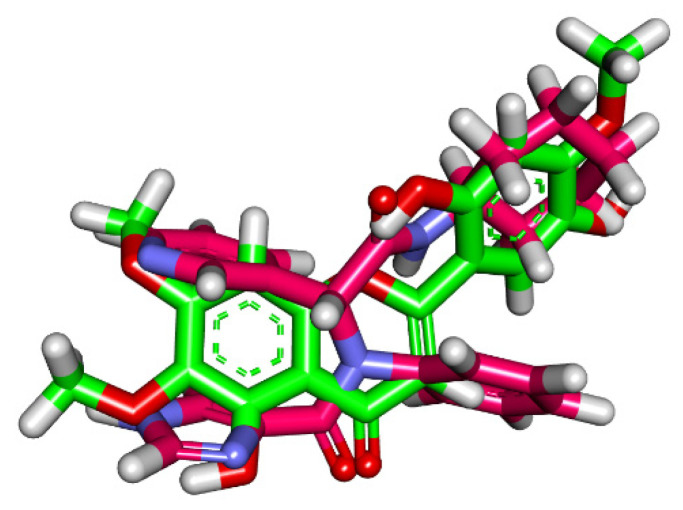
Flexible alignment of Jusanin (green) with **X77** (pink).

Three-dimensional flexible alignment of Jusanin with the co-crystallized ligands (**X77**) was studied. The result of flexible alignment revealed the generally good overlap of Jusanin with the reference molecule (**X77**). In addition, Jusanin showed the same spatial orientation of **X77**. In detail, the 2,3-dimethoxyphenol moiety showed the same orientation of both pyridine and 1H-imidazole moieties of **X77**. In addition, the 4H-pyran-4-one moiety of Jusanin exhibited close orientation to the phenyl ring of **X77**. Furthermore, the 2-methoxybenzene-1,4-diol moiety of Jusanin showed the same orientation of the cyclohexyl moiety of **X77** (Figure 6).

### 2.5. DFT Studies

**Figure 7:**
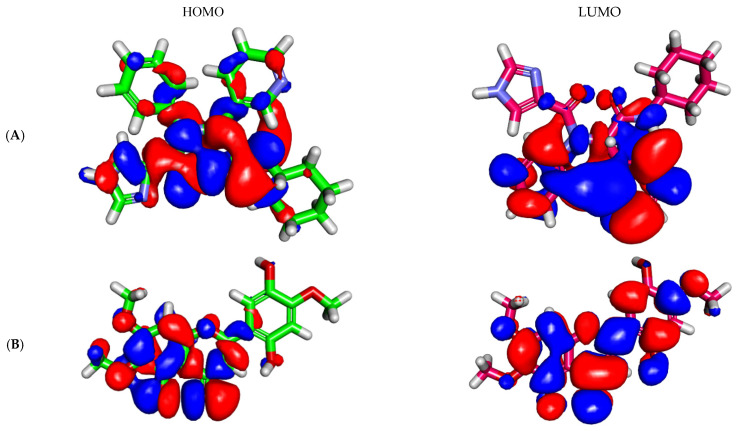
MO spatial distribution of (**A**) Jusanin and (**B**) **X77**.

**Figure 8:**
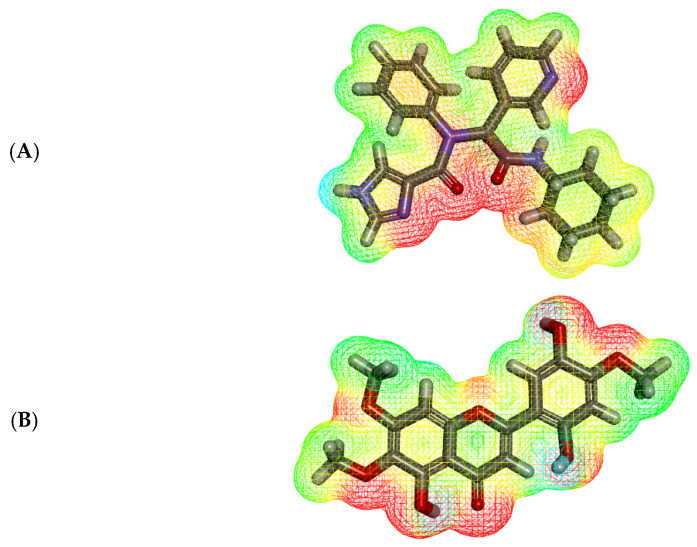
MEP map of (**A**) **X77** and (**B**) Jusanin.

The DFT study is an in silico method that calculates the molecular orbital analysis in addition to the molecular electrostatic potential maps of a certain compound based on the examined parameters (Table 4) [67,68]. The DFT study determines the degree of reactivity of a compound. Accordingly, the DFT properties of Jusanin and **X77** were studied by Discovery Studio software to disclose the similarity between them from this point. The results were summarized in Table 4, Figure 7 and Figure 8. The functional used in this test was PWC of local density approximation (LDA). In addition, the quality was adjusted to be Coarse which uses DN basis set with SCF density convergence of 1.0e-4 as utilized from Accelrys in the DMol3 module of Materials Studio package.

#### 2.5.1. Molecular Orbital (MO) Analysis

The compounds also exhibited almost equal dipole moment values of 2.395 and 2.906. Additionally, the gap energy of Jusanin (0.083 Ha) was less than that of **X77** (0.096 Ha) which denotes a higher degree of reactivity. Consequently, Jusanin may serve as a promising candidate for further studies.

As shown in Figure 7, the analysis of EHOMO and ELUMO, which are corresponding to the highest occupied and lowest unoccupied molecular orbital energies, respectively. EHOMO and ELUMO were related to the chemical reactivity of the investigated compound and its stability. The HOMO spatial (the electron transfer zones) distributions of **X77** are mainly on the 1*H*-imidazole and the two amide moieties, while its LUMO spatial (the electron acceptor zones) distributions are on the pyridine and phenyl moieties. For compound Jusanin, the HOMO spatial distributions are mainly distributed on 5-hydroxy-6,7-dimethoxy-4*H*-chromen-4-one moiety, while its LUMO spatial distributions are located on the 2-methoxybenzene-1,4-diol.

#### 2.5.2. Molecular Electrostatic Potential Maps (MEP)

MEP is an in silico method that calculates partial charges [69], electronegativity [70], intermolecular interaction [71], and chemical reactivity [71] to determine the electrostatic potential of a compound in a 3D form [72]. In MEP study, the electronegative atoms (H-bond acceptors) appear in red. On the other side, the electron-poor atoms (H-bond donors) appear in blue. Additionally, the neutral atoms (hydrophobic interactors) appear in a green to yellow color [73].

The MEP of Jusanin and **X77** was illustrated in Figure 8A,B, respectively. The ligand, **X77**, showed four red patches indicating H-bond acceptors and one blue patch (an H-bond donor). Additionally, the high possibility for hydrophobic interactions was indicated by the presence of yellow patches on the aromatic and aliphatic moieties. 

For Jusanin, it showed eight red patches (H-bond acceptors) and two blue patches (H-bond donors). Additionally, there is a yellow patch on the aromatic system indicating the possibility of hydrophobic interaction. These findings validate the ability of Jusanin to interact with M^pro^ like **X77**.

### 2.6. Docking Studies

**Figure 9:**
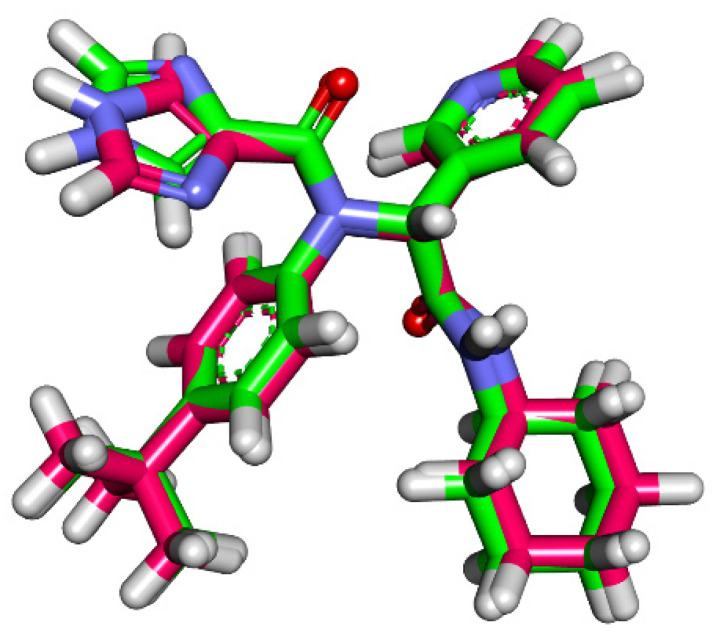
Superimposition of the docking pose (dark green) and the co-crystallized (pink) of the same molecule.

To validate the results of the previous experiments, the binding interaction of Jusanin was investigated against the SARS-CoV-2 main protease (PDB ID: 6W63) through docking studies. Compound **X77**, was used as a reference. The binding free energy (∆G) and the correct binding mode were employed as the base to evaluate the biding. First of all, a validation procedure of the docking process was applied via re-docking of **X77** against M^pro^. The resulting RMSD value was 1.7 °A to indicate the validity of the docking process (Figure 9).

**Figure 10:**
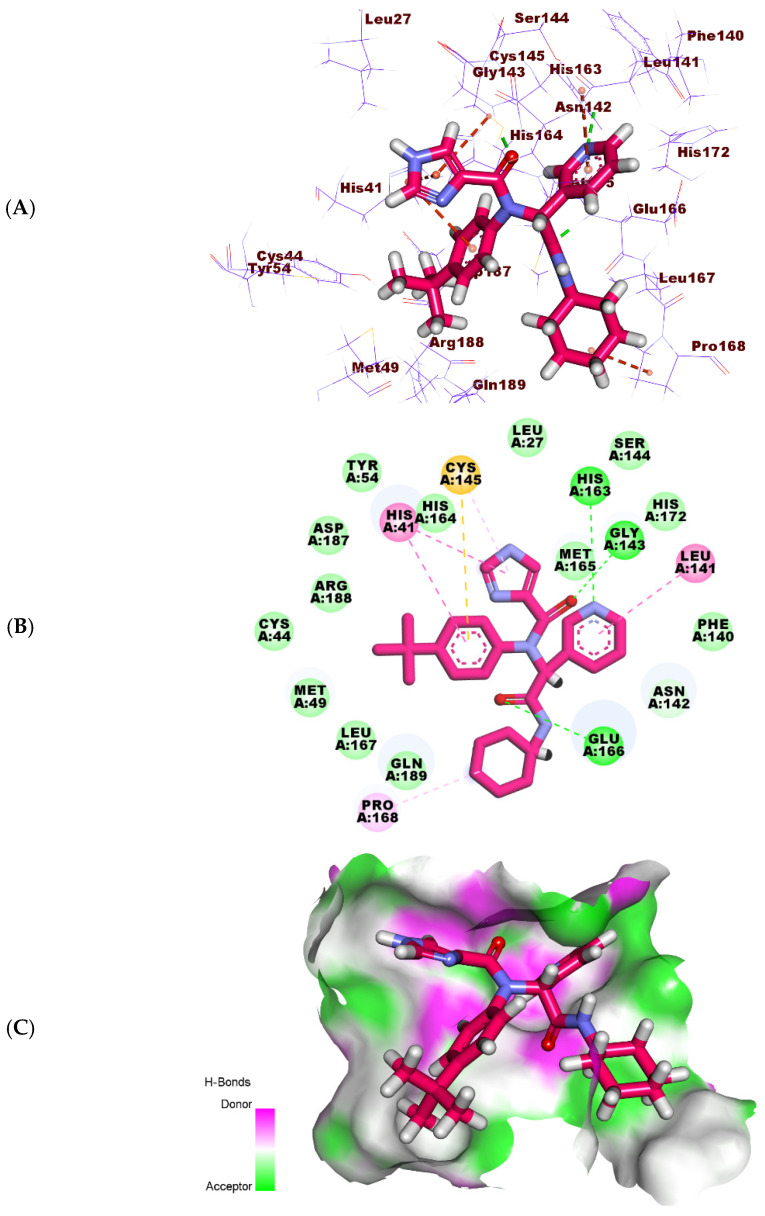
(**A**) Three-dimensional, (**B**) two-dimensional, and (**C**) surface mapping of **X77** docked into M^pro^.

The binding of **X77** exhibited a binding free energy of −22.81 kcal/mol. The pyridine moiety was buried in the first pocket of the receptor to form one H-bond with His163 and one hydrophobic interaction with Leu141. Furthermore, the 1H-imidazole was oriented into the second pocket and interacted hydrophobically with Cys145 and His41. The tert-butylbenzene moiety occupied the third pocket closely to the amino acids Arg188, Met49, and Leu167. The same moiety reacted with two hydrophobic interactions with Cys145 and His41. The cyclohexyl moiety was buried in the fourth pocket forming a hydrophobic interaction with the amino acid Pro168. The amide moiety formed one H-bond interaction with Glu166 (Figure 10).

**Figure 11:**
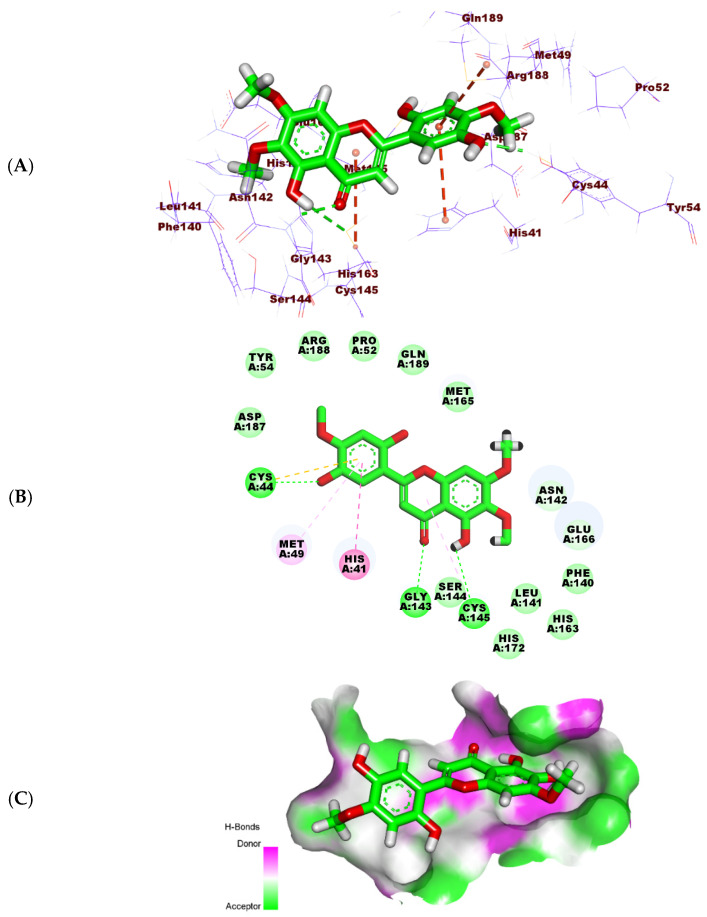
(**A**) Three-dimensional, (**B**) two-dimensional, and (**C**) surface mapping of Jusanin docked into M^pro^.

The binding mode of the Jusanin showed a binding free energy of -19.54 kcal/mol (Table 5). The 2,3-dimethoxyphenol moiety was oriented into the first pocket of the receptor to form one H-bond with Cys145. It showed close contact with Asn142, Glu166, Phe140, His163, and Leu141. In addition, the 4H-pyran-4-one was buried in the second pocket forming one hydrophobic interaction with Cys145. It formed one H-bond with Gly143. The 2-methoxybenzene-1,4-diol moiety occupied the third pocket in close contact with Arg188 and Met49. It formed three hydrophobic bonds with Cys44, Met49, and His41. Additionally, it formed one H-bond with Cys44 (Figure 11).

### 2.7. In Silico ADMET Analysis

**Figure 12:**
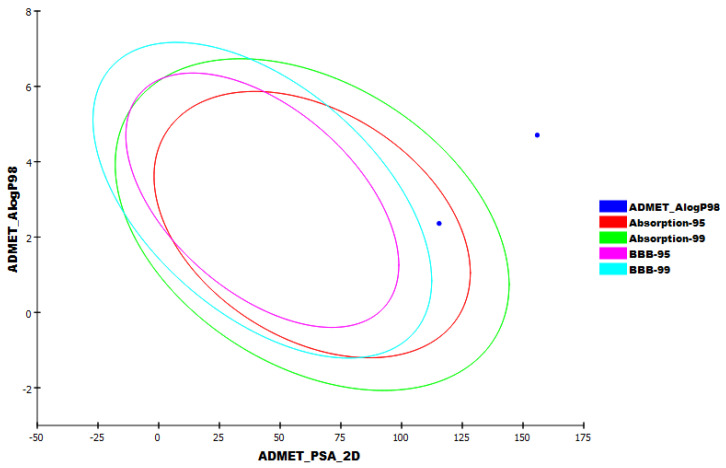
The expected ADMET study. ADMET_AlogP98: lipid-water partition coefficient; ADMET_PSA_2D: polar molecular surface area. Two-dimensional polar surface area (PSA_2D) each drug is plotted against their computed atom-type partition coefficient (ALogP98). The area encompassed by the ellipse represents good absorption without any violation of the ADMET properties. Based on Egan et al. [75] absorption model the 95% and 99% confidence limit ellipses corresponding to the blood–brain barrier (BBB) and intestinal absorption models are indicated.

The possibility of a molecule being a drug depends on its activity as well as its bioavailability [74]. In consequence, the in silico ADMET parameters of Jusanin were determined by Discovery Studio software. Simeprevir was used as a reference drug. The results were illustrated in Table 5 and Figure 12.

The results revealed that Jusanin has a very low chance to penetrate BBB indicating its high safety margin against CNS. Moreover, Jusanin exhibited good aqueous solubility and intestinal absorption. Finally, Jusanin was predicted to be CYP2D6 non-inhibitor and can bind plasma protein by more than 90%.

### 2.8. In Silico Toxicity Studies

The proposed toxicity of Jusanin was determined in silico utilizing Discovery Studio software. Seven different toxicity models were applied using Simeprevir as a reference drug. The results were summarized in Table 6.

FDA rodent carcinogenicity in male mice expected that Jusanin is non-carcinogenic. Additionally, Jusanin showed high carcinogenic potency TD50 value of 13.5782 mg/kg body weight/day, comparing to Simeprevir (0.2803 mg/kg/day). Additionally, Jusanin showed a high rat maximum tolerated dose value of 0.3502 g/kg compared to Simeprevir (0.0030 g/kg body weight). Jusanin showed a rat oral LD50 value of 0.4621 g/kg which was higher than the reference drug LD50 = 0.2088 g/kg. Regarding the rat chronic LOAEL model, Jusanin exhibited a high value of 0.0624 g/kg, higher than Simeprevir (0.0021 g/kg body weight). Finally, Jusanin was predicted to have mild irritancy against the ocular irritancy model and non-irritant against the skin irritancy model.

### 2.9. Molecular Dynamics (MD) Simulations Studies

MD simulation is a powerful accurate in silico method that can explore the exact binding mode, flexibility, and stability of a specific receptor–ligand complex for a certain time [76]. Molecular dynamics (MD) simulations studies were employed to mimic the dynamic nature of M^pro^–Jusanin interaction investigating the simulation for binding complex stability at 100 ns.

**Figure 13:**
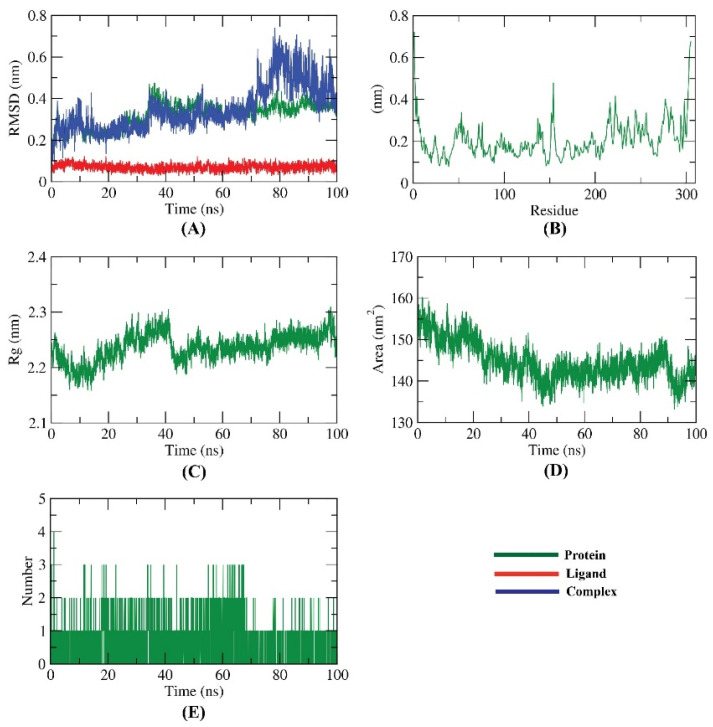
Molecular dynamics simulations results; (**A**) RMSD values of Jusanin, M^pro^, and Jusanin-M^pro^ complex during MD runs. (**B**) RMSF for M^pro^ in the MD run. (**C**) Radius of gyration of M^pro^ in the MD run. (**D**) SASA of M^pro^ in the MD run. (**E**) H-bonding between Jusanin-M^pro^ complex in the MD run.

The dynamic changes of atoms in addition to the conformational variations of atoms backbone in the M^pro^-Jusanin complex were estimated by RMSD to investigate their stability in both apo- and ligand-bonded states. The M^pro^-Jusanin exhibited low RMSD values (less than 0.6 nm) with no major fluctuations declaring the great stability of the complex. The M^pro^-Jusanin complex was stable till 70 ns~. Although the M^pro^-Jusanin complex showed a minor fluctuation later, it started to be more stable again at 85 ns~ (Figure 13A).

The flexibility of M^pro^ was estimated in terms of RMSF to identify the regions of M^pro^ that are being fluctuated through the simulation. Figure 13B shows that the binding of M^pro^ with Jusanin did not change the flexibility of M^pro^. The compactness of the M^pro^-Jusanin was represented by the radius of gyration (R_g_). The decrease in fluctuation throughout the simulation period indicates the higher compactness of the examined system. The R_g_ of the M^pro^-Jusanin complex reached a stable conformation with the radius of gyration fluctuating around 2.2 nm (Figure 13C). The interaction between M^pro^-Jusanin complex and solvents was investigated by solvent accessible surface area (SASA) over the simulation time. Consequently, SASA of the M^pro^-Jusanin complex was calculated to investigate the degree of the conformational changes that happened after the interaction. Interestingly, as shown in Figure 13D, M^pro^ exhibeted a decrease in the surface area showing a comparatively lower value of SASA than the starting period. H-bonding between the M^pro^-Jusanin complex as anessential factor to stabilize the complex observed (Figure 13E). The highest number of conformations of the M^pro^ formed up to three H-bonds with Jusanin.

## 3. Experimental

### 3.1. General Experimental Section

NMR spectra were carried out on a commercial instrument (Bruker Avance 300 and 600 MHz), chemical shifts (δ) are presented in parts per million (ppm) and re-calculated with respect to tetramethylsilane (TMS) (^1^H) or carbon signals of deuterium solvents (^13^C). Spin–spin coupling constants (J) are given in hertz (Hz). The refinement of ^13^C NMR spectra signals was carried out using Dept, HSQC, HMBC NMR spectra. Mass spectra were recorded on an HP5989A instrument (CI and EI, ionization energy 70 eV) with Apollo 300 data, and on a Kratos MS50TC instrument for accurate calculations (reaching by electric shock (ESI), common solvent mixture: CH_2_Cl_2_^−^MeOH^+^ NH_4_OAc) with MASSLYNX system data. UV spectra were obtained on a Perkin-Elmer Lambda 20 Spectrometer instrument. Melting points were determined on Reichert Thermovar. For column chromatography, silica gel 0.06–0.2 mm (Acros) was used as the stationary phase. Silica gel 32–63 mesh was used for flash column chromatography.

### 3.2. Plant Material

To study the component composition of *Artemisia commutata* Besser, or wormwood substitute (family *Asteraceae*), the aboveground part of the plant is collected in the eastern Kazakhstan region (Western Altai Mountains) on July, phase of blooming—beginning of flowering.

Species is identified by botanists of the Altai Botanical Garden (Rider city, eastern Kazakhstan). The herbarium sample is stored in the International Scientific Research Holding «Phytochemistry» Fund. The herbarium sample code is 2007.10.02.02.03.

### 3.3. Extraction and Isolation

In total, 1.04 kg of raw material was placed in a round-bottomed flask and filled with chloroform and heated to the boiling point of solvents. This operation was repeated three times. The solvent was evaporated on a rotary evaporator under the vacuum of a water-jet pump to obtain an extract weighing 20 g, which was used for preparative chromatographic separation by column chromatography on silica gel using Heptane-Ethylacetate-Methanol followed by Sephadex LH-20 (Pharmacia Fine Chemicals), Uppsala, Sweden.

### 3.4. Molecular Similarity

The molecular similarity of Jusanin against the co-crystallized ligands of SARS-Cov-2 was carried out and calculated using Discovery Studio 4.0 [77] (See Supplementary Materials).

### 3.5. Fingerprint Study

A fingerprint study of Jusanin against the nine co-crystallized ligands of SARS-Cov-2 was carried out and calculated using Discovery Studio 4.0 (See Supplementary Materials).

### 3.6. DFT

The DFT parameters of Jusanin were calculated using Discovery Studio software [78] (See Supplementary Materials).

### 3.7. Docking Studies

The docking investigation was carried out for Jusanin using MOE2014 software. The results of the docking process were then visualized using Discovery Studio 4.0 software [79,80,81] (See Supplementary Materials).

### 3.8. ADMET

ADMET descriptors of Jusanin were determined using Discovery Studio 4.0 [82,83] (See Supplementary Materials).

### 3.9. Toxicity Studies

The toxicity parameters of Jusanin were calculated using Discovery Studio 4.0 [84,85,86] (See Supplementary Materials).

### 3.10. Molecular Dynamics Simulations

The system was prepared using the web-based CHARMM-GUI [87,88,89] interface with the CHARMM36 force field [90]. All the simulations were carried out using the NAMD 2.13 [91] package. The TIP3P explicit solvation model and CHARMM general force field were used [92,93] (See Supplementary Materials).

## 4. Conclusions

A new flavonoid, Jusanin, (**1**) has been isolated from the aerial parts of Artemisia commutata. Jusanin showed a high structural similarity degree with **X77**, the co-crystallized ligand of the COVID-19 main protease (PDB ID: 6W63), M^pro^. This result was indicated by molecular similarity, fingerprint, and DFT studies. The molecular docking of **1** against M^pro^ confirmed the correct binding of **1** inside M^pro^ exhibiting a binding energy of −19.54 Kcal/mol. ADMET and toxicity properties of 1 indicated its likeness to be a drug as well as its general safety. The MD simulation studies at 100 ns confirmed the correct binding of the M^pro^-Jusanin complex. These interesting results open the door to finding a treatment against COVID-19 after in vitro and in vivo studies.

## Figures and Tables

**Table 1:** ^1^H and ^13^C spectral data of Jusanin (DMSO).

Position	^1^H (*J* = Hz)	^13^С	Position	^1^H (*J* = Hz)	^13^С
2		161.97	2′		152.4
3	7.11 s	106.71	3′	6.58 s	104.39
4		182.22	4′		142.0
5		152.6	5′		139.7
6		131.7	6′	7.45 s	111.94
7		158.45	6-OCH_3_	3.74 s	60.0
8	6.97 s	91.57	7-OCH_3_	3.82 s	56.7
9		151.95	4′-OCH_3_	3.94 s	56.5
10		105.3	5-OH	13.06 s	
1′		107.24			

**Table 2:** Structural properties of Jusanin and the different co-crystallized ligands of SARSCoV-2 proteins.

Compound	ALog p	M. Wt	HBA	HBD	Rotatable Bonds	Rings	Aromatic Rings	MFPSA	Minimum Distance
**Jusanin**	2.361	360.315	8	3	4	3	2	0.325	0
**X77**	2.622	403.477	4	2	6	4	3	0.22	0.92
**TTT**	3.647	304.386	2	2	3	3	3	0.171	0.95
**VXG**	0.711	233.263	3	1	2	2	1	0.237	1.10
**F86**	−1.502	371.243	11	5	4	3	2	0.612	1.12
**SAM**	−4.254	399.445	9	4	7	3	2	0.483	1.17
**GWS**	2.171	218.295	2	1	3	2	1	0.179	1.20
**PRD_002214**	2.453	680.791	8	5	18	3	2	0.273	1.38
**1N7**	0.231	631.884	8	6	12	4	0	0.256	1.52

**Table 3:** Fingerprint similarity between Jusanin and the different co-crystallized ligands of SARSCoV-2 proteins.

Comp.	Similarity	SA	SB	SC
Jusanin	1	331	0	0
**X77**	0.512	266	188	65
**TTT**	0.489	258	196	73
**VXG**	0.436	151	15	180
**F86**	0.339	136	70	195
**GWS**	0.310	120	56	211
**SAM**	0.299	131	106	200
**PRD_002214**	0.291	327	789	4
**1N7**	0.138	216	1231	115

**Table 4:** MO spatial distribution of Jusanin and **X77**.

Name	Binding Energy (Ha)	HOMO Energy (Ha)	LUMO Energy (Ha)	Dipole Mag	Band Gap Energy (Ha)
Jusanin	−8.558	−0.180	−0.098	2.395	0.083
**X77**	−10.830	−0.158	−0.062	2.906	0.096

**Table 5:** Predicted ADMET for Jusanin and reference.

Comp.	BBB Level ^a^	Solubility Level	Absorption Level	CYP2D6 Prediction ^b^	PPB Prediction ^c^
Jusanin	V. low	good	good	non inhibitor	>90%
Simeprevir	V. low	low	V. poor	non inhibitor	>90%

^a^ BBB level, blood–brain barrier penetration level; ^b^ CYP2D6, cytochrome P2D6 inhibition; ^c^ PBB, plasma protein binding level.

**Table 6:** Toxicity properties of Jusanin and reference.

Comp.	FDA Rodent Carcinogenicity (Mouse-Male)	Carcinogenic Potency TD_50_ (Rat) ^a^	Rat Maximum Tolerated Dose (Feed) ^b^	Rat Oral LD_50_ ^b^	Rat Chronic LOAEL ^b^	Ocular Irritancy	Skin Irritancy
Jusanin	Non-Carcinogen	13.5782	0.3502	0.4621	0.0624	Mild	Non-Irritant
Simeprevir	Non-Carcinogen	0.2803	0.0030	0.2088	0.0021	Mild	Non-Irritant

^a^ Unit: mg/kg body weight/day ^b^ Unit: g/kg body weight.

## Data Availability

All data is contained in the published article.

## Supplementary Materials

The following data are available online, 1D, 2D NMR and HR-Ms spectra data of Jusanin, detailed toxicity report of Jusanin, in addition to the utilized in silico methods.

## Sample Availability

Samples of the compounds are available from the authors.
