# Supplementary material for: Jusanin, a New Flavonoid from Artemisia commutata with an In Silico Inhibitory Potential against the SARS-CoV-2 Main Protease
Source: Molecules. 2022 Mar 1;27(5):1636. doi: 10.3390/molecules27051636 (PMC8911936; doi:10.3390/molecules27051636)
Supplement: Supplementary file 1 [file molecules-1583631-supplementary.pdf › molecules-1583631-supplementary.pdf]

**Supporting Data**

# Jusanin, a new flavonoid from *Artemisia commutata* with an *in silico* inhibitory potential against SARS-CoV-2 main protease

**Yerlan M. Suleimen [1,2], Rani A. Jose [3], Raigul N. Suleimen[4*], Christoph Arenz[5], Margarita Yu. Ishmuratova [6], Toppet Suzanne[3], Wim Dehaen [3], Bshra A. Alsfouk [7], Eslam B. Elkaeed [8] Ibrahim. H. Eissa[9], Ahmed M. Metwaly[10,11*]**

[1]The International Centre for Interdisciplinary Solutions on Antibiotics and Secondary Metabolites, Republican collection of microorganisms, Nur-Sultan, Republic of Kazakhstan

[3]Catholic University of Leuven, Leuven, Belgium

[4]L.N. Gumilyov Eurasian National University, 010010, Nur-Sultan, Kazakstan

[5]Institut für Chemie der Humboldt-Universität zu Berlin, Germany

[6]Department of Botany, E.A. Buketov Karaganda University, Karaganda, Republic of Kazakhstan

[7] Department of Pharmaceutical Sciences, College of Pharmacy, Princess Nourah bint Abdulrahman University, P.O. Box 84428, Riyadh 11671, Saudi Arabia

[8]Department of Pharmaceutical Sciences, College of Pharmacy, AlMaarefa University, Ad Diriyah 13713, Riyadh, Saudi Arabia

[9]Pharmaceutical Medicinal Chemistry & Drug Design Department, Faculty of Pharmacy (Boys), Al-Azhar University, Cairo11884, Egypt

[10]Pharmacognosy and Medicinal Plants Department, Faculty of Pharmacy (Boys), Al-Azhar University, Cairo, Egypt

[11]Biopharmaceutical Product Research Department, Genetic Engineering and Biotechnology Research Institute, City of Scientific Research and Technological Applications, Alexandria, Egypt

**Content**


| | |
|---|---|
| Method | Fingerprint studies |
| | Molecular Similarity |
| | Docking studies |
| | ADMET studies |
| | Toxicity studies |
| | Molecular dynamics |
| Spectral data | |
| Toxicity report | |

# Method

## 1. General experimental section

NMR spectra were carried out on a commercial instrument (Bruker Avance 300 and 600 MHz), chemical shifts (δ) are presented in parts per million (ppm) and re-calculated with respect to tetramethylsilane (TMS) ($^{1}$H) or carbon signals of deuterium solvents ($^{13}$C). Spin-spin coupling constants (J) are given in hertz (Hz). Refinement of $^{13}$C NMR spectra signals was carried out using Dept, HSQC, HMBC NMR spectra. Mass spectra were recorded on an HP5989A instrument (CI and EI, ionization energy 70 eV) with Apollo 300 data, and on a Kratos MS50TC instrument for accurate calculations (reaching by electric shock (ESI), common solvent mixture: $CH_2Cl_2$-MeOH + $NH_4OAc$) with MASSLYNX system data. UV spectra were obtained on a Perkin-Elmer Lambda 20 Spectrometer instrument. Melting points were determined on Reichert Thermovar. For column chromatography, silica gel 0.06-0.2 mm (Acros) was used as the stationary phase. Silica gel 32-63 mesh was used for flash column chromatography.

## 2. Plant material

To study the component composition of *Artemisia commutata* Besser, family *Asteraceae*, the aboveground part of plant is collected in the Eastern-Kazakhstan region (Western Altai Mountains) on July, phase of blooming – beginning of flowering.

Species is identified by botanists of the Altai Botanical Garden (Rider city, eastern Kazakhstan). The herbarium sample is stored in the International Scientific Research Holding «Phytochemistry» Fund. The herbarium sample code is 2007.10.02.02.03.

## 3. Extraction and isolation

1.04 kg of raw material was placed in a round-bottomed flask and filled with chloroform and heated to the boiling point of solvents. This operation was repeated three times. The solvent was evaporated on a rotary evaporator under the vacuum of a water-jet pump to obtain an extract weighing 20 g, which was used for preparative chromatographic separation by column chromatography on silica gel.

## 4. Molecular Similarity

Molecular Similarity of the tested compound against the eight co-crystallized ligands of SARS-Cov-2 was carried out calculated using Discovery studio 4.0. At first, the CHARMM force field was applied then the compound was prepared using prepare ligand protocol. Then, the tested compound was used as a reference compound while the co-crystallized ligands were used as a test

set. The protocol was adjusted to give one  output. The default molecular properties were applied. The molecular properties include the number of rotatable bonds, number of rings, number of aromatic rings, number of hydrogen bond donors (HBA), number of hydrogen bond acceptors (HBD), partition coefficient (ALog p), molecular weight (M. Wt), and molecular fractional polar surface area (MFPSA).

**5. Fingerprint study**

A fingerprint study of the tested compound against the eight co-crystallized ligands of SARS-Cov-2 was carried out calculated using Discovery studio 4.0. At first, the CHARMM force field was applied then the compounds were prepared using prepare ligand protocol. Then, the tested compound was used as a reference compound while the co-crystallized ligands was used as a test set. The protocol was adjusted to give the most related co-crystallized ligand s to the tested compound. The default molecular properties were applied. The used fingerprints were based on some parameters related to the type of atoms which may be one of the following: charge, hybridization, H-bond acceptor, H-bond donor, Positive ionizable, Negative ionizable, Halogen, Aromatic, or None of the above. In addition, it includes the ALogP category of atoms.

**6. DFT**

The DFT parameters (total energy, binding energy, HOMO, LUMO, gap energy, dipole moment, and electrostatic potential) were calculated using Discovery studio software. the tested compounds were prepared using prepare ligand protocol. Then, the prepared compounds were subjected to DFT calculation protocol using the default option

**7. Docking studies**

Crystal structure of SARS-Cov-2 main protease (PDB ID: 6W63) was obtained from Protein Data Bank. The docking investigation was accomplished using MOE2014 software. At first, the crystal structure of SARS-Cov-2 main protease was prepared by removing water molecules. Only one chain was retained beside the crystallized ligand, **X77,** (N-(4-tert-butylphenyl)-N-[(1R)-2-(cyclohexylamino)-2-oxo-1-(pyridin-3-yl)ethyl]-1H-imidazole-4-carboxamide). Then, the selected chain was protonated and subjected to minimization of energy process. Next, the active site of the target protein was defined.

Structures of the tested compounds and the co-crystallized ligand were drawn using ChemBioDraw Ultra 14.0 and saved as MDL-SD format. Such file was opened using MOE to display the 3D structures which were protonated and subjected to energy minimization. Formerly,

validation of the docking process was performed by docking the co-crystallized ligand against the isolated pocket of the active site. The produced RMSD value indicated the validity of the process. Finally, docking of the tested compounds was done through the dock option inserted in compute window. For each docked molecule, 30 docked poses were produced using ASE for scoring function and force field for refinement. The results of the docking process were then visualized using Discovery Studio 4.0 software.

**8. ADMET**

ADMET descriptors (absorption, distribution, metabolism, excretion and toxicity) of the compounds were determined using Discovery studio 4.0. At first, the CHARMM force field was applied then the tested compounds were prepared and minimized according to the preparation of small molecule protocol. Then ADMET descriptors protocol was applied to carry out these studies.

**9. Toxicity studies**

The toxicity parameters of the tested compounds were calculated using Discovery studio 4.0. Indinavir was used as a reference drug. At first, the CHARMM force field was applied then the compounds were prepared and minimized according to the preparation of small molecule protocol. Then different parameters were calculated from the toxicity prediction (extensible) protocol.

**10. Molecular dynamics simulations**

The system was prepared using the web-based CHARMM-GUI interface with the CHARMM36 force field. All the simulations were done using the NAMD 2.13 package. The TIP3P explicit solvation model was used[78], and the periodic boundary conditions were set with a dimension of the dimensions ---- Å, ----- Å, and ----- Å in x, y, and z, respectively. The parameters for the top docking results were generated using the CHARMM general force field Afterward, the system was neutralized using ---- ($Cl^-/Na^+$) ions. The MD protocols involved minimization, equilibration, and production. a 2 *fs* time step of integration was chosen for all MD simulations, the equilibration was carried in the canonical (*NVT*) ensemble, while the isothermal–isobaric (*NPT*) ensemble was for the production. Through the 100 *ns* of MD production, the pressure was set at 1 atm using the Nose´–Hoover Langevin piston barostat[80,81] with a Langevin piston decay of 0.05 *ps* and a period of 0.1 *ps*. The temperature was set at 298.15 K using the Langevin thermostat[82]. A distance cutoff of 12.0 Å was applied to short-range nonbonded interactions with

a pair list distance of 16 Å, and Lennard Jones interactions were smoothly truncated at 8.0 Å. Long-range electrostatic interactions were treated using the particle-mesh Ewald (PME) method[83,84], where a grid spacing of 1.0 Å was used for all simulation cells. All covalent bonds involving hydrogen atoms were constrained using the SHAKE algorithm[85]. For consistency, we have applied the same protocol for all MD simulations.

# Spectral data

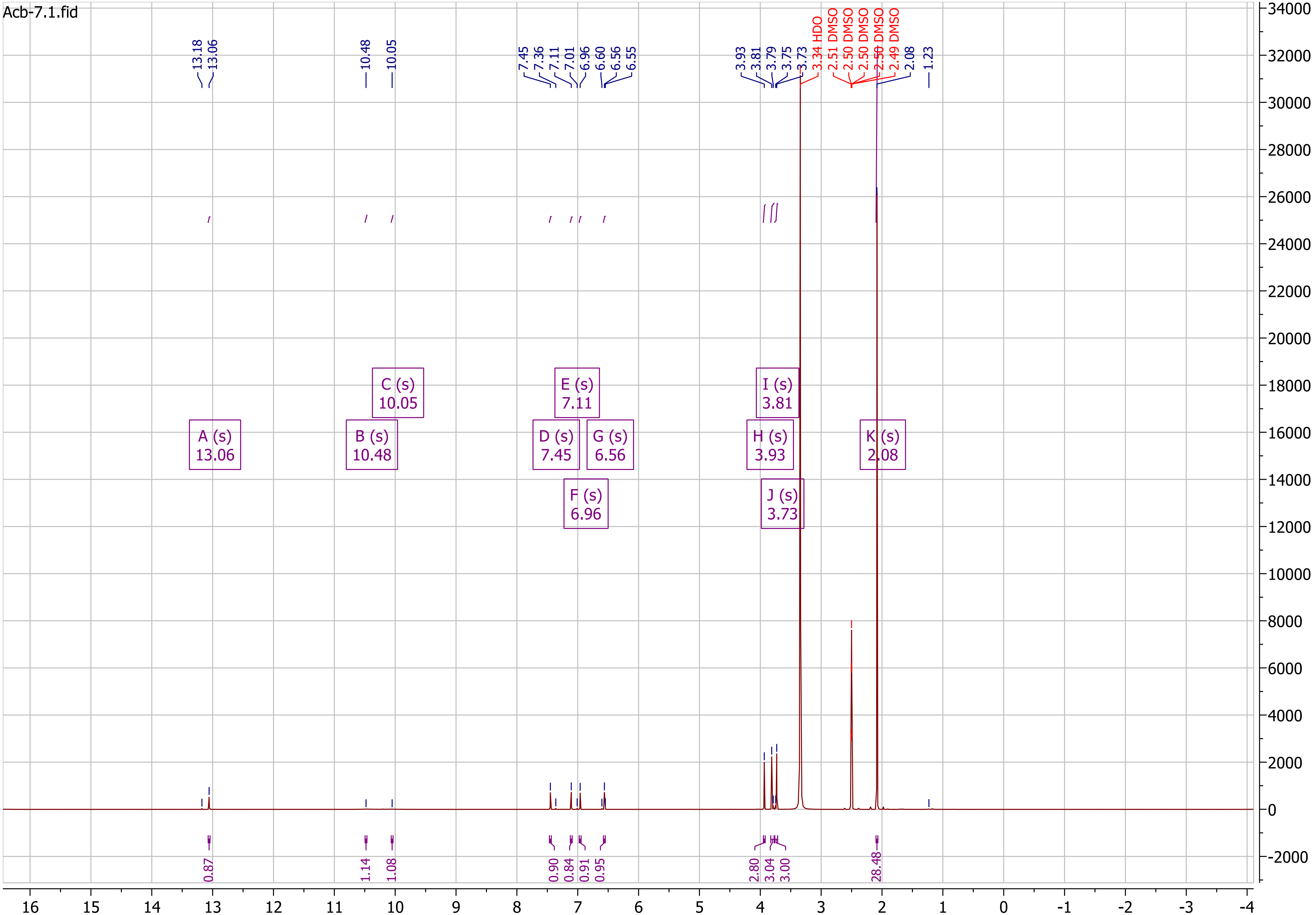


Figure S.1 $^{1}$HNMR of **1**

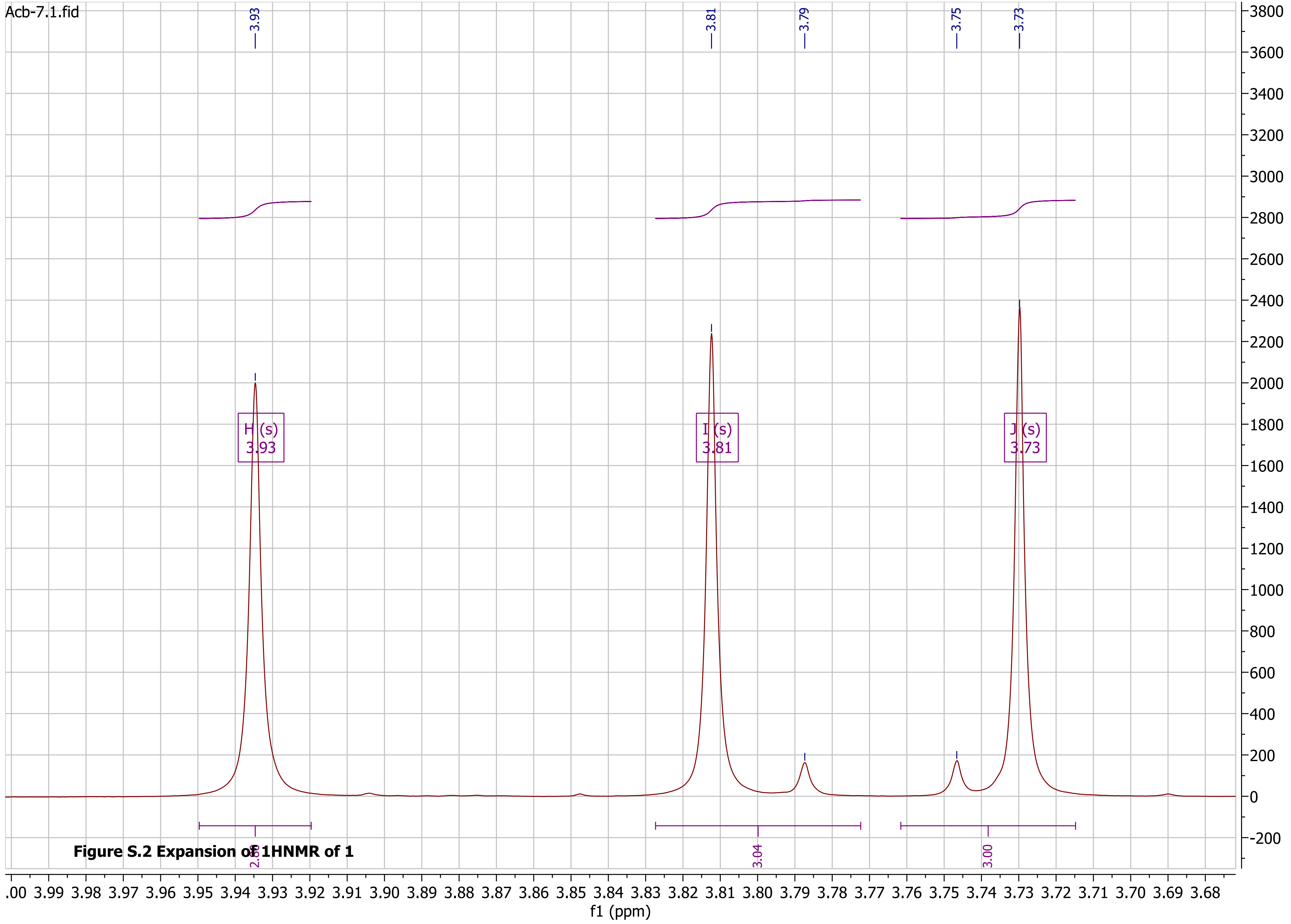


**Figure S.2 Expansion of 1HNMR of 1**

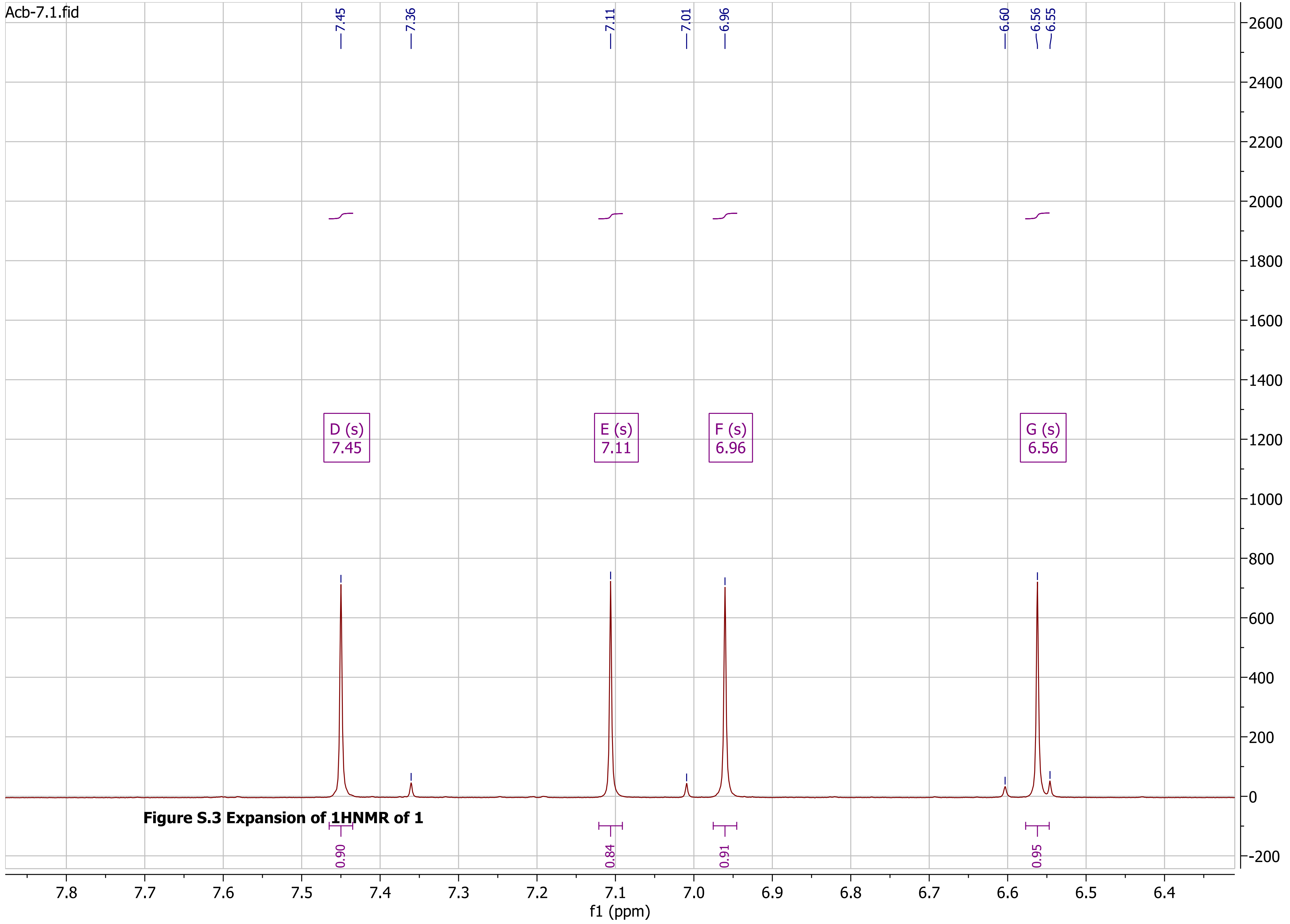


**Figure S.3 Expansion of 1HNMR of 1**

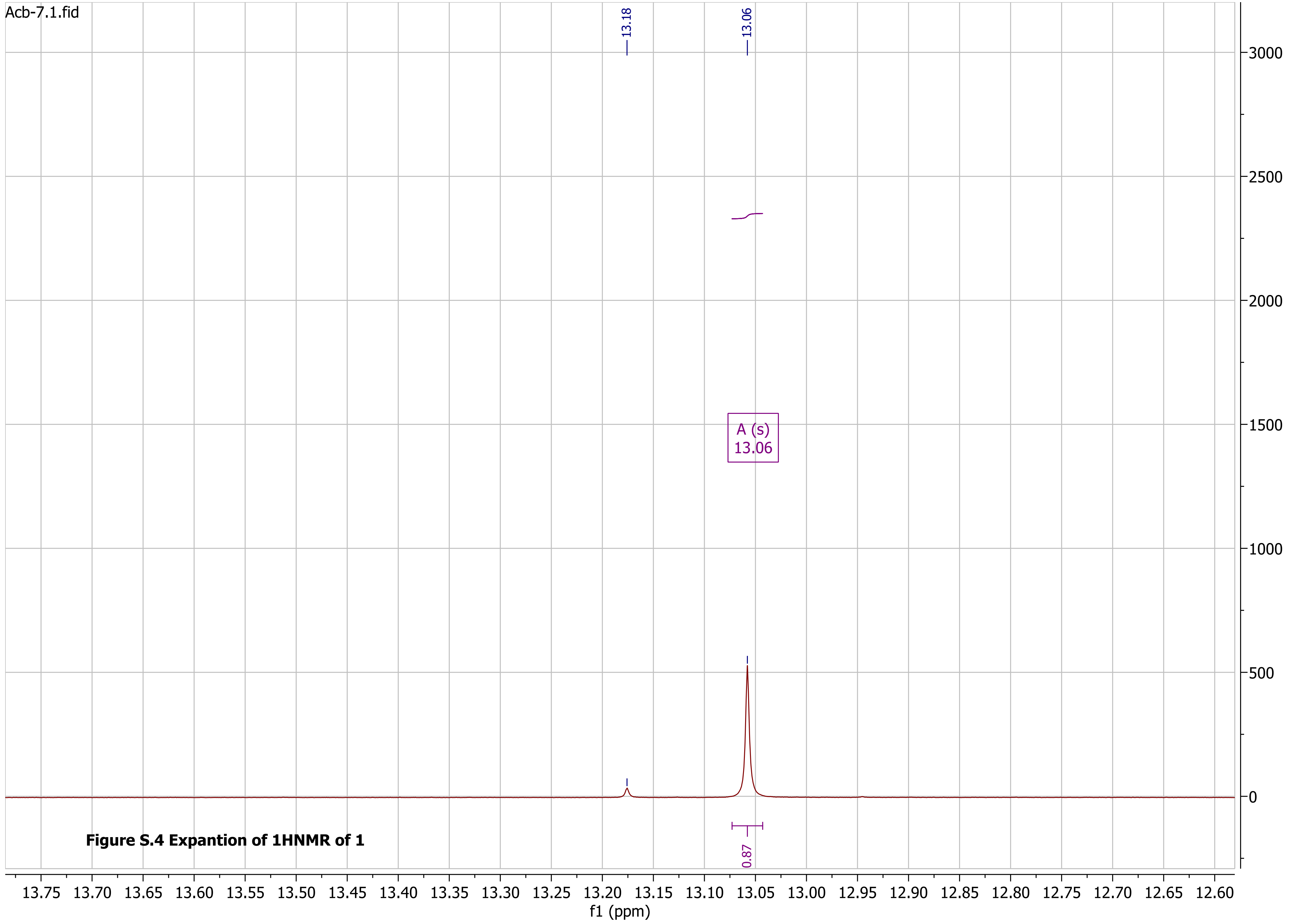


**Figure S.4 Expantion of 1HNMR of 1**

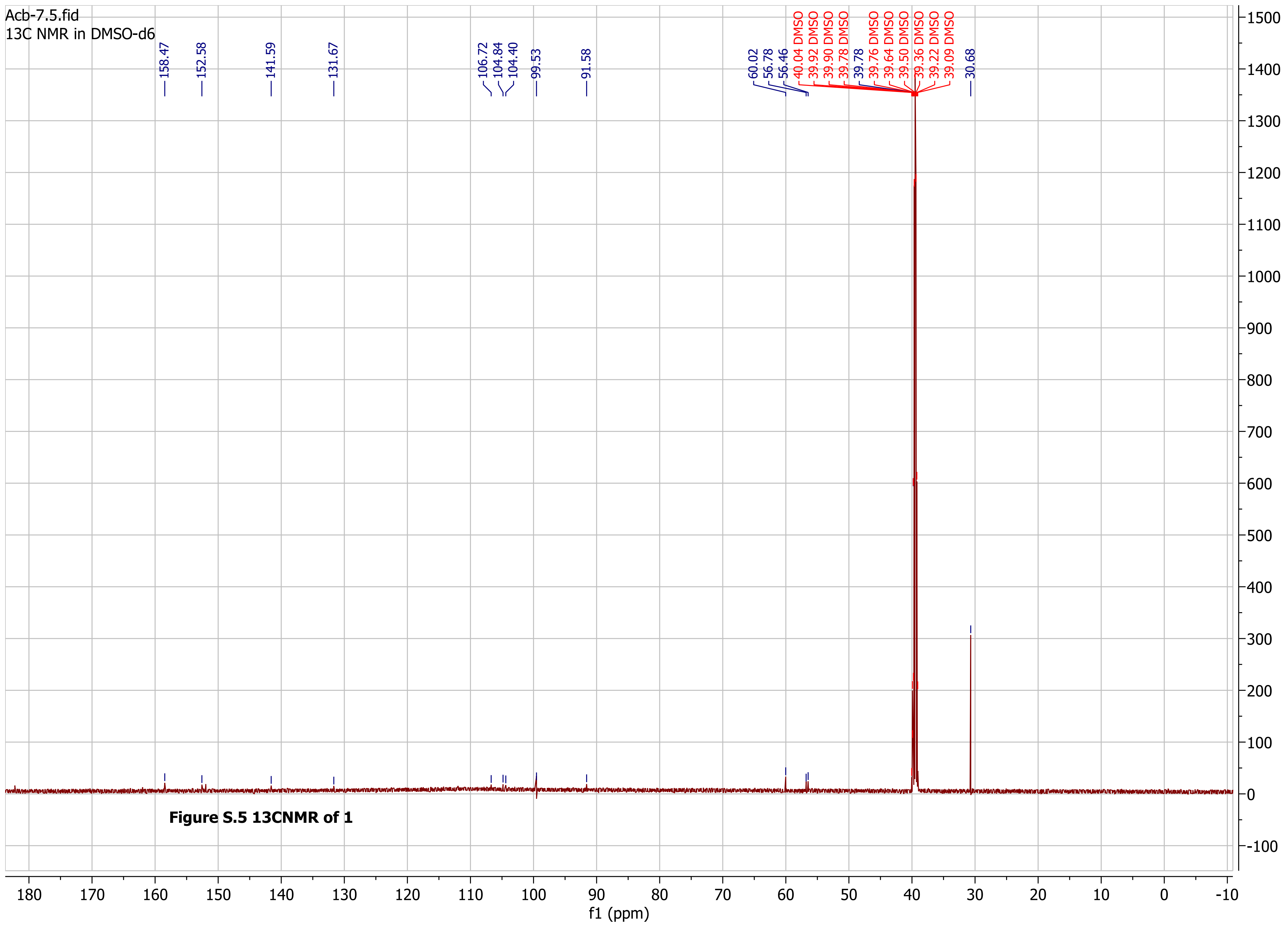


**Figure S.5 13CNMR of 1**

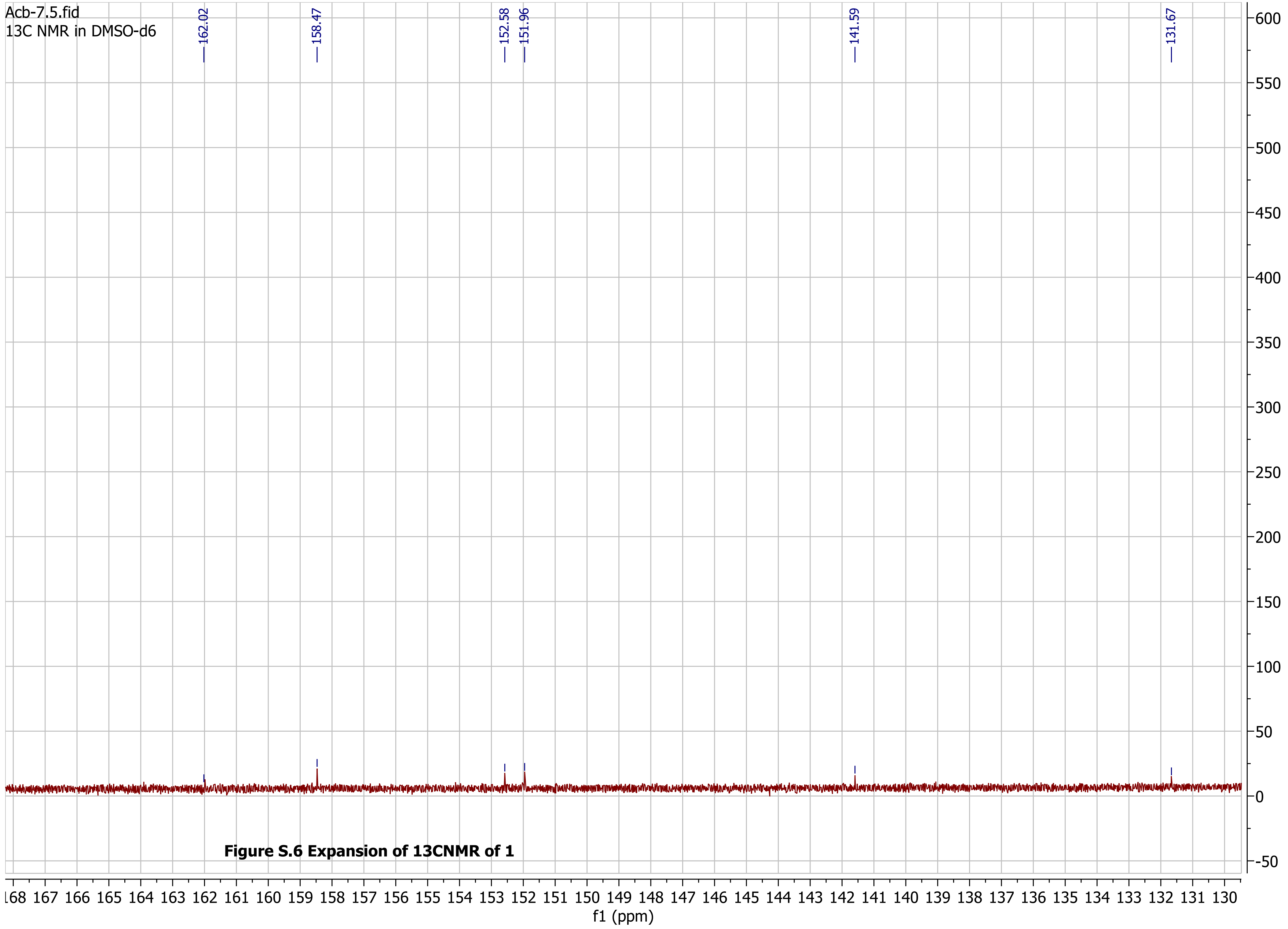


**Figure S.6 Expansion of 13CNMR of 1**

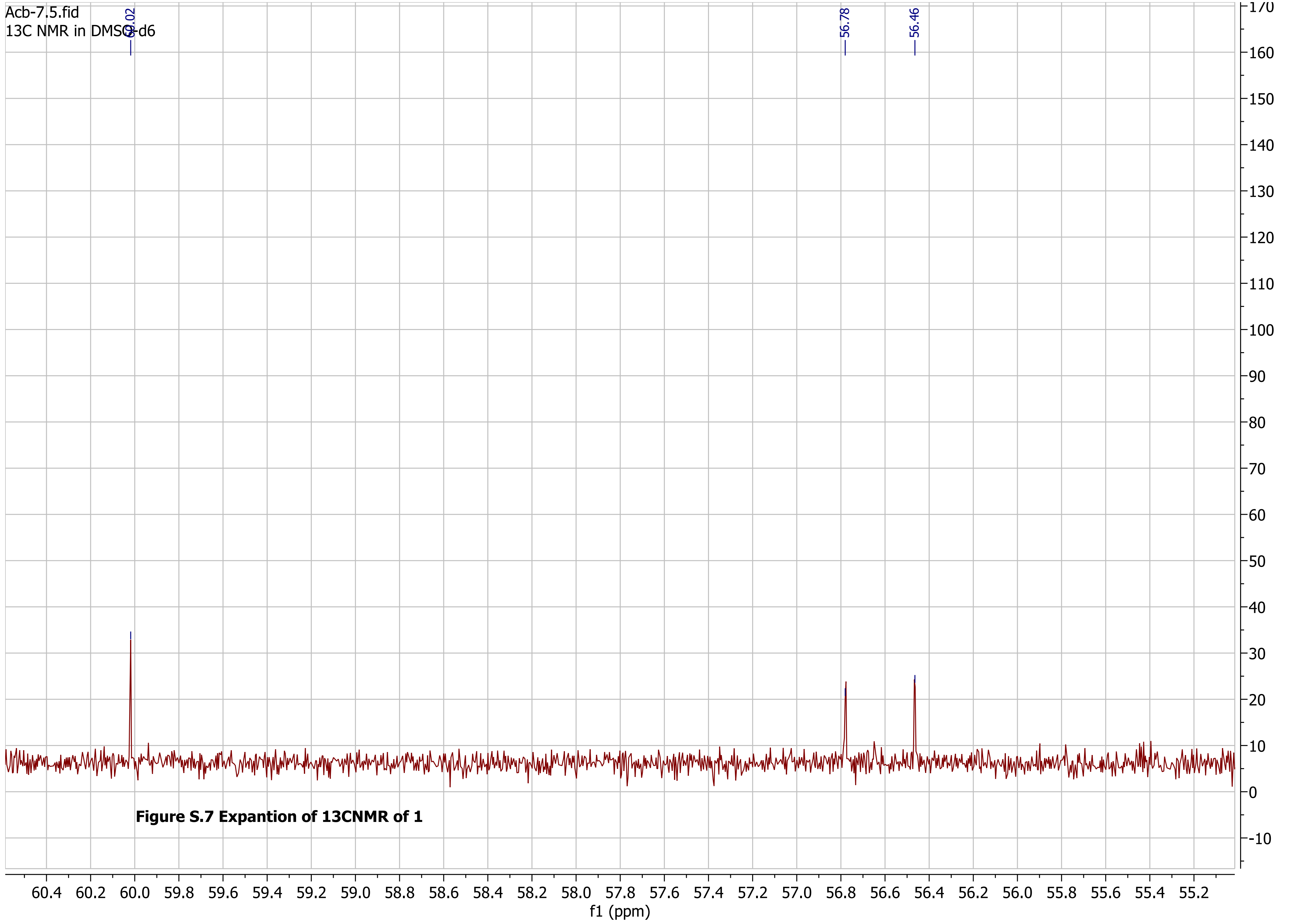


**Figure S.7 Expantion of 13CNMR of 1**

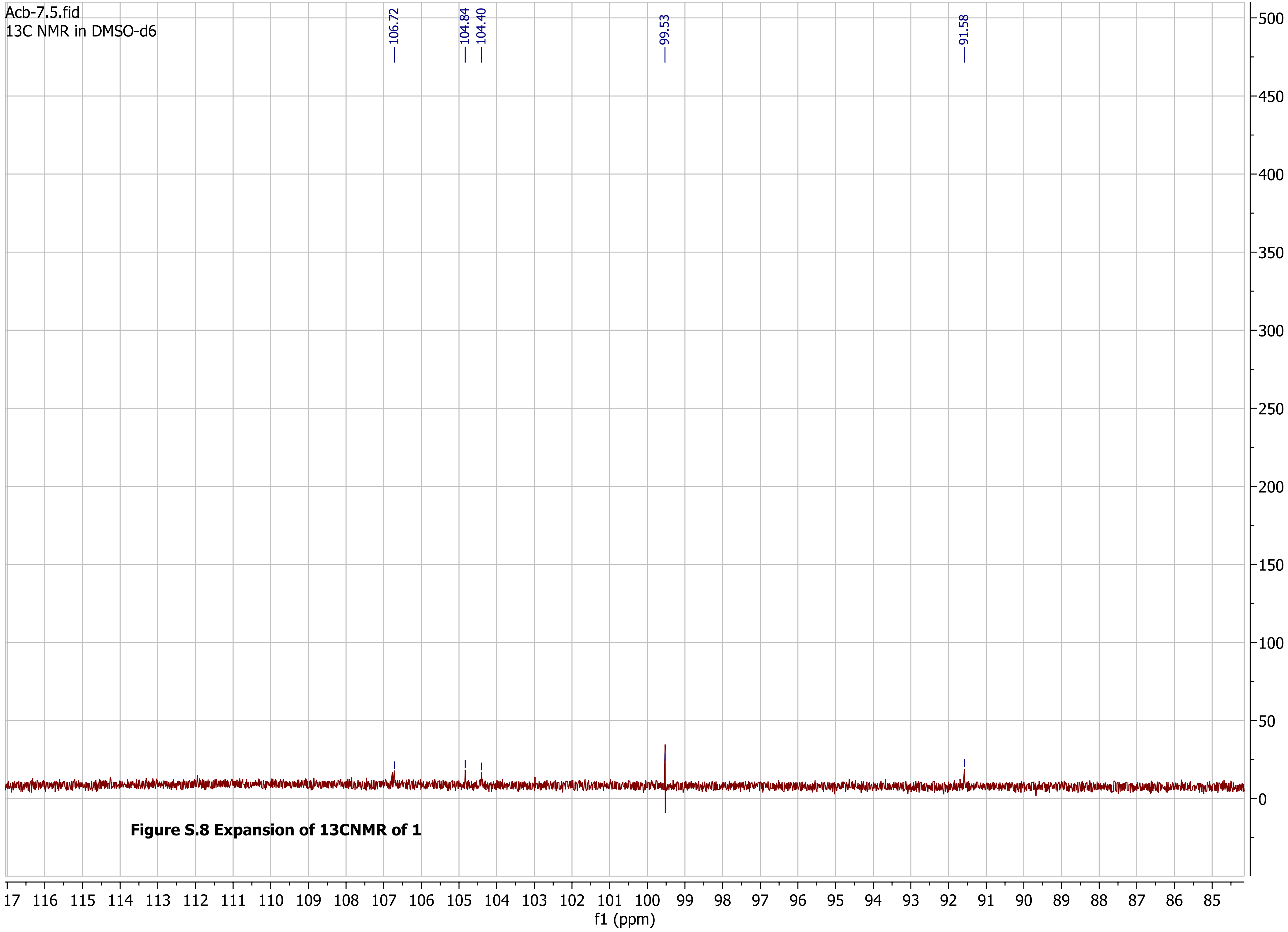


**Figure S.8 Expansion of 13CNMR of 1**

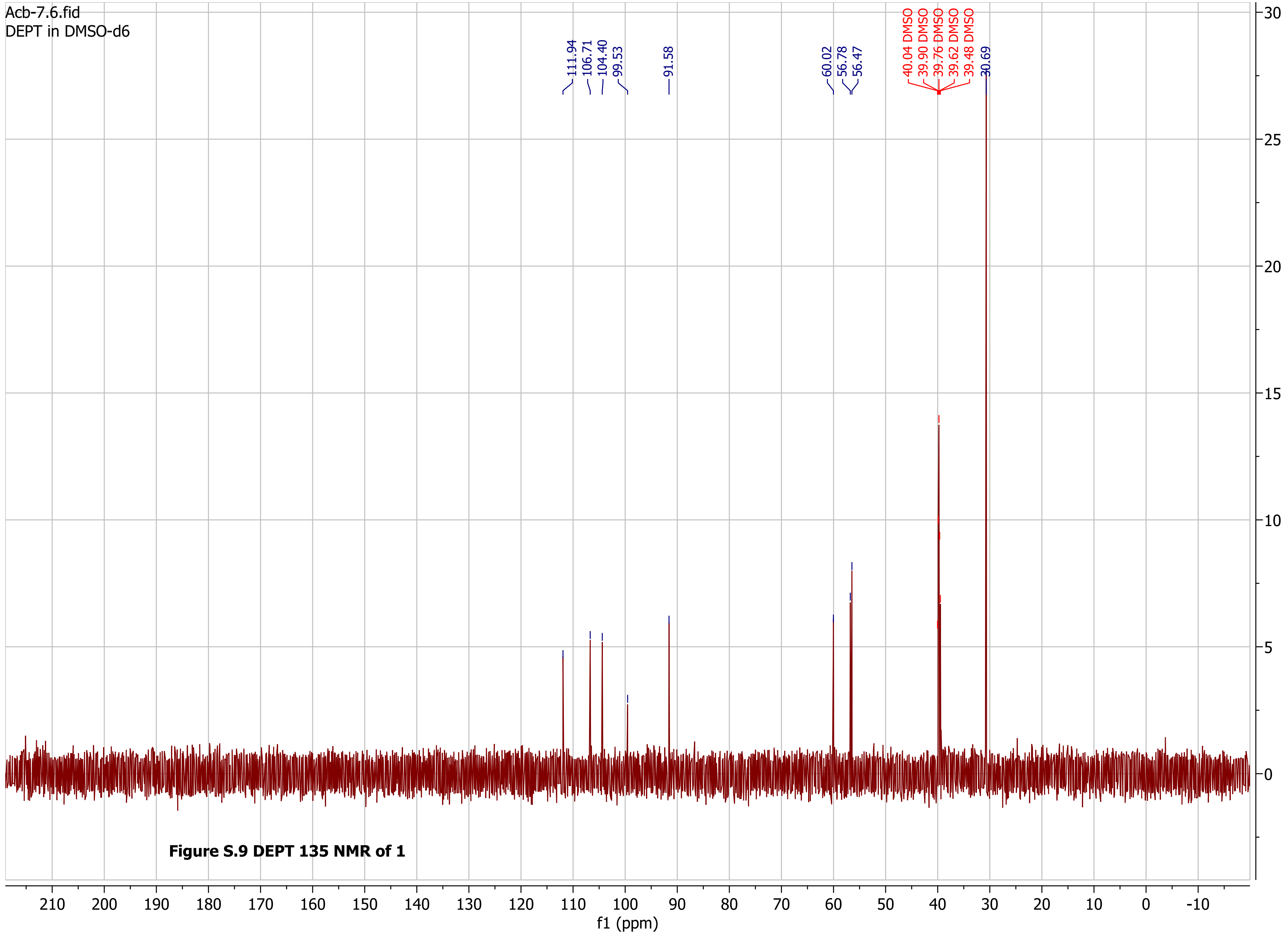


**Figure S.9 DEPT 135 NMR of 1**

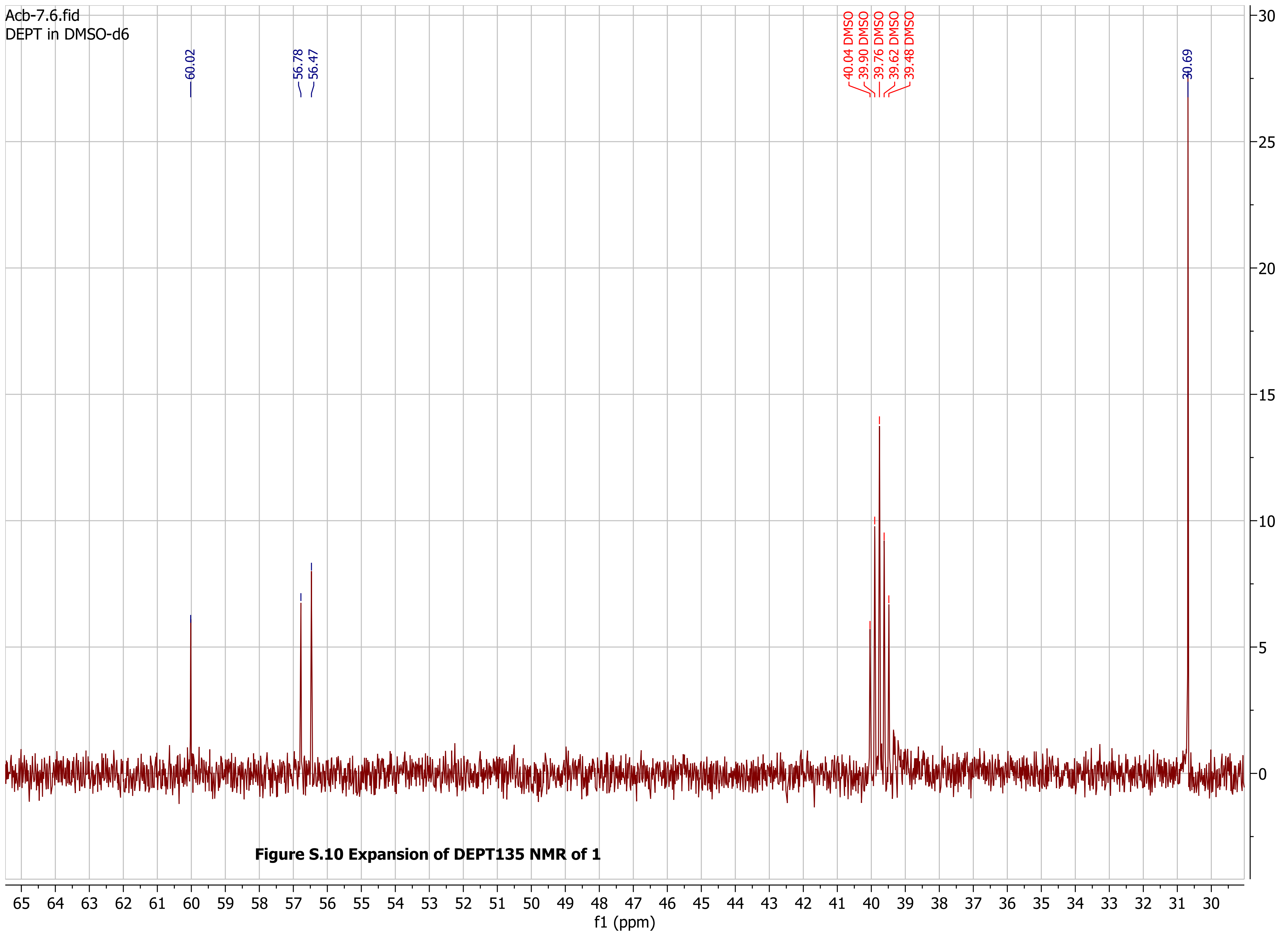


**Figure S.10 Expansion of DEPT135 NMR of 1**

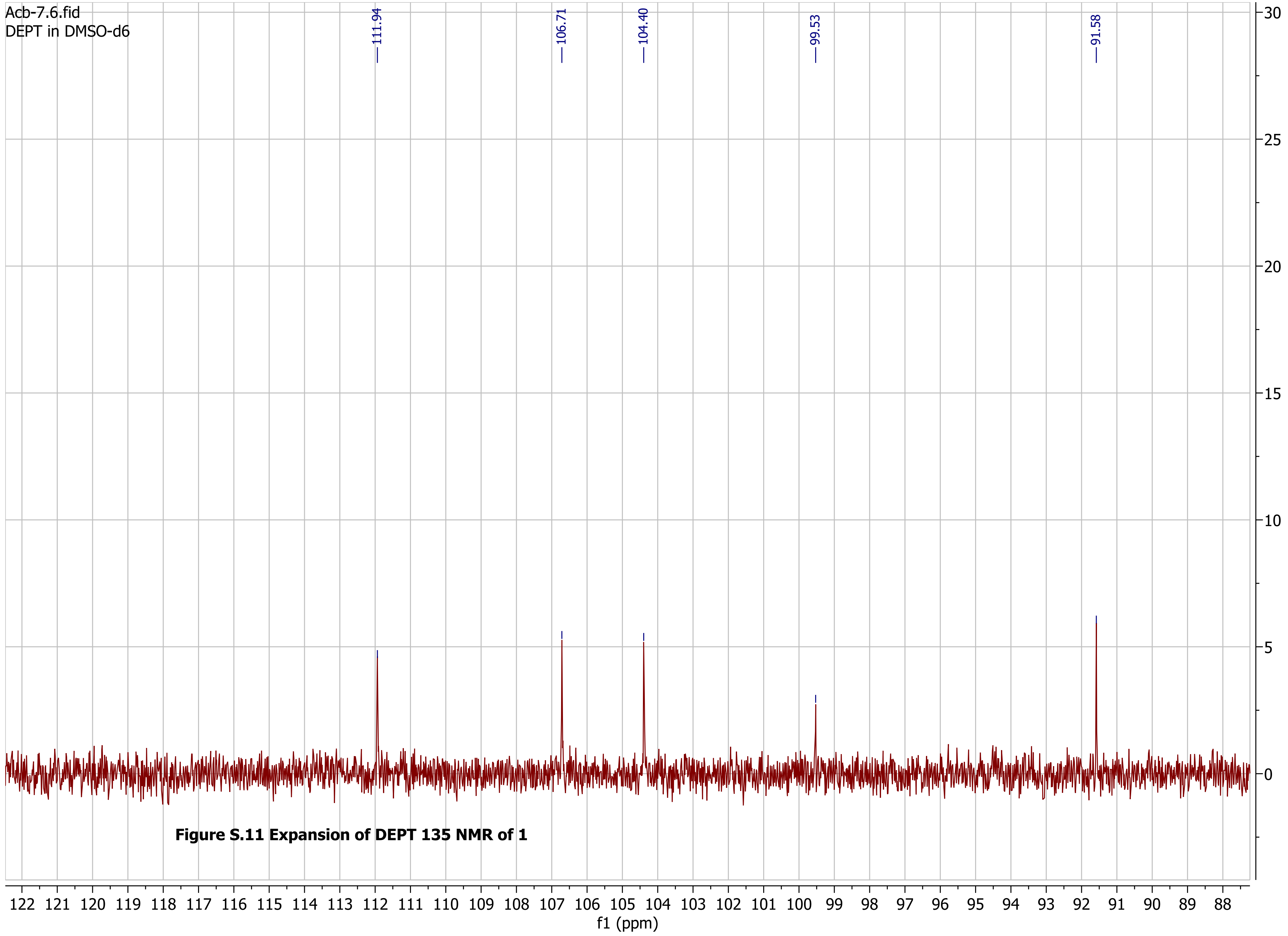


**Figure S.11 Expansion of DEPT 135 NMR of 1**

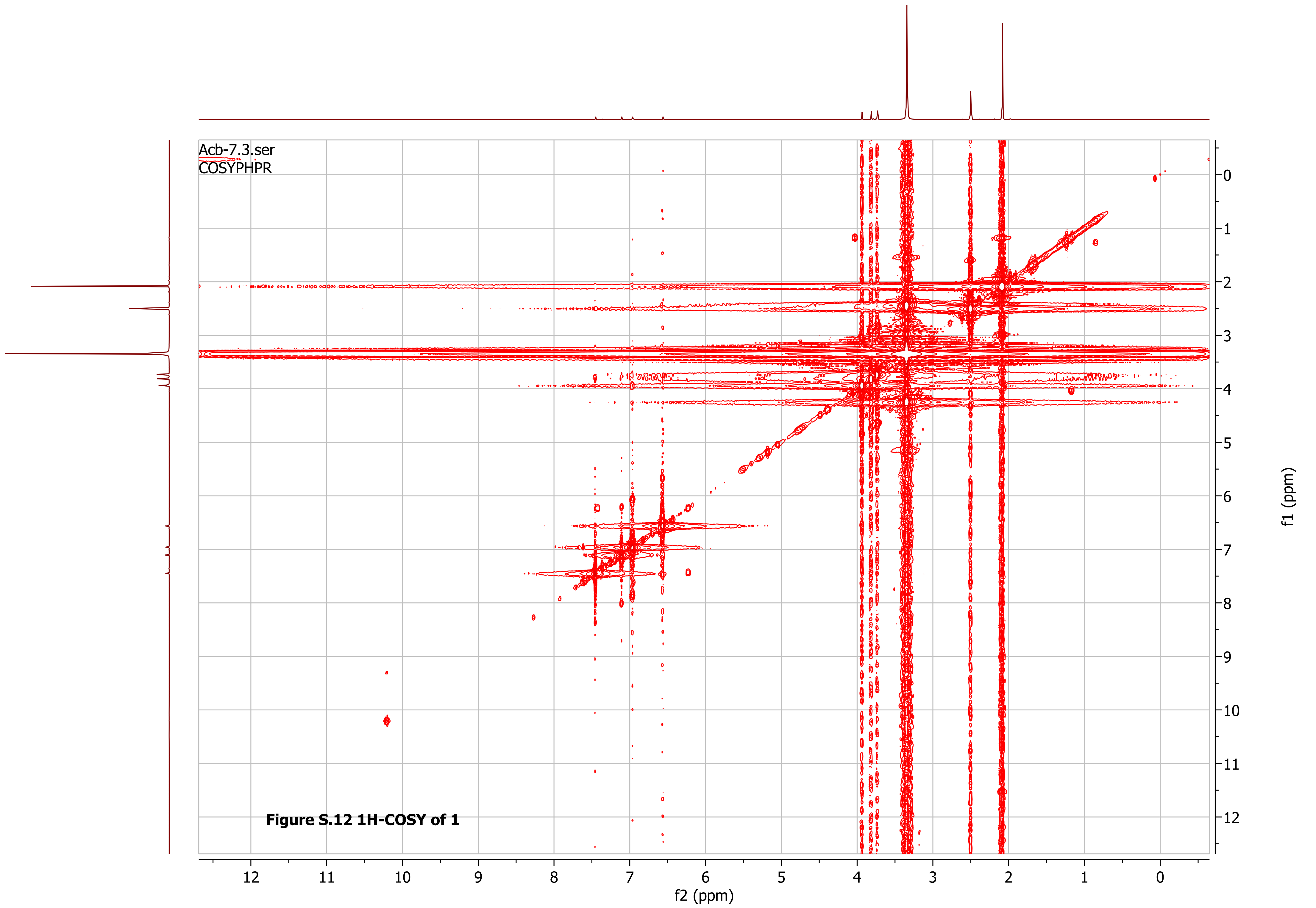


**Figure S.12 1H-COSY of 1**

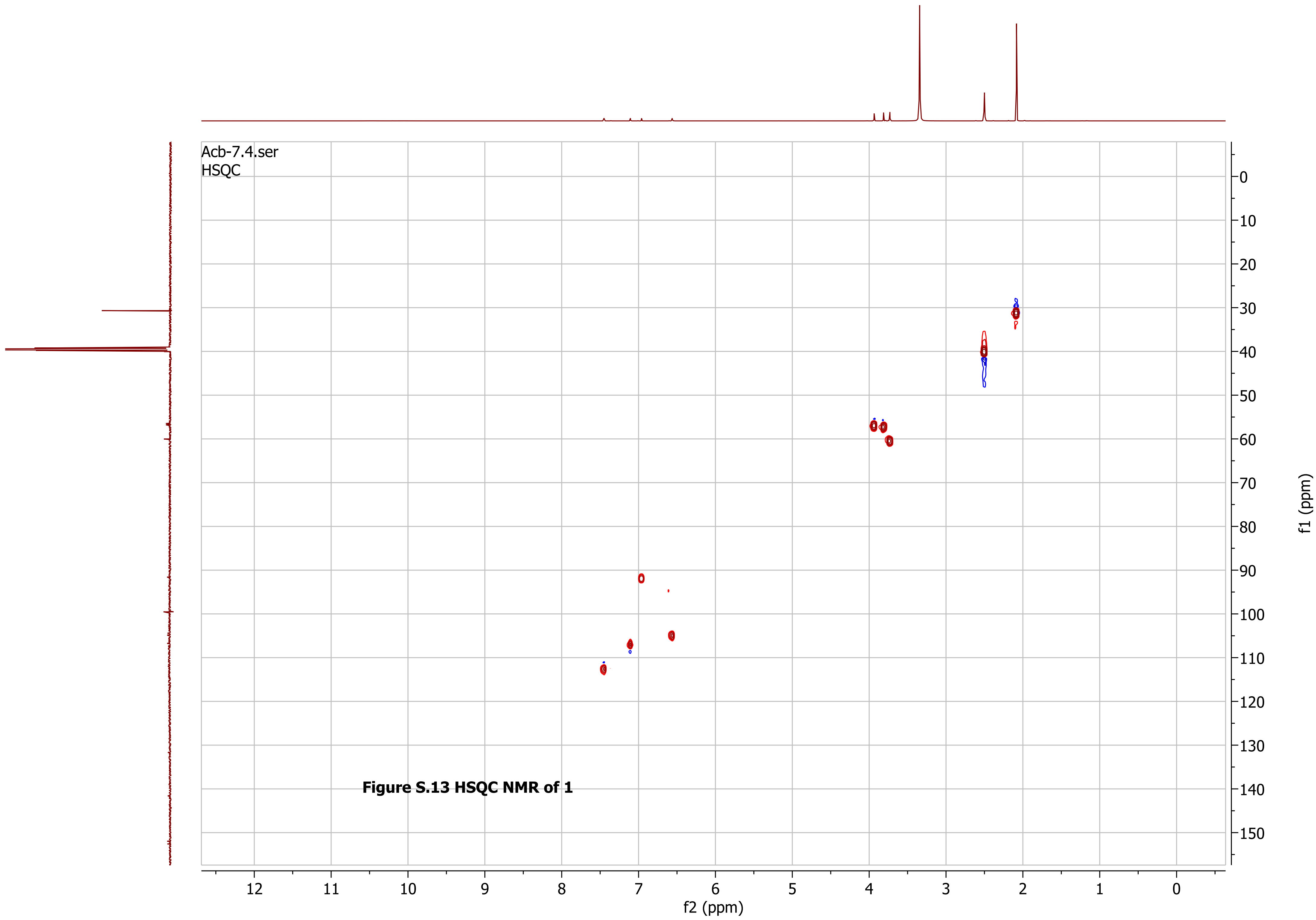


**Figure S.13 HSQC NMR of 1**

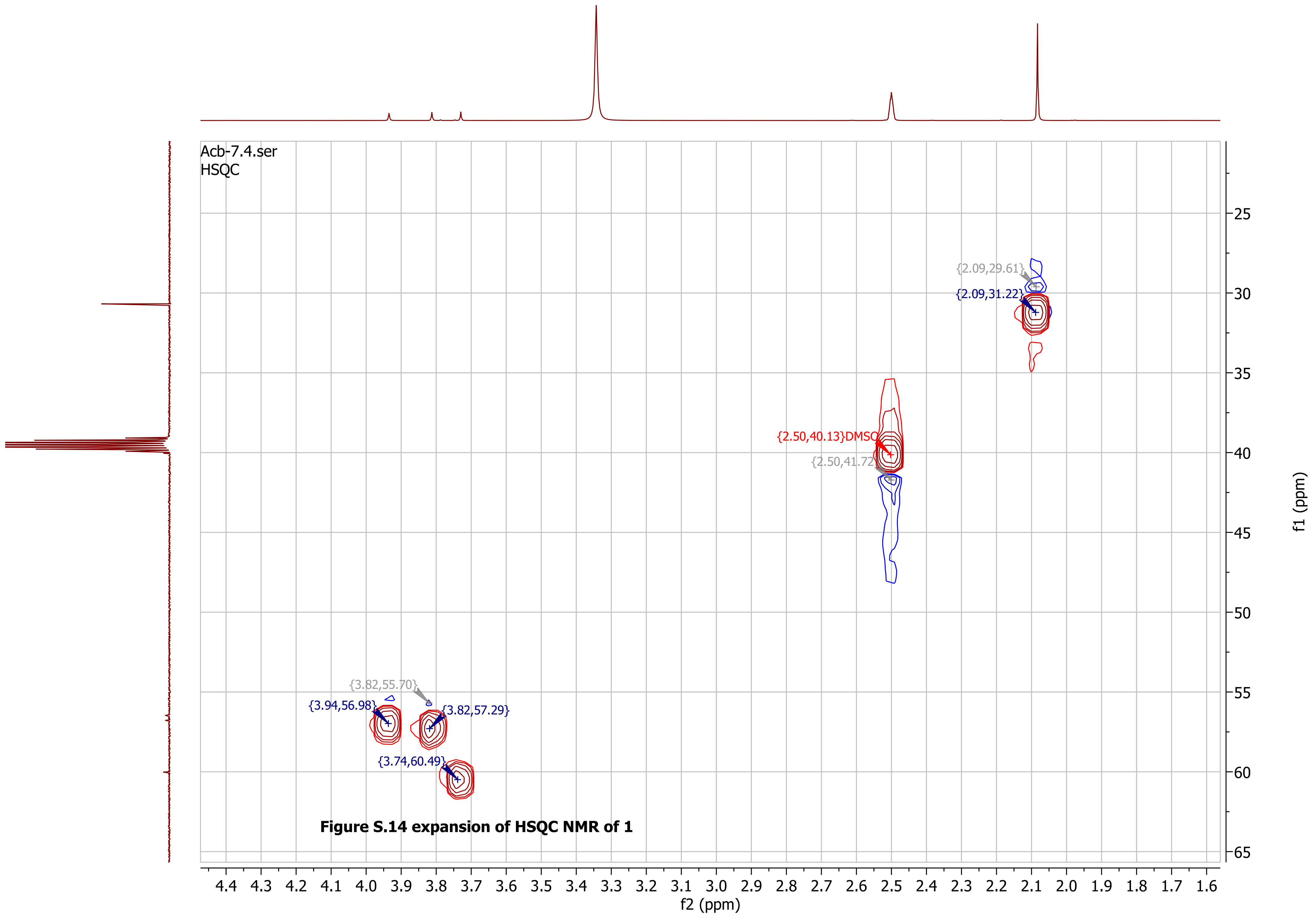


**Figure S.14 expansion of HSQC NMR of 1**

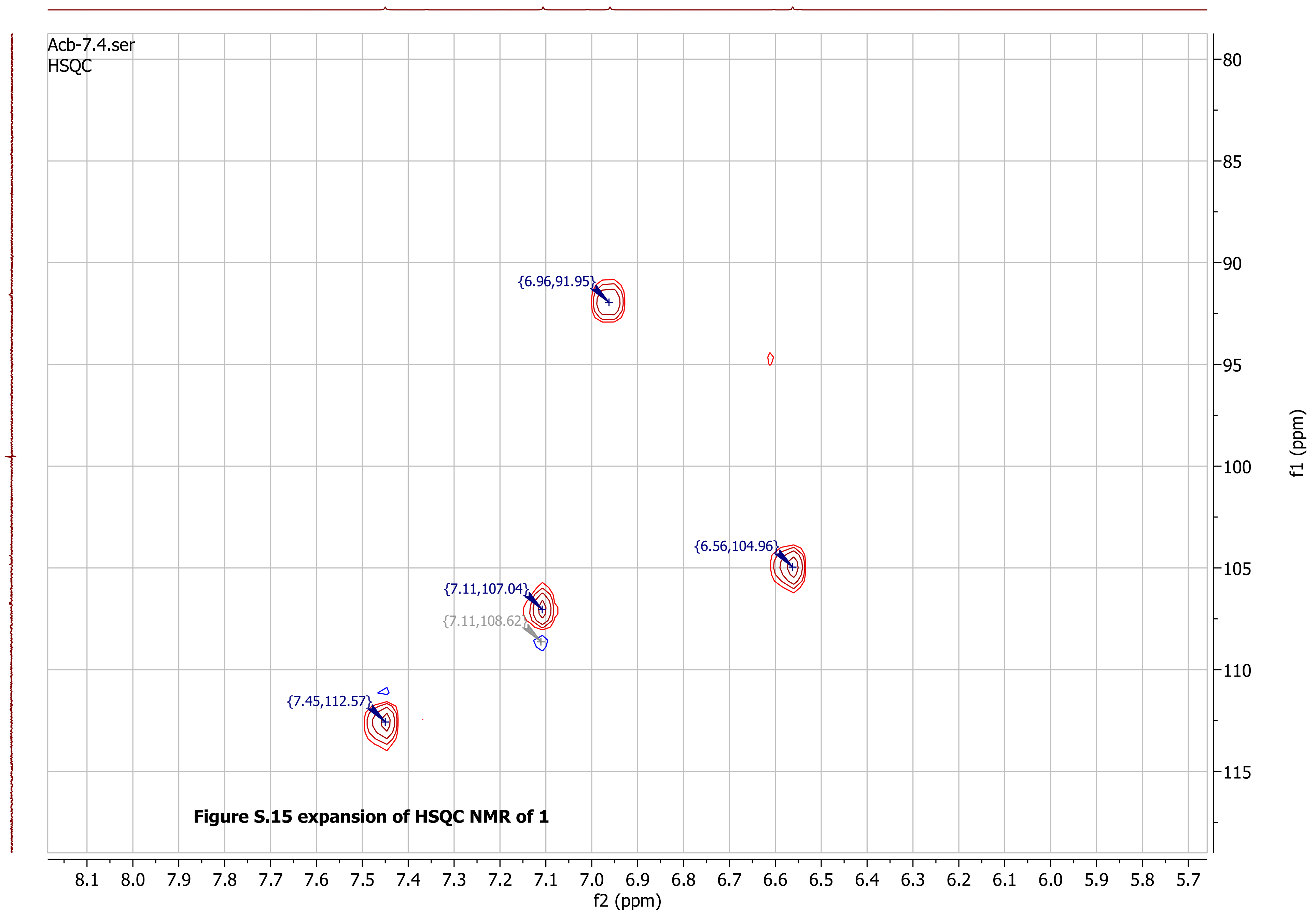


**Figure S.15 expansion of HSQC NMR of 1**

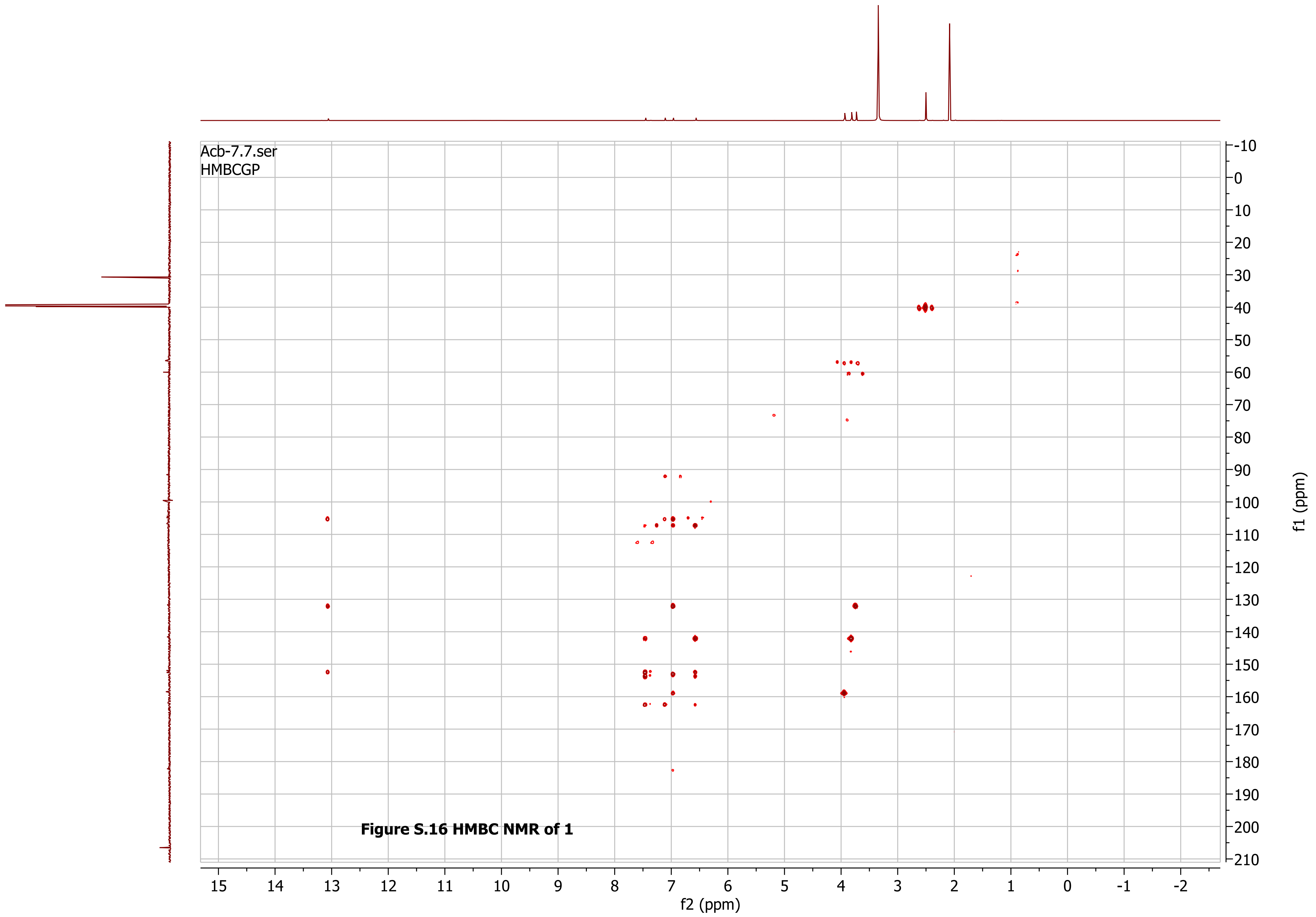


**Figure S.16 HMBC NMR of 1**

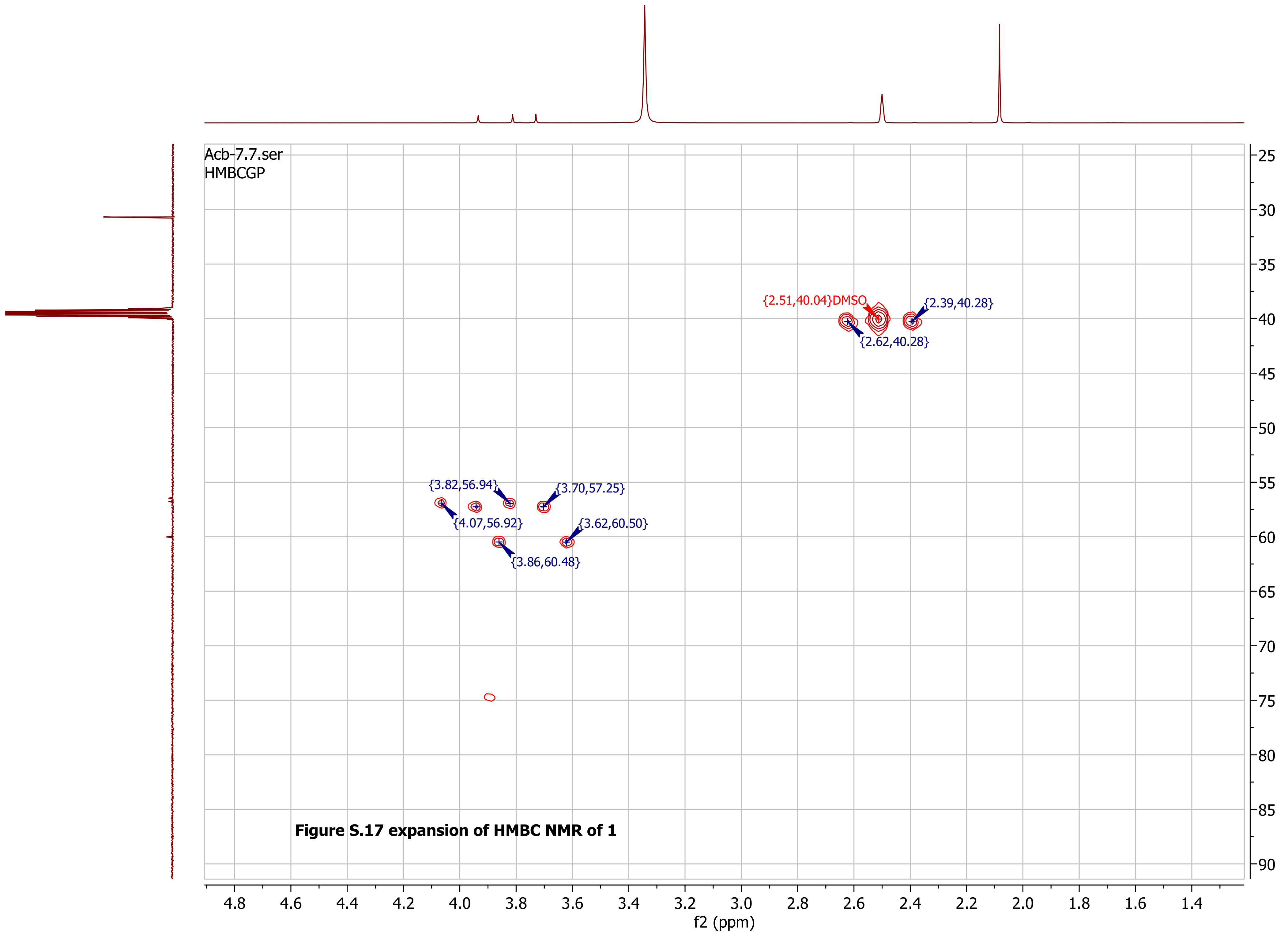


**Figure S.17 expansion of HMBC NMR of 1**

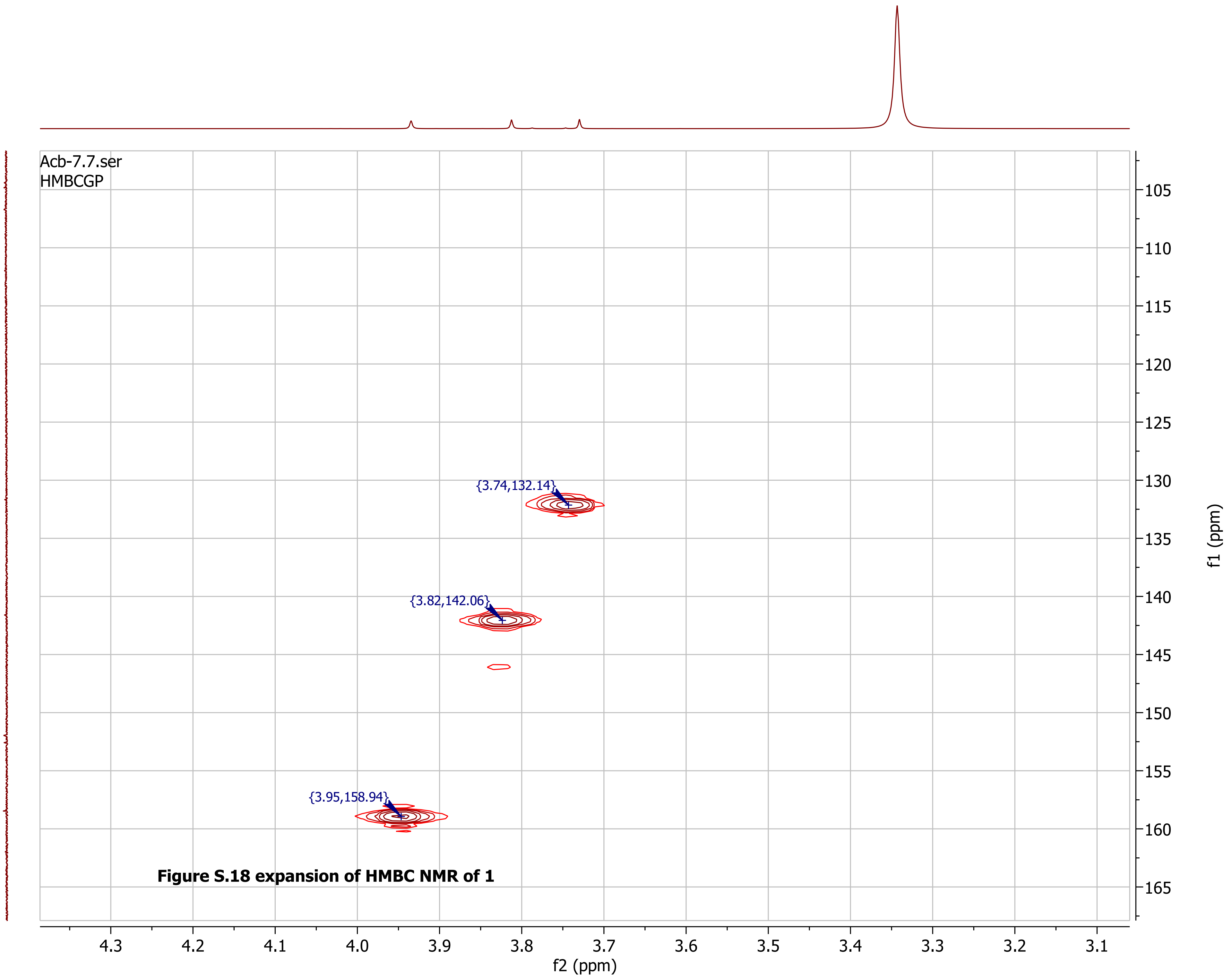


**Figure S.18 expansion of HMBC NMR of 1**

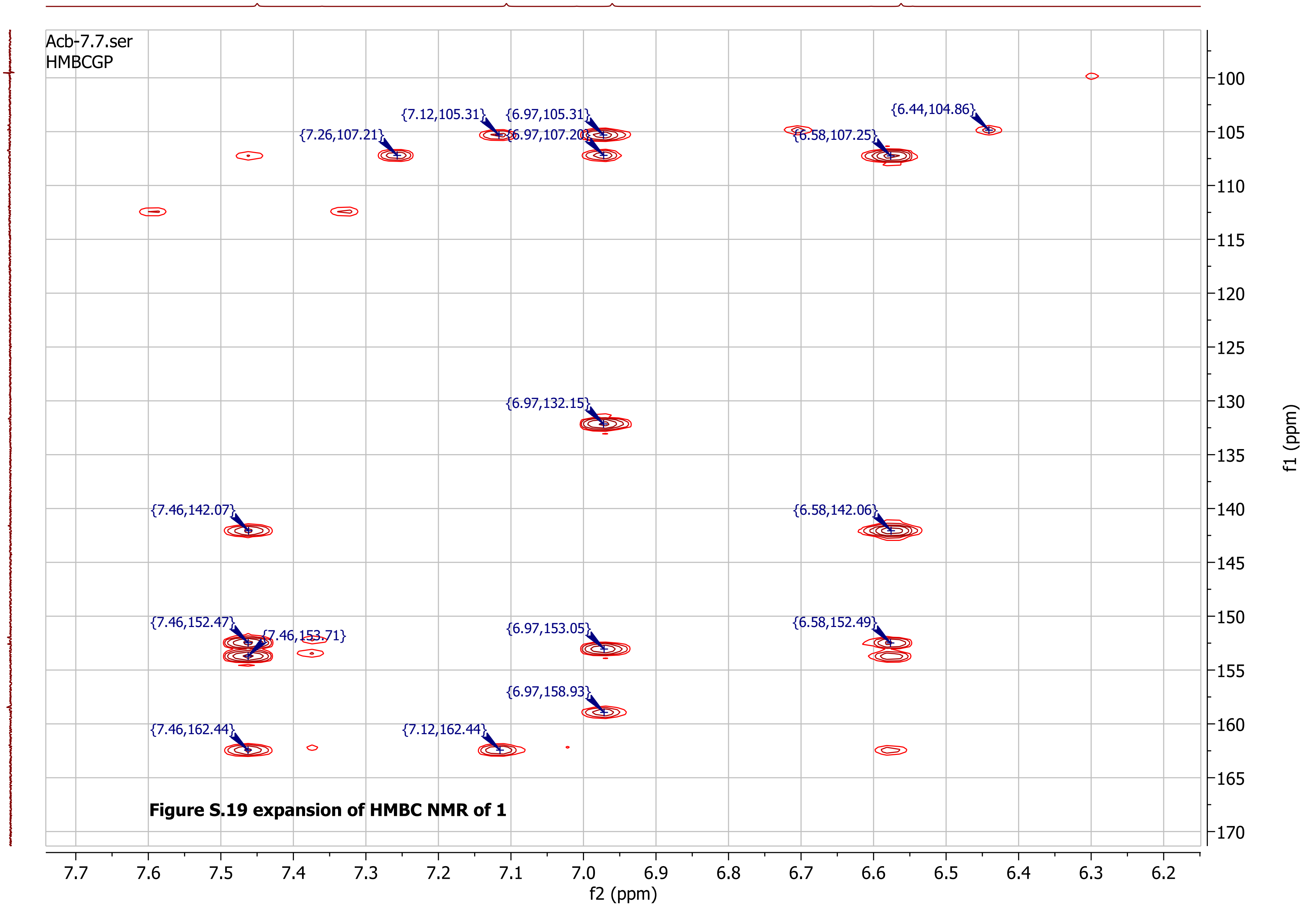


**Figure S.19 expansion of HMBC NMR of 1**

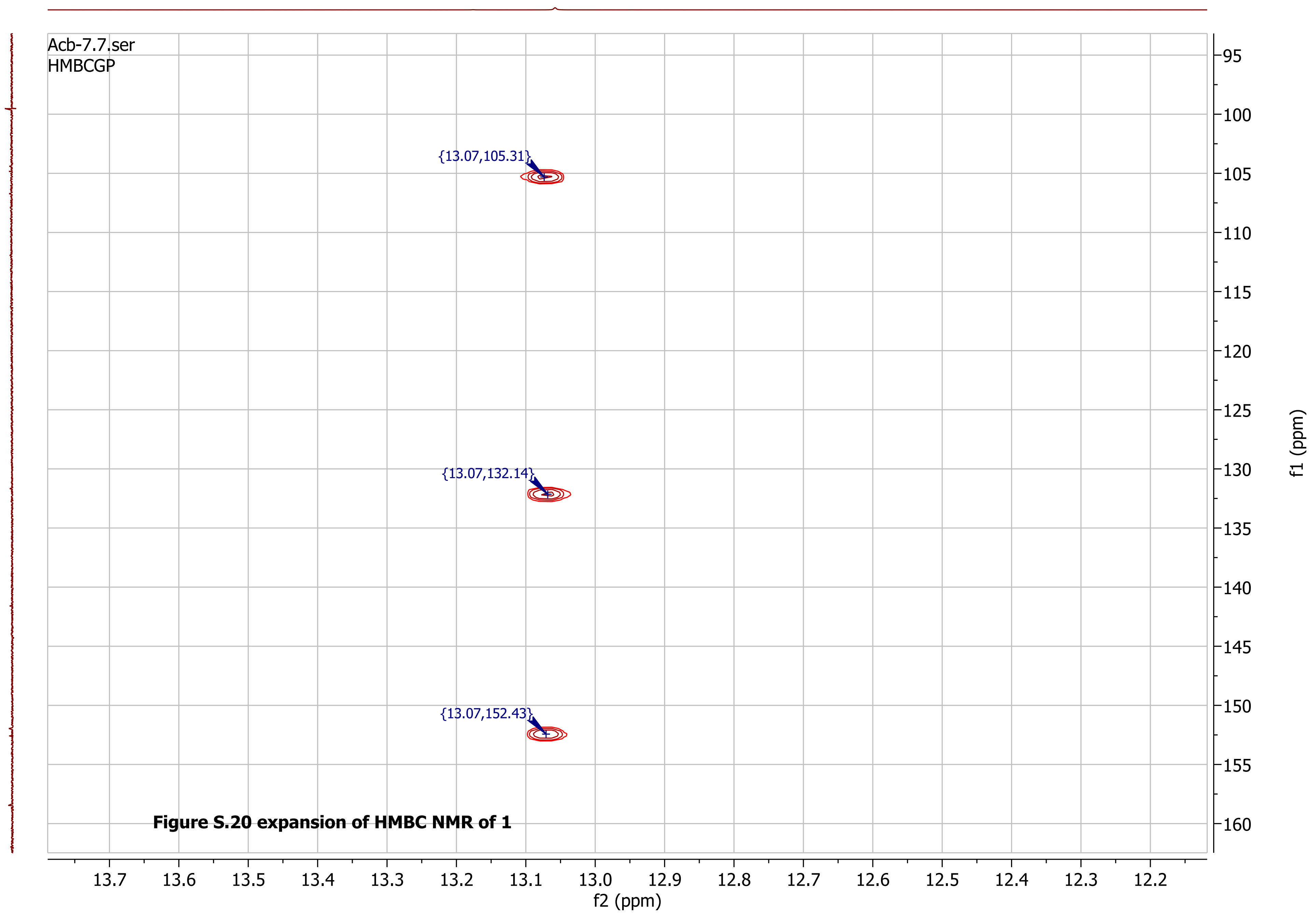


**Figure S.20 expansion of HMBC NMR of 1**

# Toxicity Report

# Flavonoid-1

# TOPKAT_Developmental_Toxicity_Potential

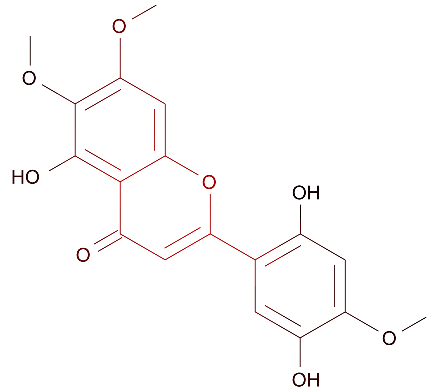

$C_{18}H_{16}O_8$
Molecular Weight: 360.31483
ALogP: 2.361
Rotatable Bonds: 4
Acceptors: 8
Donors: 3

## Model Prediction

Prediction: Toxic
Probability: 0.802
Enrichment: 1.52
Bayesian Score: 6.74
Mahalanobis Distance: 11.1
Mahalanobis Distance p-value: 0.000872

Prediction: Positive if the Bayesian score is above the estimated best cutoff value from minimizing the false positive and false negative rate.
Probability: The esimated probability that the sample is in the positive category. This assumes that the Bayesian score follows a normal distribution and is different from the prediction using a cutoff.
Enrichment: An estimate of enrichment, that is, the increased likelihood (versus random) of this sample being in the category.
Bayesian Score: The standard Laplacian-modified Bayesian score.
Mahalanobis Distance: The Mahalanobis distance (MD) is the distance to the center of the training data. The larger the MD, the less trustworthy the prediction.
Mahalanobis Distance p-value: The p-value gives the fraction of training data with an MD greater than or equal to the one for the given sample, assuming normally distributed data. The smaller the p-value, the less trustworthy the prediciton. For highly non-normal X properties (e.g., fingerprints), the MD p-value is wildly inaccurate.

## Structural Similar Compounds

| Name | Ochratoxin a | D&C Yellow 8 | N-(3-Piperidyl)3;4;5-trimethoxybenzamide |
|---|---|---|---|
| Structure | 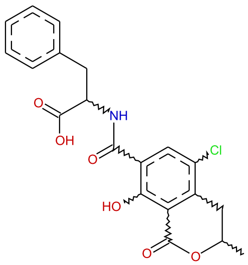 | 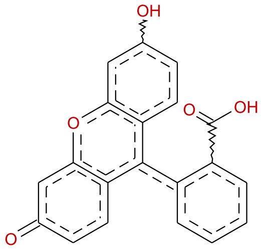 | 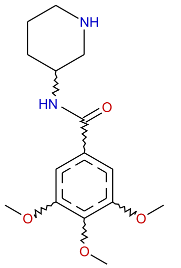 |
| Actual Endpoint | Toxic | Non-Toxic | Toxic |
| Predicted Endpoint | Toxic | Non-Toxic | Toxic |
| Distance | 0.630 | 0.708 | 0.719 |
| Reference | Toxicol Appl Pharmacol 37(2):331-8; 1976 | Food Chem Toxicol 24:819-823; 1986 | Kiso to Rinsho 18:91-101; 1984 |

## Model Applicability

Unknown features are fingerprint features in the query molecule, but not found or appearing too infrequently in the training set.

1. All properties and OPS components are within expected ranges.

## Feature Contribution

**Top features for positive contribution**

| Fingerprint | Bit/Smiles | Feature Structure | Score | Toxic in training set |
|---|---|---|---|---|
| SCFP_6 | 2116304939 | 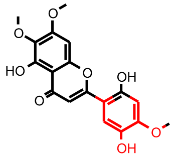 [*]O[c]1:[cH]:[*]:[c]([*]):[cH]:[c]:1O | 0.504 | 5 out of 5 |

| | | | | |
|---|---|---|---|---|
| SCFP_6 | 616636418 | 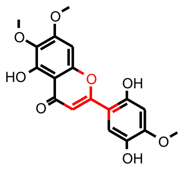<br>[*]O\C(=C/[*])\[c](:[ *]):[*] | 0.478 | 4 out of 4 |
| SCFP_6 | 591469355 | 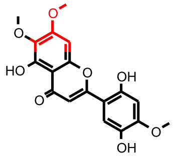<br>[*][c](:[*]):[c](OC):[cH]:[*] | 0.411 | 10 out of 12 |
| **Top Features for negative contribution** | | | | |
| **Fingerprint** | **Bit/Smiles** | **Feature Structure** | **Score** | **Toxic in training set** |
| SCFP_6 | -496409612 | 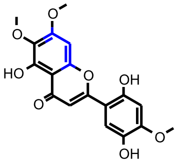<br>[*][c](:[*]):[cH]:[c]([*]):[*] | 0 | 82 out of 163 |
| SCFP_6 | 0 | 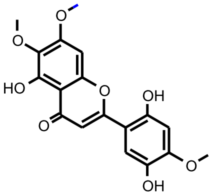<br>[*]C | 0 | 122 out of 230 |
| SCFP_6 | 13 | 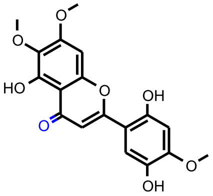<br>[*]=O | 0 | 81 out of 162 |

# Simeprevir

# TOPKAT_Developmental_Toxicity_Potential

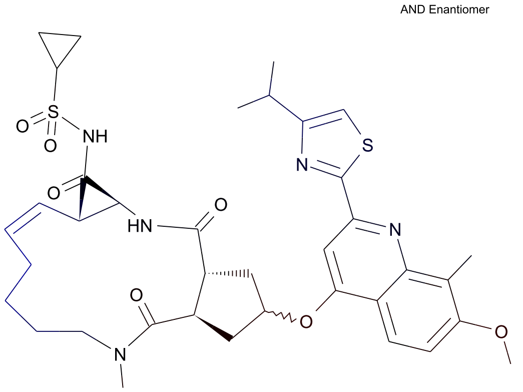


$C_{38}H_{47}N_5O_7S_2$
Molecular Weight: 749.93908
ALogP: 4.796
Rotatable Bonds: 8
Acceptors: 9
Donors: 2

## Structural Similar Compounds

| Name | Reserpate | Bromocriptine | Estramustine Phosphate Disodium (Free acid form) |
|---|---|---|---|
| Structure | 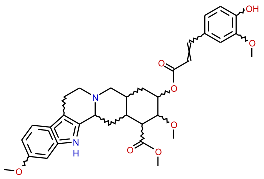 | 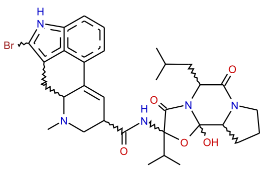 | 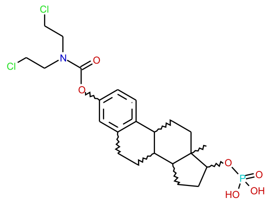 |
| Actual Endpoint | Toxic | Non-Toxic | Non-Toxic |
| Predicted Endpoint | Toxic | Non-Toxic | Non-Toxic |
| Distance | 0.675 | 0.778 | 0.819 |
| Reference | Oyo Yakuri 18:105-124; 1979 | Toxicol Lett 50:189-194; 1990 | Oyo Yakuri 20(6):1219-1236; 1980 |

## Model Prediction

Prediction: Non-Toxic
Probability: 0.438
Enrichment: 0.833
Bayesian Score: -3.14
Mahalanobis Distance: 13.4
Mahalanobis Distance p-value: 9.2e-008

Prediction: Positive if the Bayesian score is above the estimated best cutoff value from minimizing the false positive and false negative rate.
Probability: The esimated probability that the sample is in the positive category. This assumes that the Bayesian score follows a normal distribution and is different from the prediction using a cutoff.
Enrichment: An estimate of enrichment, that is, the increased likelihood (versus random) of this sample being in the category.
Bayesian Score: The standard Laplacian-modified Bayesian score.
Mahalanobis Distance: The Mahalanobis distance (MD) is the distance to the center of the training data. The larger the MD, the less trustworthy the prediction.
Mahalanobis Distance p-value: The p-value gives the fraction of training data with an MD greater than or equal to the one for the given sample, assuming normally distributed data. The smaller the p-value, the less trustworthy the prediciton. For highly non-normal X properties (e.g., fingerprints), the MD p-value is wildly inaccurate.

## Model Applicability

Unknown features are fingerprint features in the query molecule, but not found or appearing too infrequently in the training set.

1. OPS PC12 out of range. Value: 3.5244. Training min, max, SD, explained variance: -3.7514, 3.3159, 1.318, 0.0255.

## Feature Contribution

| Top features for positive contribution | | | | |
|---|---|---|---|---|
| Fingerprint | Bit/Smiles | Feature Structure | Score | Toxic in training set |
| SCFP_6 | 591469355 | 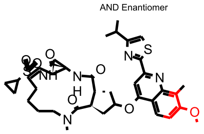 [*][c](:[*]):[c](OC):[cH]:[*] | 0.411 | 10 out of 12 |

| | | | | |
|---|---|---|---|---|
| SCFP_6 | -395254381 | 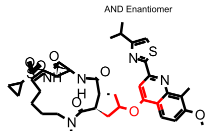  [*]:[cH]:[c](OC1C[*][ *]C1):[c](:[*]):[*] | 0.271 | 1 out of 1 |
| SCFP_6 | -109092631 | 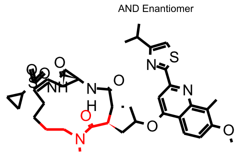  [*]CCN(C)C(=O)C([*])[ *] | 0.271 | 1 out of 1 |

## Top Features for negative contribution

| Fingerprint | Bit/Smiles | Feature Structure | Score | Toxic in training set |
|---|---|---|---|---|
| SCFP_6 | -98332825 | 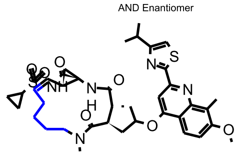  [*]CCCCC=[*] | -0.718 | 0 out of 2 |
| SCFP_6 | -1476112164 | 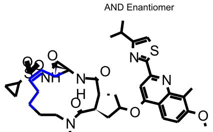  [*]CC\C=C/C1[*][*]1 | -0.718 | 0 out of 2 |
| SCFP_6 | 1260369147 | 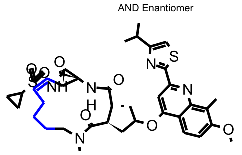  [*]CCC\C=C/[*] | -0.718 | 0 out of 2 |

# Flavonoid-1

# TOPKAT_Mouse_Female_FDA_None_vs_Carcinogen

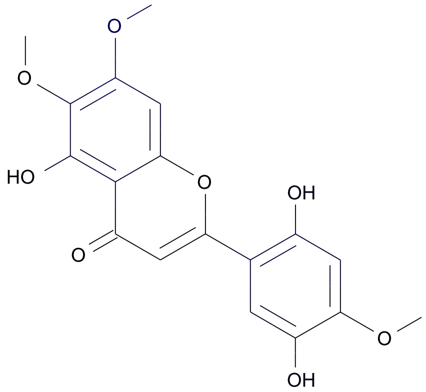

$C_{18}H_{16}O_8$
Molecular Weight: 360.31483
ALogP: 2.361
Rotatable Bonds: 4
Acceptors: 8
Donors: 3

## Structural Similar Compounds

| Name | Nedocromil | Sulfasalazine | Budesonide |
|---|---|---|---|
| Structure | 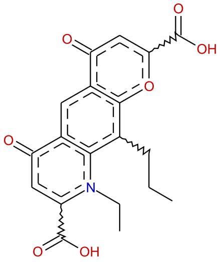 | 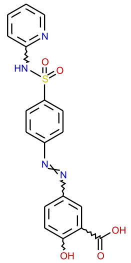 | 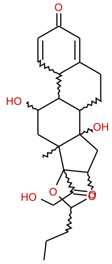 |
| Actual Endpoint | Non-Carcinogen | Carcinogen | Non-Carcinogen |
| Predicted Endpoint | Non-Carcinogen | Carcinogen | Non-Carcinogen |
| Distance | 0.600 | 0.669 | 0.686 |
| Reference | US FDA (Centre for Drug Eval.& Res./Off. Testing & Res.) Sept. 1997 | US FDA (Centre for Drug Eval.& Res./Off. Testing & Res.) Sept. 1997 | US FDA (Centre for Drug Eval.& Res./Off. Testing & Res.) Sept. 1997 |

## Model Prediction

Prediction: Non-Carcinogen
Probability: 0.208
Enrichment: 0.648
Bayesian Score: -4.85
Mahalanobis Distance: 11.7
Mahalanobis Distance p-value: 0.0252

Prediction: Positive if the Bayesian score is above the estimated best cutoff value from minimizing the false positive and false negative rate.
Probability: The esimated probability that the sample is in the positive category. This assumes that the Bayesian score follows a normal distribution and is different from the prediction using a cutoff.
Enrichment: An estimate of enrichment, that is, the increased likelihood (versus random) of this sample being in the category.
Bayesian Score: The standard Laplacian-modified Bayesian score.
Mahalanobis Distance: The Mahalanobis distance (MD) is the distance to the center of the training data. The larger the MD, the less trustworthy the prediction.
Mahalanobis Distance p-value: The p-value gives the fraction of training data with an MD greater than or equal to the one for the given sample, assuming normally distributed data. The smaller the p-value, the less trustworthy the prediciton. For highly non-normal X properties (e.g., fingerprints), the MD p-value is wildly inaccurate.

## Model Applicability

Unknown features are fingerprint features in the query molecule, but not found or appearing too infrequently in the training set.

1. All properties and OPS components are within expected ranges.
2. Unknown ECFP_2 feature: 1573564860: [*]O\C(=C/[*])\[c](:[*]):[*]

## Feature Contribution

**Top features for positive contribution**

| Fingerprint | Bit/Smiles | Feature Structure | Score | Carcinogen in training set |
|---|---|---|---|---|
| ECFP_6 | 464808839 | 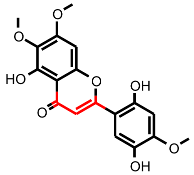 [*]C(=CC(=[*])[*])[*] | 0.524 | 8 out of 14 |

| | | | | |
|---|---|---|---|---|
| ECFP_6 | 2106656448 | 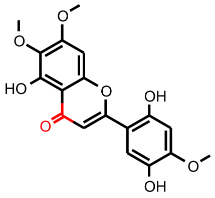 [*]C(=O)[*] | 0.254 | 31 out of 77 |
| ECFP_6 | -560785749 | 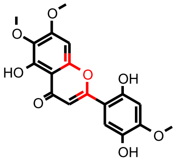 [*]C(=[*])O[c](:[*]):[*] | 0.212 | 1 out of 2 |
| **Top Features for negative contribution** | | | | |
| **Fingerprint** | **Bit/Smiles** | **Feature Structure** | **Score** | **Carcinogen in training set** |
| ECFP_6 | 1307307440 | 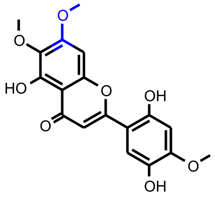 [*]:[c](:[*])OC | -0.558 | 4 out of 25 |
| ECFP_6 | 1334400011 | 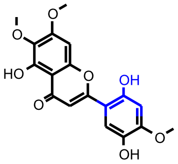 [*][c](:[*]):[c](O):[cH]:[*] | -0.496 | 3 out of 18 |
| ECFP_6 | -427397688 | 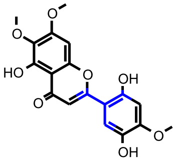 [*]C(=[*])[c](:[cH]:[*]):[c]([*]):[*] | -0.476 | 5 out of 28 |

# Simeprevir

# TOPKAT_Mouse_Female_FDA_None_vs_Carcinogen

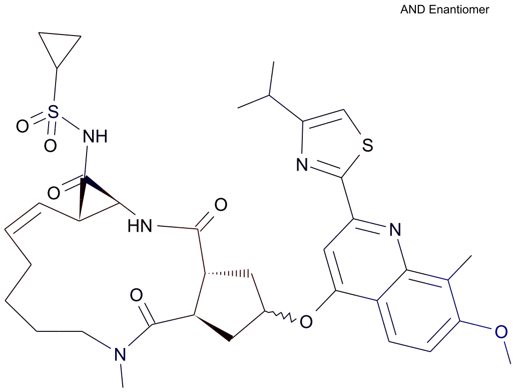


$C_{38}H_{47}N_5O_7S_2$

Molecular Weight: 749.93908

ALogP: 4.796

Rotatable Bonds: 8

Acceptors: 9

Donors: 2

## Structural Similar Compounds

| Name | Reserpine | Bromocriptine | Itraconazole |
|---|---|---|---|
| Structure | 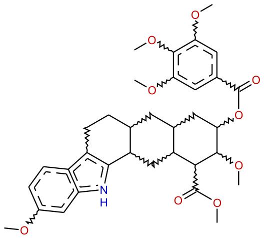 | 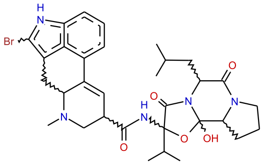 | 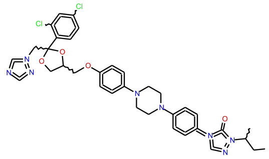 |
| Actual Endpoint | Carcinogen | Non-Carcinogen | Non-Carcinogen |
| Predicted Endpoint | Carcinogen | Non-Carcinogen | Non-Carcinogen |
| Distance | 0.783 | 0.847 | 0.870 |
| Reference | US FDA (Centre for Drug Eval.& Res./Off. Testing & Res.) Sept. 1997 | US FDA (Centre for Drug Eval.& Res./Off. Testing & Res.) Sept. 1997 | US FDA (Centre for Drug Eval.& Res./Off. Testing & Res.) Sept. 1997 |

## Model Prediction

Prediction: Non-Carcinogen

Probability: 0.207

Enrichment: 0.646

Bayesian Score: -4.97

Mahalanobis Distance: 15.4

Mahalanobis Distance p-value: 4.64e-008

Prediction: Positive if the Bayesian score is above the estimated best cutoff value from minimizing the false positive and false negative rate.
Probability: The esimated probability that the sample is in the positive category. This assumes that the Bayesian score follows a normal distribution and is different from the prediction using a cutoff.
Enrichment: An estimate of enrichment, that is, the increased likelihood (versus random) of this sample being in the category.
Bayesian Score: The standard Laplacian-modified Bayesian score.
Mahalanobis Distance: The Mahalanobis distance (MD) is the distance to the center of the training data. The larger the MD, the less trustworthy the prediction.
Mahalanobis Distance p-value: The p-value gives the fraction of training data with an MD greater than or equal to the one for the given sample, assuming normally distributed data. The smaller the p-value, the less trustworthy the prediciton. For highly non-normal X properties (e.g., fingerprints), the MD p-value is wildly inaccurate.

## Model Applicability

Unknown features are fingerprint features in the query molecule, but not found or appearing too infrequently in the training set.

1. OPS PC4 out of range. Value: 5.1697. Training min, max, SD, explained variance: -4.7116, 4.7287, 2.103, 0.0450.
2. OPS PC14 out of range. Value: -3.7168. Training min, max, SD, explained variance: -3.5274, 4.3994, 1.457, 0.0216.
3. OPS PC17 out of range. Value: 3.3515. Training min, max, SD, explained variance: -4.7306, 3.3103, 1.364, 0.0189.
4. Unknown ECFP_2 feature: -309676563: [*]N[C@]1(C[C@H]1[*])C(=[*])[*]
5. Unknown ECFP_2 feature: -1049290660: [*]C1([*])C[C@H]1C=[*]
6. Unknown ECFP_2 feature: 360408239: [*]C\C=C/[*]
7. Unknown ECFP_2 feature: 1616402542: [*]CN(C)C(=[*])[*]
8. Unknown ECFP_2 feature: -1818486371: [*]NC(=O)C1([*])[*][*]1
9. Unknown ECFP_2 feature: 1310940530: [*]C([*])O[c](:[*]):[*]
10. Unknown ECFP_2 feature: -253227249: [*]:[c](:[*])[c]1:n:[*]:[*]:s:1
11. Unknown ECFP_2 feature: 733491677: [*]:[c](:[*])C(C)C
12. Unknown ECFP_2 feature: 2121941848: [*]NS(=O)(=O)C1[*][*]1
13. Unknown ECFP_2 feature: -622223421: [*]S(=[*])(=[*])C1CC1

## Feature Contribution

**Top features for positive contribution**

| Fingerprint | Bit/Smiles | Feature Structure | Score | Carcinogen in training set |
|---|---|---|---|---|
| ECFP_6 | -2095963820 | 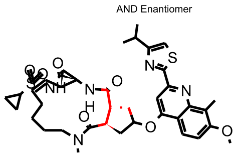 [*][C@@H]1[*][*]C[C@H]1C(=[*])[*] | 0.891 | 12 out of 14 |
| ECFP_6 | 2082767335 | 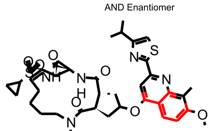 [*][c](:[*]):[c]1:[cH]:[cH]:[c]([*]):[*]:[c]:1:[*] | 0.617 | 2 out of 2 |
| ECFP_6 | -1331088410 | 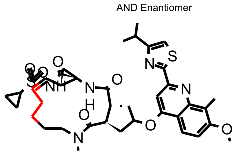 [*]CCC=[*] | 0.442 | 2 out of 3 |
| **Top Features for negative contribution** | | | | |
| **Fingerprint** | **Bit/Smiles** | **Feature Structure** | **Score** | **Carcinogen in training set** |
| ECFP_6 | 2077607946 | 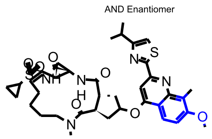 [*]O[c]1:[cH]:[cH]:[c](:[*]):[*]:[c]:1[*] | -1.15 | 0 out of 7 |

| | | | | |
|---|---|---|---|---|
| ECFP_6 | 1526862590 | 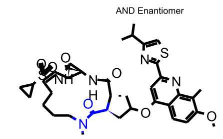  [*]C([*])C(=O)N([*])[*] | -0.638 | 1 out of 9 |
| ECFP_6 | 1307307440 | 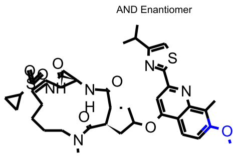  [*]:[c](:[*])OC | -0.558 | 4 out of 25 |

# Flavonoid-1

# TOPKAT_Mouse_Male_FDA_None_vs_Carcinogen

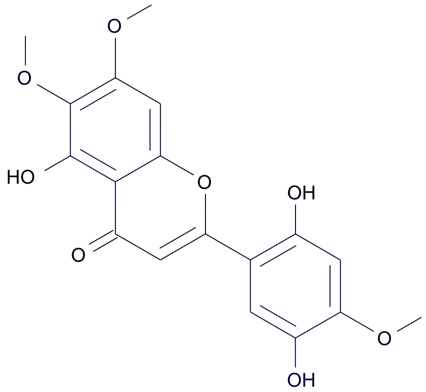

$C_{18}H_{16}O_{8}$
Molecular Weight: 360.31483
ALogP: 2.361
Rotatable Bonds: 4
Acceptors: 8
Donors: 3

## Structural Similar Compounds

| Name | Nedocromil | Sulfasalazine | Olsalazine |
|---|---|---|---|
| Structure | 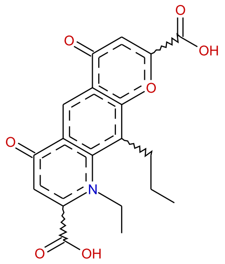 | 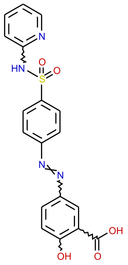 | 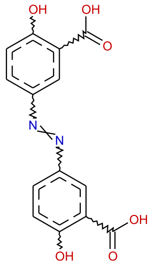 |
| Actual Endpoint | Non-Carcinogen | Carcinogen | Non-Carcinogen |
| Predicted Endpoint | Non-Carcinogen | Carcinogen | Carcinogen |
| Distance | 0.586 | 0.641 | 0.691 |
| Reference | US FDA (Centre for Drug Eval.& Res./Off. Testing & Res.) Sept. 1997 | US FDA (Centre for Drug Eval.& Res./Off. Testing & Res.) Sept. 1997 | US FDA (Centre for Drug Eval.& Res./Off. Testing & Res.) Sept. 1997 |

## Model Prediction

Prediction: Non-Carcinogen
Probability: 0.217
Enrichment: 0.737
Bayesian Score: -3.97
Mahalanobis Distance: 12
Mahalanobis Distance p-value: 0.00673

Prediction: Positive if the Bayesian score is above the estimated best cutoff value from minimizing the false positive and false negative rate.
Probability: The esimated probability that the sample is in the positive category. This assumes that the Bayesian score follows a normal distribution and is different from the prediction using a cutoff.
Enrichment: An estimate of enrichment, that is, the increased likelihood (versus random) of this sample being in the category.
Bayesian Score: The standard Laplacian-modified Bayesian score.
Mahalanobis Distance: The Mahalanobis distance (MD) is the distance to the center of the training data. The larger the MD, the less trustworthy the prediction.
Mahalanobis Distance p-value: The p-value gives the fraction of training data with an MD greater than or equal to the one for the given sample, assuming normally distributed data. The smaller the p-value, the less trustworthy the prediciton. For highly non-normal X properties (e.g., fingerprints), the MD p-value is wildly inaccurate.

## Model Applicability

Unknown features are fingerprint features in the query molecule, but not found or appearing too infrequently in the training set.

1. All properties and OPS components are within expected ranges.
2. Unknown FCFP_2 feature: -1678245750: [*]O\C(=C/[*])\[c](:[*]):[*]

## Feature Contribution

**Top features for positive contribution**

| Fingerprint | Bit/Smiles | Feature Structure | Score | Carcinogen in training set |
|---|---|---|---|---|
| FCFP_6 | 451847724 | 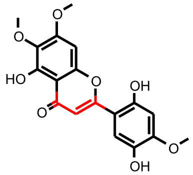 [*]C(=CC(=[*])[*])[*] | 0.479 | 21 out of 48 |

| | | | | |
|---|---|---|---|---|
| FCFP_6 | 1679744180 | 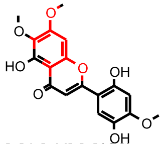<br>[*]O[c]1:[cH]:[c](O[*<br>]):[c]([*]):[*]:[c]:<br>1[*] | 0.271 | 1 out of 2 |
| FCFP_6 | 1872154524 | 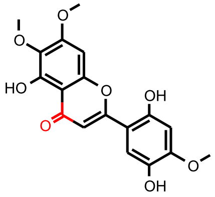<br>[*]C(=O)[*] | 0.205 | 69 out of 213 |
| **Top Features for negative contribution** | | | | |
| **Fingerprint** | **Bit/Smiles** | **Feature Structure** | **Score** | **Carcinogen in training set** |
| FCFP_6 | 1028934530 | 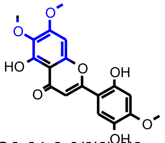<br>[*]O[c]1:[c]([*]):[*]<br>:[c]([*]):[cH]:[c]:1<br>OC | -0.596 | 1 out of 10 |
| FCFP_6 | -1549192822 | 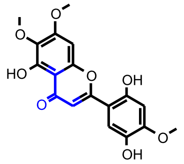<br>[*]=CC(=O)[c](:[*]):[<br>*] | -0.489 | 3 out of 21 |
| FCFP_6 | -1604301295 | 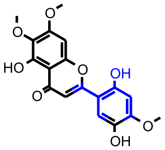<br>[*]C(=[*])[c]1:[cH]:[<br>*]:[c]([*]):[cH]:[c]<br>:1O | -0.445 | 2 out of 14 |

# Simeprevir

# TOPKAT_Mouse_Male_FDA_None_vs_Carcinogen

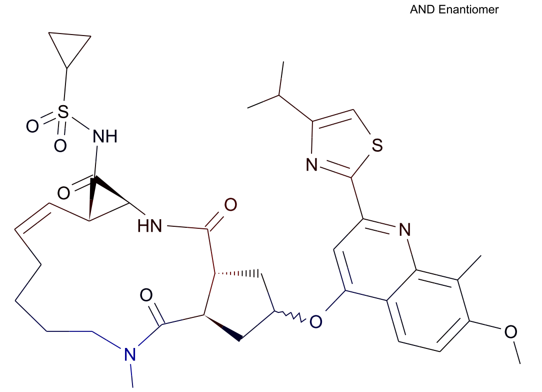


$C_{38}H_{47}N_5O_7S_2$
Molecular Weight: 749.93908
ALogP: 4.796
Rotatable Bonds: 8
Acceptors: 9
Donors: 2

## Model Prediction

Prediction: Non-Carcinogen
Probability: 0.258
Enrichment: 0.877
Bayesian Score: -1.92
Mahalanobis Distance: 18.3
Mahalanobis Distance p-value: 6.22e-015

Prediction: Positive if the Bayesian score is above the estimated best cutoff value from minimizing the false positive and false negative rate.
Probability: The esimated probability that the sample is in the positive category. This assumes that the Bayesian score follows a normal distribution and is different from the prediction using a cutoff.
Enrichment: An estimate of enrichment, that is, the increased likelihood (versus random) of this sample being in the category.
Bayesian Score: The standard Laplacian-modified Bayesian score.
Mahalanobis Distance: The Mahalanobis distance (MD) is the distance to the center of the training data. The larger the MD, the less trustworthy the prediction.
Mahalanobis Distance p-value: The p-value gives the fraction of training data with an MD greater than or equal to the one for the given sample, assuming normally distributed data. The smaller the p-value, the less trustworthy the prediciton. For highly non-normal X properties (e.g., fingerprints), the MD p-value is wildly inaccurate.

## Structural Similar Compounds

| Name | Reserpine | Bromocriptine | Itraconazole |
|---|---|---|---|
| Structure | 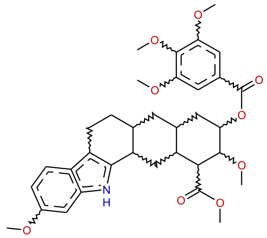 | 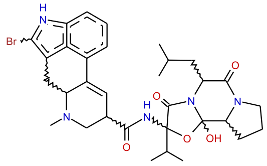 | 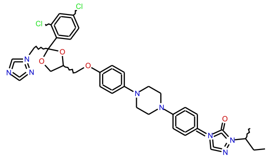 |
| Actual Endpoint | Carcinogen | Non-Carcinogen | Non-Carcinogen |
| Predicted Endpoint | Carcinogen | Non-Carcinogen | Non-Carcinogen |
| Distance | 0.775 | 0.832 | 0.865 |
| Reference | US FDA (Centre for Drug Eval.& Res./Off. Testing & Res.) Sept. 1997 | US FDA (Centre for Drug Eval.& Res./Off. Testing & Res.) Sept. 1997 | US FDA (Centre for Drug Eval.& Res./Off. Testing & Res.) Sept. 1997 |

## Model Applicability

Unknown features are fingerprint features in the query molecule, but not found or appearing too infrequently in the training set.

1. All properties and OPS components are within expected ranges.
2. Unknown FCFP_2 feature: 690481386: [*]:[c](:[*])[c]1:n:[*]:[*]:s:1

## Feature Contribution

**Top features for positive contribution**

| Fingerprint | Bit/Smiles | Feature Structure | Score | Carcinogen in training set |
|---|---|---|---|---|
| FCFP_6 | -55265897 | 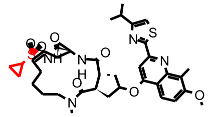 AND Enantiomer [*]S(=[*])(=[*])C1CC1 | 0.594 | 17 out of 34 |

| | | | | |
|---|---|---|---|---|
| FCFP_6 | -1289661876 | 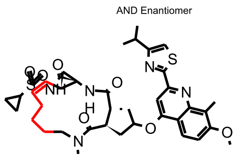 [*]CCC\C=C/[*] | 0.517 | 2 out of 3 |
| FCFP_6 | 451847724 | 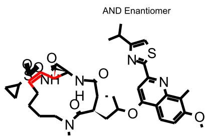 [*]C(=CC(=[*])[*])[*] | 0.479 | 21 out of 48 |
| **Top Features for negative contribution** | | | | |
| **Fingerprint** | **Bit/Smiles** | **Feature Structure** | **Score** | **Carcinogen in training set** |
| FCFP_6 | -98332825 | 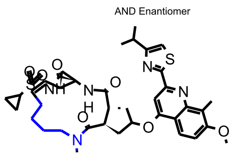 [*]CCCCN([*])[*] | -0.793 | 1 out of 13 |
| FCFP_6 | -1972798083 | 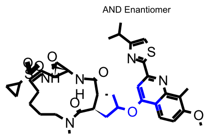 [*]:[cH]:[c](OC1C[*][ *]C1):[c](:[*]):[*] | -0.582 | 0 out of 3 |
| FCFP_6 | -1553874037 | 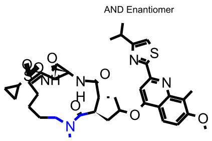 [*]CN(C)C(=[*])[*] | -0.45 | 5 out of 32 |

# Flavonoid-1 TOPKAT_Ocular_Irritancy_Mild_vs_Moderate_Severe

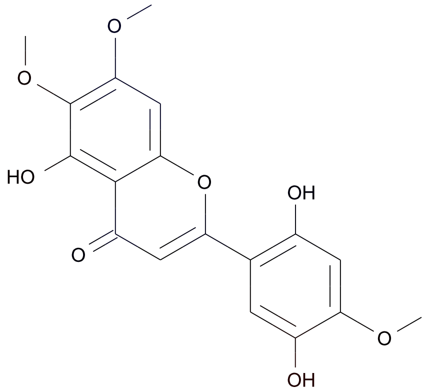


$C_{18}H_{16}O_8$
Molecular Weight: 360.31483
ALogP: 2.361
Rotatable Bonds: 4
Acceptors: 8
Donors: 3

## Model Prediction

Prediction: Mild
Probability: 0.804
Enrichment: 1.17
Bayesian Score: -0.831
Mahalanobis Distance: 8.69
Mahalanobis Distance p-value: 0.674

Prediction: Positive if the Bayesian score is above the estimated best cutoff value from minimizing the false positive and false negative rate.
Probability: The esimated probability that the sample is in the positive category. This assumes that the Bayesian score follows a normal distribution and is different from the prediction using a cutoff.
Enrichment: An estimate of enrichment, that is, the increased likelihood (versus random) of this sample being in the category.
Bayesian Score: The standard Laplacian-modified Bayesian score.
Mahalanobis Distance: The Mahalanobis distance (MD) is the distance to the center of the training data. The larger the MD, the less trustworthy the prediction.
Mahalanobis Distance p-value: The p-value gives the fraction of training data with an MD greater than or equal to the one for the given sample, assuming normally distributed data. The smaller the p-value, the less trustworthy the prediciton. For highly non-normal X properties (e.g., fingerprints), the MD p-value is wildly inaccurate.

## Structural Similar Compounds

| Name | ANTHRAQUINONE; 1;5-DIAMINO-4;8-DIHYDROXY-3-(p-METHOXYPHENYL)- | 4;4'-DIAMINO-1;1'-DIANTHRIMIDE | ANTHRAQUINONE; 1-((2-HYDROXYETHYL)AMINO)-4-(METHYLAMINO)- |
|---|---|---|---|
| Structure | 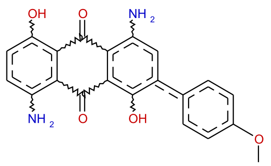 | 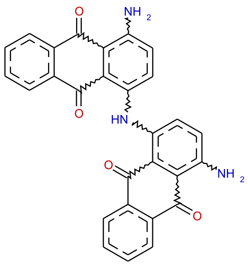 | 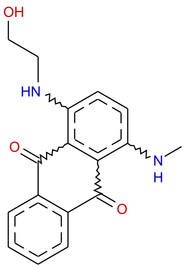 |
| Actual Endpoint | Mild | Mild | Mild |
| Predicted Endpoint | Mild | Mild | Mild |
| Distance | 0.709 | 0.726 | 0.787 |
| Reference | 28ZPAK 245;72 | 28ZPAK-;125;72 | 28ZPAK 245;72 |

## Model Applicability

Unknown features are fingerprint features in the query molecule, but not found or appearing too infrequently in the training set.

1. All properties and OPS components are within expected ranges.
2. Unknown FCFP_2 feature: -1678245750: [*]O\C(=C/[*])\[c](:[*]):[*]

## Feature Contribution

| Top features for positive contribution | | | | |
|---|---|---|---|---|
| Fingerprint | Bit/Smiles | Feature Structure | Score | Moderate_Severe in training set |
| FCFP_10 | 1679744180 | 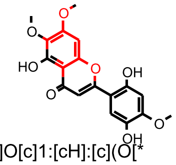 [*]O[c]1:[cH]:[c](O[*]):[c]([*]):[*]:[c]:1[*] | 0.256 | 2 out of 2 |

| | | | | |
|---|---|---|---|---|
| FCFP_10 | 1028934530 | 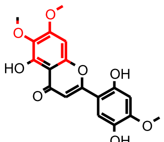 [*]O[c]1:[c]([*]):[*]:[c]([*]):[cH]:[c]:1OC | 0.256 | 2 out of 2 |
| FCFP_10 | 7 | 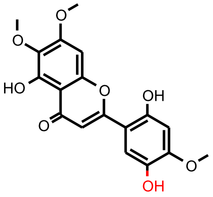 [*]O | 0.219 | 117 out of 142 |
| **Top Features for negative contribution** | | | | |
| **Fingerprint** | **Bit/Smiles** | **Feature Structure** | **Score** | **Moderate_Severe in training set** |
| FCFP_10 | -1977641857 | 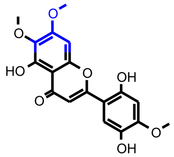 [*][c](:[*]):[c](OC):[cH]:[*] | -0.78 | 4 out of 15 |
| FCFP_10 | -1099193755 | 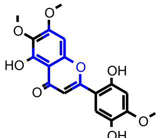 [*]C1=[*]C(=[*])[c]2:[c]([*]):[*]:[c]([*]):[cH]:[c]:2O1 | -0.361 | 2 out of 5 |
| FCFP_10 | 136627117 | 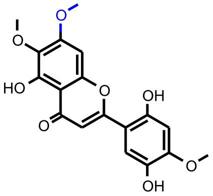 [*]OC | -0.316 | 46 out of 96 |

# Simeprevir

# TOPKAT_Ocular_Irritancy_Mild_vs_Moderate_Severe

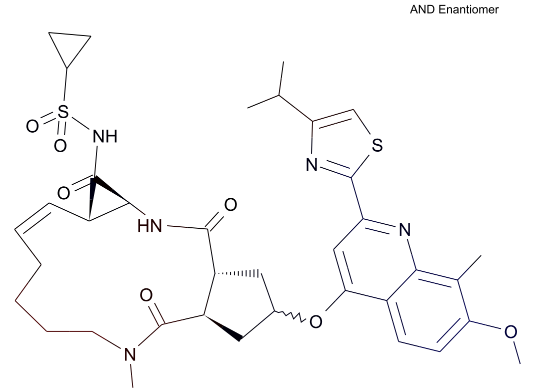


$C_{38}H_{47}N_5O_7S_2$

Molecular Weight: 749.93908

ALogP: 4.796

Rotatable Bonds: 8

Acceptors: 9

Donors: 2

## Structural Similar Compounds

| Name | Anthraquinone; 1;1'-(anthraquinon-1;5-ylenediimino)di- | Anthraquinone; 1;1'-(anthraquinon-1;4-ylenediimino)di- | 4;4'-DIAMINO-1;1'-DIANTHRIMIDE |
|---|---|---|---|
| Structure | 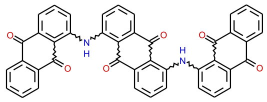 | 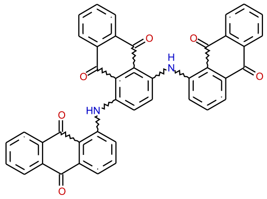 | 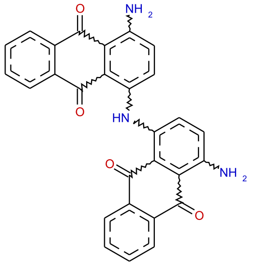 |
| Actual Endpoint | Mild | Mild | Mild |
| Predicted Endpoint | Mild | Mild | Mild |
| Distance | 0.875 | 0.875 | 1.102 |
| Reference | Prehled Prumyslove Toxikologie; Organicke Latky; Marhold; J. pp 736;86 | Prehled Prumyslove Toxikologie; Organicke Latky; Marhold; J. pp 736;86 | 28ZPAK-;125;72 |

## Model Prediction

Prediction: Mild

Probability: 0.777

Enrichment: 1.13

Bayesian Score: -1.77

Mahalanobis Distance: 17.1

Mahalanobis Distance p-value: 9.62e-022

Prediction: Positive if the Bayesian score is above the estimated best cutoff value from minimizing the false positive and false negative rate.
Probability: The esimated probability that the sample is in the positive category. This assumes that the Bayesian score follows a normal distribution and is different from the prediction using a cutoff.
Enrichment: An estimate of enrichment, that is, the increased likelihood (versus random) of this sample being in the category.
Bayesian Score: The standard Laplacian-modified Bayesian score.
Mahalanobis Distance: The Mahalanobis distance (MD) is the distance to the center of the training data. The larger the MD, the less trustworthy the prediction.
Mahalanobis Distance p-value: The p-value gives the fraction of training data with an MD greater than or equal to the one for the given sample, assuming normally distributed data. The smaller the p-value, the less trustworthy the prediciton. For highly non-normal X properties (e.g., fingerprints), the MD p-value is wildly inaccurate.

## Model Applicability

Unknown features are fingerprint features in the query molecule, but not found or appearing too infrequently in the training set.

1. OPS PC15 out of range. Value: 5.5683. Training min, max, SD, explained variance: -4.4073, 5.1625, 1.138, 0.0158.

## Feature Contribution

### Top features for positive contribution

| Fingerprint | Bit/Smiles | Feature Structure | Score | Moderate_Severe in training set |
|---|---|---|---|---|
| FCFP_10 | -2015019763 | 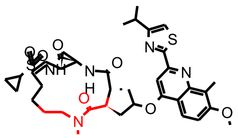 AND Enantiomer [*]CCN(C)C(=O)C([*])[*] | 0.294 | 3 out of 3 |

| | | | | |
|---|---|---|---|---|
| FCFP_10 | -1474971978 | 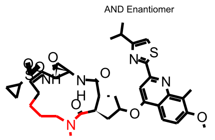  [*]CCCN(C)C(=[*])[*] | 0.259 | 14 out of 16 |
| FCFP_10 | -124655670 | 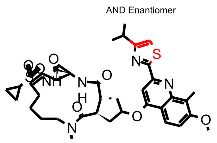  [*][c]1:[*]:[*]:s:[cH]:1 | 0.259 | 14 out of 16 |
| **Top Features for negative contribution** | | | | |
| **Fingerprint** | **Bit/Smiles** | **Feature Structure** | **Score** | **Moderate_Severe in training set** |
| FCFP_10 | -1977641857 | 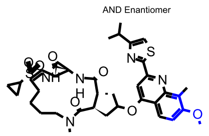  [*][c](:[*]):[c](OC):[cH]:[*] | -0.78 | 4 out of 15 |
| FCFP_10 | -528918648 | 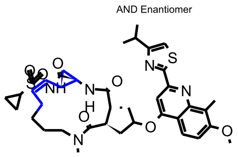  [*]C\C=C/[C@@H]1CC1([*])[*] | -0.651 | 4 out of 13 |
| FCFP_10 | 690481386 | 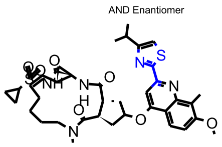  [*]:[c](:[*])[c]1:n:[*]:[*]:s:1 | -0.6 | 1 out of 4 |

# Flavonoid-1 TOPKAT_Ocular_Irritancy_None_vs_Irritant

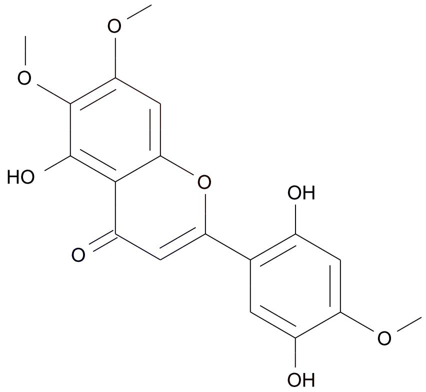


$C_{18}H_{16}O_8$

Molecular Weight: 360.31483

ALogP: 2.361

Rotatable Bonds: 4

Acceptors: 8

Donors: 3

## Structural Similar Compounds

| **Name** | **Disperse Black 9** | **4;4'-DIAMINO-1;1'-DIANTHRIMIDE** | **ANTHRAQUINONE; 1;5-DIAMINO-4;8-DIHYDROXY-3-(p-METHOXYPHENYL)-** |
|---|---|---|---|
| Structure | 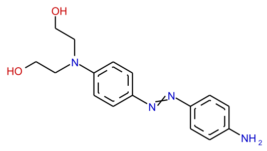 | 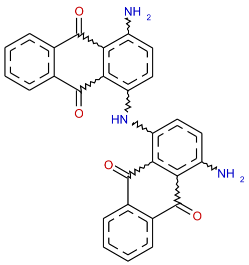 | 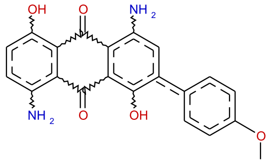 |
| Actual Endpoint | Non-Irritant | Irritant | Irritant |
| Predicted Endpoint | Non-Irritant | Irritant | Irritant |
| Distance | 0.704 | 0.708 | 0.711 |
| Reference | J. Am. Coll. Toxicol. 5(3):205;1986 | 28ZPAK-;125;72 | 28ZPAK 245;72 |

## Model Prediction

Prediction: Irritant

Probability: 0.976

Enrichment: 1.15

Bayesian Score: 0.256

Mahalanobis Distance: 8.37

Mahalanobis Distance p-value: 0.817

Prediction: Positive if the Bayesian score is above the estimated best cutoff value from minimizing the false positive and false negative rate.
Probability: The esimated probability that the sample is in the positive category. This assumes that the Bayesian score follows a normal distribution and is different from the prediction using a cutoff.
Enrichment: An estimate of enrichment, that is, the increased likelihood (versus random) of this sample being in the category.
Bayesian Score: The standard Laplacian-modified Bayesian score.
Mahalanobis Distance: The Mahalanobis distance (MD) is the distance to the center of the training data. The larger the MD, the less trustworthy the prediction.
Mahalanobis Distance p-value: The p-value gives the fraction of training data with an MD greater than or equal to the one for the given sample, assuming normally distributed data. The smaller the p-value, the less trustworthy the prediciton. For highly non-normal X properties (e.g., fingerprints), the MD p-value is wildly inaccurate.

## Model Applicability

Unknown features are fingerprint features in the query molecule, but not found or appearing too infrequently in the training set.

1. All properties and OPS components are within expected ranges.
2. Unknown FCFP_2 feature: -1678245750: [*]O\C(=C/[*])\[c](:[*]):[*]

## Feature Contribution

**Top features for positive contribution**

| **Fingerprint** | **Bit/Smiles** | **Feature Structure** | **Score** | **Irritant in training set** |
|---|---|---|---|---|
| FCFP_12 | -1099193755 | 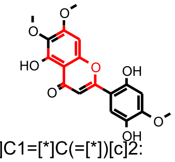 [*]C1=[*]C(=[*])[c]2:[c]([*]):[*]:[c]([*]):[cH]:[c]:2O1 | 0.175 | 5 out of 5 |

| | | | | |
|---|---|---|---|---|
| FCFP_12 | 1679744180 | 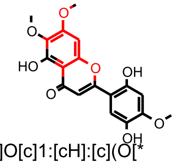<br>[*]O[c]1:[cH]:[c](O[*]):[c]([*]):[*]:[c]:1[*] | 0.137 | 2 out of 2 |
| FCFP_12 | 1028934530 | 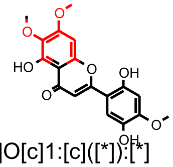<br>[*]O[c]1:[c]([*]):[*]:[c]([*]):[cH]:[c]:1OC | 0.137 | 2 out of 2 |
| **Top Features for negative contribution** | | | | |
| **Fingerprint** | **Bit/Smiles** | **Feature Structure** | **Score** | **Irritant in training set** |
| FCFP_12 | 1244036906 | 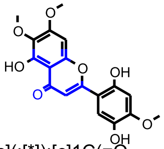<br>[*][c](:[*]):[c]1C(=O)C=C([*])[*][c]:1:[*] | -0.592 | 0 out of 1 |
| FCFP_12 | 1673930087 | 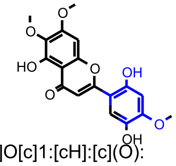<br>[*]O[c]1:[cH]:[c](O):[c]([*]):[*]:[c]:1[*] | -0.218 | 5 out of 8 |
| FCFP_12 | 136627117 | 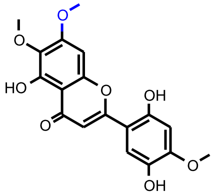<br>[*]OC | 0 | 96 out of 113 |

# Simeprevir

# TOPKAT_Ocular_Irritancy_None_vs_Irritant

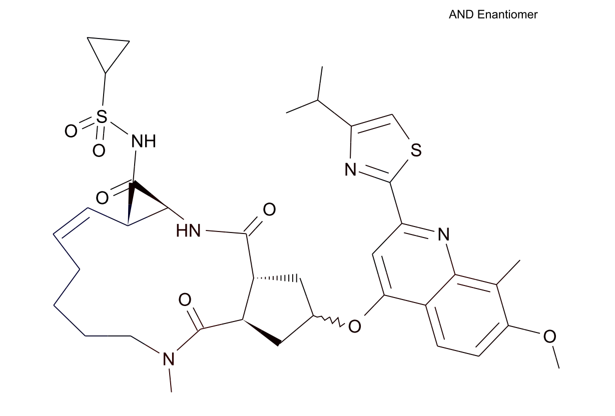


$C_{38}H_{47}N_5O_7S_2$
Molecular Weight: 749.93908
ALogP: 4.796
Rotatable Bonds: 8
Acceptors: 9
Donors: 2

## Structural Similar Compounds

| Name | Anthraquinone; 1;1'-(anthraquinon-1;5-ylenediimino)di- | Anthraquinone; 1;1'-(anthraquinon-1;4-ylenediimino)di- | ANILINE;N;N'-1;4-ANTHRAQUINONYLENEBIS(4-PHENOXY- |
|---|---|---|---|
| Structure | 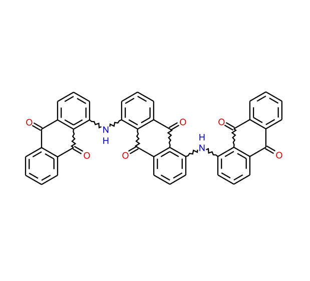 | 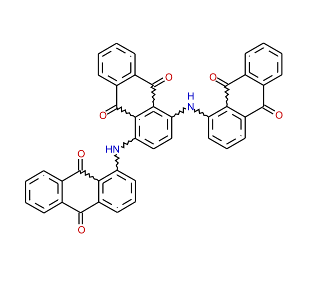 | 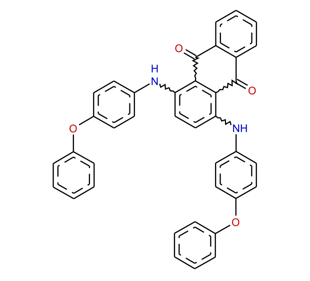 |
| Actual Endpoint | Irritant | Irritant | Non-Irritant |
| Predicted Endpoint | Irritant | Irritant | Non-Irritant |
| Distance | 0.837 | 0.838 | 1.053 |
| Reference | Prehled Prumyslove Toxikologie; Organicke Latky; Marhold; J. pp 736;86 | Prehled Prumyslove Toxikologie; Organicke Latky; Marhold; J. pp 736;86 | 28ZPAK-;114;72 |

## Model Prediction

Prediction: Irritant
Probability: 1
Enrichment: 1.18
Bayesian Score: 1.81
Mahalanobis Distance: 16.5
Mahalanobis Distance p-value: 1.59e-019

Prediction: Positive if the Bayesian score is above the estimated best cutoff value from minimizing the false positive and false negative rate.
Probability: The esimated probability that the sample is in the positive category. This assumes that the Bayesian score follows a normal distribution and is different from the prediction using a cutoff.
Enrichment: An estimate of enrichment, that is, the increased likelihood (versus random) of this sample being in the category.
Bayesian Score: The standard Laplacian-modified Bayesian score.
Mahalanobis Distance: The Mahalanobis distance (MD) is the distance to the center of the training data. The larger the MD, the less trustworthy the prediction.
Mahalanobis Distance p-value: The p-value gives the fraction of training data with an MD greater than or equal to the one for the given sample, assuming normally distributed data. The smaller the p-value, the less trustworthy the prediciton. For highly non-normal X properties (e.g., fingerprints), the MD p-value is wildly inaccurate.

## Model Applicability

Unknown features are fingerprint features in the query molecule, but not found or appearing too infrequently in the training set.

1. All properties and OPS components are within expected ranges.

## Feature Contribution

**Top features for positive contribution**

| Fingerprint | Bit/Smiles | Feature Structure | Score | Irritant in training set |
|---|---|---|---|---|
| FCFP_12 | 1747237384 | 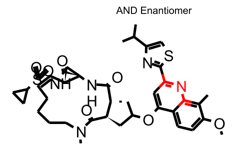 [*][c](:[*]):n:[c](:[*]):[*] | 0.208 | 44 out of 44 |

| | | | | |
|---|---|---|---|---|
| FCFP_12 | 178336375 | 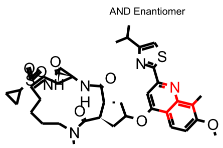 [*][c](:[*]):[c](:n:[ *]):[c](:[*]):[*] | 0.202 | 19 out of 19 |
| FCFP_12 | -124655670 | 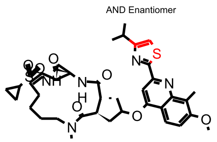 [*][c]1:[*]:[*]:s:[cH ]:1 | 0.2 | 16 out of 16 |
| **Top Features for negative contribution** | | | | |
| **Fingerprint** | **Bit/Smiles** | **Feature Structure** | **Score** | **Irritant in training set** |
| FCFP_12 | -1812846456 | 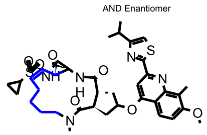 [*]CCCC\C=C/C1[*][*]1 | -1.01 | 2 out of 9 |
| FCFP_12 | 690511177 | 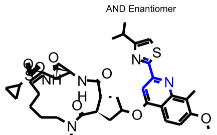 [*]:[cH]:[c](:n:[*])[ c](:[*]):[*] | -0.268 | 1 out of 2 |
| FCFP_12 | 1175638033 | 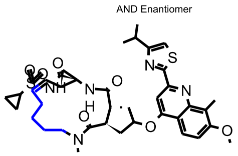 [*]CCCCC=[*] | -0.133 | 207 out of 293 |

# Flavonoid-1

# TOPKAT_Rat_Female_FDA_None_vs_Carcinogen

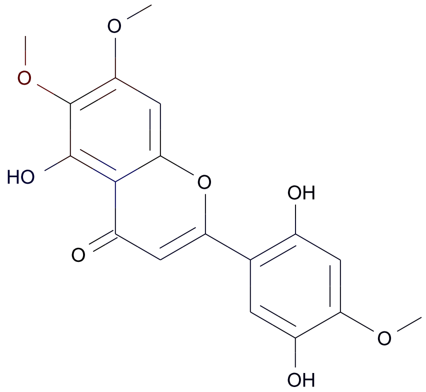


$C_{18}H_{16}O_8$
Molecular Weight: 360.31483
ALogP: 2.361
Rotatable Bonds: 4
Acceptors: 8
Donors: 3

## Structural Similar Compounds

| Name | Nedocromil | Sulfasalazine | Budesonide |
|---|---|---|---|
| Structure | 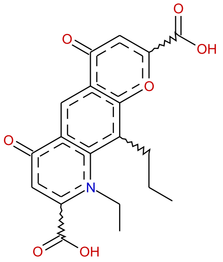 | 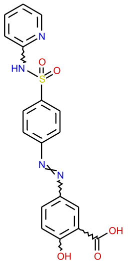 | 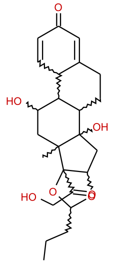 |
| Actual Endpoint | Non-Carcinogen | Non-Carcinogen | Non-Carcinogen |
| Predicted Endpoint | Non-Carcinogen | Non-Carcinogen | Non-Carcinogen |
| Distance | 0.619 | 0.681 | 0.700 |
| Reference | US FDA (Centre for Drug Eval.& Res./Off. Testing & Res.) Sept. 1997 | US FDA (Centre for Drug Eval.& Res./Off. Testing & Res.) Sept. 1997 | US FDA (Centre for Drug Eval.& Res./Off. Testing & Res.) Sept. 1997 |

## Model Prediction

Prediction: Non-Carcinogen
Probability: 0.248
Enrichment: 0.771
Bayesian Score: -2.89
Mahalanobis Distance: 10.5
Mahalanobis Distance p-value: 0.181

Prediction: Positive if the Bayesian score is above the estimated best cutoff value from minimizing the false positive and false negative rate.
Probability: The esimated probability that the sample is in the positive category. This assumes that the Bayesian score follows a normal distribution and is different from the prediction using a cutoff.
Enrichment: An estimate of enrichment, that is, the increased likelihood (versus random) of this sample being in the category.
Bayesian Score: The standard Laplacian-modified Bayesian score.
Mahalanobis Distance: The Mahalanobis distance (MD) is the distance to the center of the training data. The larger the MD, the less trustworthy the prediction.
Mahalanobis Distance p-value: The p-value gives the fraction of training data with an MD greater than or equal to the one for the given sample, assuming normally distributed data. The smaller the p-value, the less trustworthy the prediciton. For highly non-normal X properties (e.g., fingerprints), the MD p-value is wildly inaccurate.

## Model Applicability

Unknown features are fingerprint features in the query molecule, but not found or appearing too infrequently in the training set.

1. All properties and OPS components are within expected ranges.

## Feature Contribution

**Top features for positive contribution**

| Fingerprint | Bit/Smiles | Feature Structure | Score | Carcinogen in training set |
|---|---|---|---|---|
| ECFP_12 | 2052151141 | 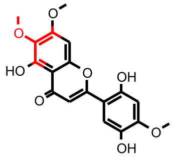 [*][c](:[*]):[c](OC):[c]([*]):[*] | 0.668 | 4 out of 5 |

| | | | | |
|---|---|---|---|---|
| ECFP_12 | 1151345624 | 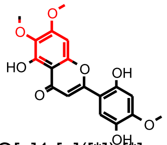 [*]O[c]1:[c]([*]):[*]:[c]([*]):[cH]:[c]:1OC | 0.613 | 2 out of 2 |
| ECFP_12 | -1531301414 | 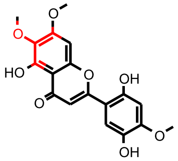 [*]O[c](:[c]([*]):[*]):[c]([*]):[*] | 0.454 | 5 out of 9 |

**Top Features for negative contribution**

| Fingerprint | Bit/Smiles | Feature Structure | Score | Carcinogen in training set |
|---|---|---|---|---|
| ECFP_12 | -1660913849 | 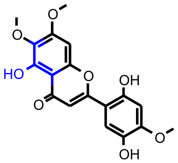 [*][c](:[*]):[c](O):[c]([*]):[*] | -0.941 | 0 out of 5 |
| ECFP_12 | 1408898974 | 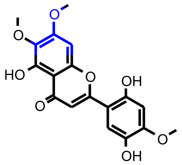 [*]O[c](:[cH]:[*]):[c]([*]):[*] | -0.517 | 5 out of 29 |
| ECFP_12 | 1680623188 | 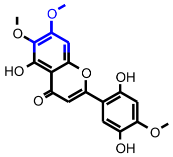 [*][c](:[*]):[c](OC):[cH]:[*] | -0.295 | 3 out of 14 |

# Simeprevir

# TOPKAT_Rat_Female_FDA_None_vs_Carcinogen

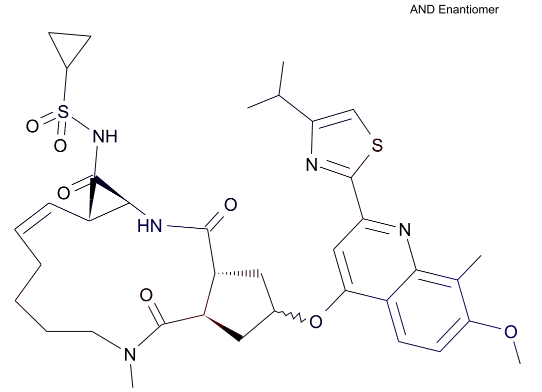


$C_{38}H_{47}N_{5}O_{7}S_{2}$

Molecular Weight: 749.93908

ALogP: 4.796

Rotatable Bonds: 8

Acceptors: 9

Donors: 2

## Model Prediction

Prediction: Non-Carcinogen

Probability: 0.232

Enrichment: 0.719

Bayesian Score: -4.13

Mahalanobis Distance: 14.6

Mahalanobis Distance p-value: 1.61e-007

Prediction: Positive if the Bayesian score is above the estimated best cutoff value from minimizing the false positive and false negative rate.
Probability: The esimated probability that the sample is in the positive category. This assumes that the Bayesian score follows a normal distribution and is different from the prediction using a cutoff.
Enrichment: An estimate of enrichment, that is, the increased likelihood (versus random) of this sample being in the category.
Bayesian Score: The standard Laplacian-modified Bayesian score.
Mahalanobis Distance: The Mahalanobis distance (MD) is the distance to the center of the training data. The larger the MD, the less trustworthy the prediction.
Mahalanobis Distance p-value: The p-value gives the fraction of training data with an MD greater than or equal to the one for the given sample, assuming normally distributed data. The smaller the p-value, the less trustworthy the prediciton. For highly non-normal X properties (e.g., fingerprints), the MD p-value is wildly inaccurate.

## Structural Similar Compounds

| Name | Reserpine | Deserpidine | Bromocriptine |
|---|---|---|---|
| Structure | 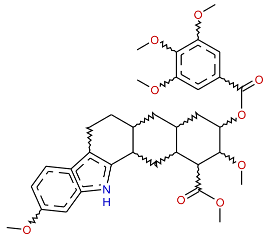 | 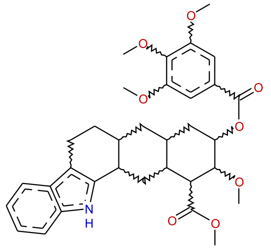 | 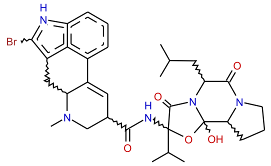 |
| Actual Endpoint | Carcinogen | Carcinogen | Carcinogen |
| Predicted Endpoint | Carcinogen | Carcinogen | Carcinogen |
| Distance | 0.807 | 0.846 | 0.874 |
| Reference | US FDA (Centre for Drug Eval.& Res./Off. Testing & Res.) Sept. 1997 | US FDA (Centre for Drug Eval.& Res./Off. Testing & Res.) Sept. 1997 | US FDA (Centre for Drug Eval.& Res./Off. Testing & Res.) Sept. 1997 |

## Model Applicability

Unknown features are fingerprint features in the query molecule, but not found or appearing too infrequently in the training set.

1. All properties and OPS components are within expected ranges.
2. Unknown ECFP_2 feature: -309676563: [*]N[C@]1(C[C@H]1[*])C(=[*])[*]
3. Unknown ECFP_2 feature: -1049290660: [*]C1([*])C[C@H]1C=[*]
4. Unknown ECFP_2 feature: 1616402542: [*]CN(C)C(=[*])[*]
5. Unknown ECFP_2 feature: -1818486371: [*]NC(=O)C1([*])[*][*]1
6. Unknown ECFP_2 feature: 1310940530: [*]C([*])O[c](:[*]):[*]
7. Unknown ECFP_2 feature: 733491677: [*]:[c](:[*])C(C)C
8. Unknown ECFP_2 feature: 2121941848: [*]NS(=O)(=O)C1[*][*]1
9. Unknown ECFP_2 feature: -622223421: [*]S(=[*])(=[*])C1CC1

## Feature Contribution

**Top features for positive contribution**

| Fingerprint | Bit/Smiles | Feature Structure | Score | Carcinogen in training set |
|---|---|---|---|---|

| | | | | |
|---|---|---|---|---|
| ECFP_12 | -1331088410 | 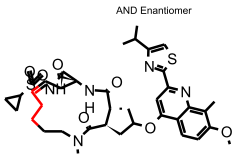 [*]CCC=[*] | 0.725 | 3 out of 3 |
| ECFP_12 | -2095963820 | 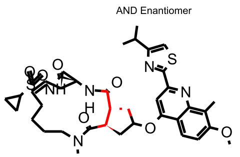 [*][C@@H]1[*][*]C[C@H ]1C(=[*])[*] | 0.722 | 12 out of 17 |
| ECFP_12 | 914325265 | 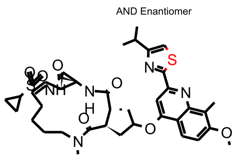 [*]:s:[*] | 0.516 | 8 out of 14 |

## Top Features for negative contribution

| Fingerprint | Bit/Smiles | Feature Structure | Score | Carcinogen in training set |
|---|---|---|---|---|
| ECFP_12 | 2077607946 | 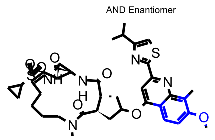 [*]O[c]1:[cH]:[cH]:[c ](:[*]):[*]:[c]:1[*] | -1.25 | 0 out of 8 |
| ECFP_12 | -867777309 | 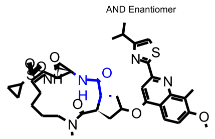 [*]NC(=O)C([*])[*] | -0.661 | 0 out of 3 |

| | | | | |
|---|---|---|---|---|
| ECFP_12 | 1408898974 | 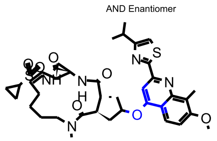 [*]O[c](:[cH]:[*]):[c]([*]):[*] | -0.517 | 5 out of 29 |

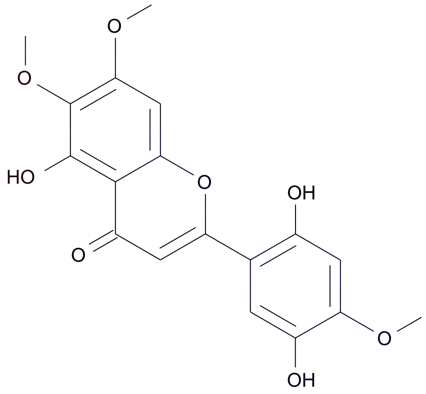

$C_{18}H_{16}O_8$
Molecular Weight: 360.31483
ALogP: 2.361
Rotatable Bonds: 4
Acceptors: 8
Donors: 3

## Model Prediction

Prediction: Non-Carcinogen
Probability: 0.313
Enrichment: 0.938
Bayesian Score: -1.58
Mahalanobis Distance: 11.9
Mahalanobis Distance p-value: 0.0324

Prediction: Positive if the Bayesian score is above the estimated best cutoff value from minimizing the false positive and false negative rate.
Probability: The esimated probability that the sample is in the positive category. This assumes that the Bayesian score follows a normal distribution and is different from the prediction using a cutoff.
Enrichment: An estimate of enrichment, that is, the increased likelihood (versus random) of this sample being in the category.
Bayesian Score: The standard Laplacian-modified Bayesian score.
Mahalanobis Distance: The Mahalanobis distance (MD) is the distance to the center of the training data. The larger the MD, the less trustworthy the prediction.
Mahalanobis Distance p-value: The p-value gives the fraction of training data with an MD greater than or equal to the one for the given sample, assuming normally distributed data. The smaller the p-value, the less trustworthy the prediciton. For highly non-normal X properties (e.g., fingerprints), the MD p-value is wildly inaccurate.

## Structural Similar Compounds

| Name | Nedocromil | Sulfasalazine | Budesonide |
|---|---|---|---|
| Structure | 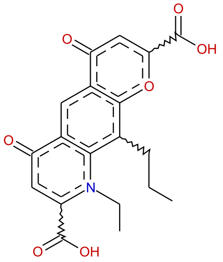 | 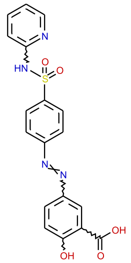 | 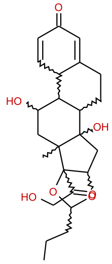 |
| Actual Endpoint | Non-Carcinogen | Carcinogen | Carcinogen |
| Predicted Endpoint | Non-Carcinogen | Carcinogen | Carcinogen |
| Distance | 0.584 | 0.649 | 0.693 |
| Reference | US FDA (Centre for Drug Eval.& Res./Off. Testing & Res.) Sept. 1997 | US FDA (Centre for Drug Eval.& Res./Off. Testing & Res.) Sept. 1997 | US FDA (Centre for Drug Eval.& Res./Off. Testing & Res.) Sept. 1997 |

## Model Applicability

Unknown features are fingerprint features in the query molecule, but not found or appearing too infrequently in the training set.

1. All properties and OPS components are within expected ranges.

## Feature Contribution

**Top features for positive contribution**

| Fingerprint | Bit/Smiles | Feature Structure | Score | Carcinogen in training set |
|---|---|---|---|---|
| SCFP_6 | -1547271378 | 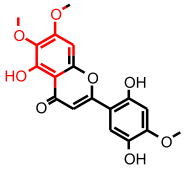 [*]O[c]1:[cH]:[*]:[c]([*]):[c](O):[c]:1OC | 0.603 | 2 out of 2 |

| | | | | |
|---|---|---|---|---|
| SCFP_6 | 392579710 | 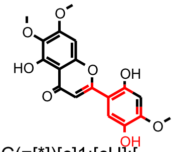 [*]C(=[*])[c]1:[cH]:[ c](O):[c]([*]):[*]:[ c]:1[*] | 0.425 | 2 out of 3 |
| SCFP_6 | -1971196727 | 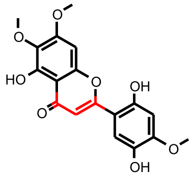 [*]C(=CC(=[*])[*])[*] | 0.361 | 17 out of 36 |

**Top Features for negative contribution**

| Fingerprint | Bit/Smiles | Feature Structure | Score | Carcinogen in training set |
|---|---|---|---|---|
| SCFP_6 | 2116304939 | 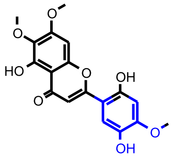 [*]O[c]1:[cH]:[*]:[c] ([*]):[cH]:[c]:1O | -0.825 | 0 out of 4 |
| SCFP_6 | 616636418 | 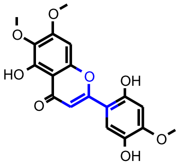 [*]O\C(=C/[*])\[c](:[ *]):[*] | -0.278 | 0 out of 1 |
| SCFP_6 | -617610981 | 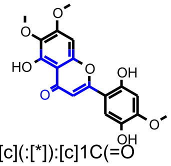 [*][c](:[*]):[c]1C(=O )C=C([*])[*][c]:1:[* ] | -0.278 | 0 out of 1 |

# Simeprevir

# TOPKAT_Rat_Male_FDA_None_vs_Carcinogen

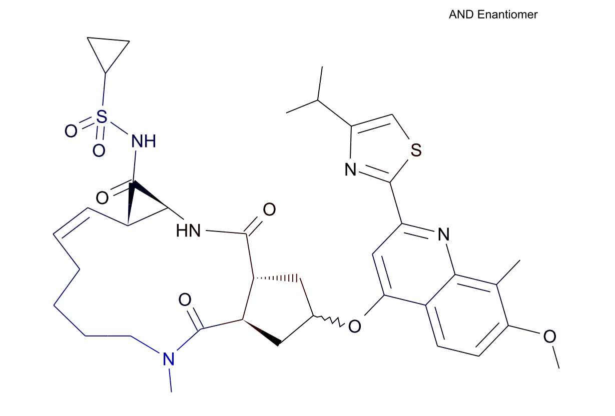


$C_{38}H_{47}N_5O_7S_2$

Molecular Weight: 749.93908

ALogP: 4.796

Rotatable Bonds: 8

Acceptors: 9

Donors: 2

## Structural Similar Compounds

| Name | Reserpine | Deserpidine | Bromocriptine |
|---|---|---|---|
| Structure | 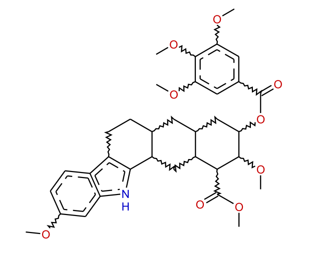 | 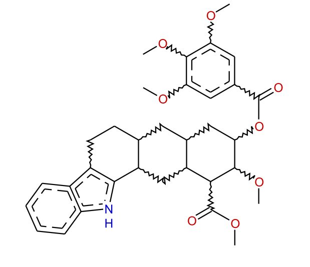 | 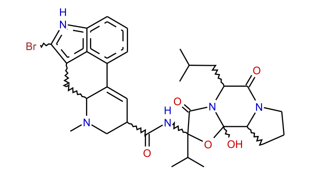 |
| Actual Endpoint | Carcinogen | Carcinogen | Non-Carcinogen |
| Predicted Endpoint | Carcinogen | Carcinogen | Non-Carcinogen |
| Distance | 0.771 | 0.812 | 0.842 |
| Reference | US FDA (Centre for Drug Eval.& Res./Off. Testing & Res.) Sept. 1997 | US FDA (Centre for Drug Eval.& Res./Off. Testing & Res.) Sept. 1997 | US FDA (Centre for Drug Eval.& Res./Off. Testing & Res.) Sept. 1997 |

## Model Prediction

Prediction: Non-Carcinogen

Probability: 0.231

Enrichment: 0.69

Bayesian Score: -5.43

Mahalanobis Distance: 20.3

Mahalanobis Distance p-value: 1.2e-019

Prediction: Positive if the Bayesian score is above the estimated best cutoff value from minimizing the false positive and false negative rate.
Probability: The esimated probability that the sample is in the positive category. This assumes that the Bayesian score follows a normal distribution and is different from the prediction using a cutoff.
Enrichment: An estimate of enrichment, that is, the increased likelihood (versus random) of this sample being in the category.
Bayesian Score: The standard Laplacian-modified Bayesian score.
Mahalanobis Distance: The Mahalanobis distance (MD) is the distance to the center of the training data. The larger the MD, the less trustworthy the prediction.
Mahalanobis Distance p-value: The p-value gives the fraction of training data with an MD greater than or equal to the one for the given sample, assuming normally distributed data. The smaller the p-value, the less trustworthy the prediciton. For highly non-normal X properties (e.g., fingerprints), the MD p-value is wildly inaccurate.

## Model Applicability

Unknown features are fingerprint features in the query molecule, but not found or appearing too infrequently in the training set.

1. OPS PC5 out of range. Value: 7.7766. Training min, max, SD, explained variance: -4.0702, 6.5927, 2.146, 0.0429.
2. OPS PC22 out of range. Value: 3.5288. Training min, max, SD, explained variance: -3.2994, 3.2573, 1.197, 0.0134.

## Feature Contribution

### Top features for positive contribution

| Fingerprint | Bit/Smiles | Feature Structure | Score | Carcinogen in training set |
|---|---|---|---|---|
| SCFP_6 | -1903488337 | 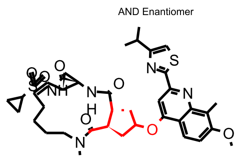 [*]OC1C[C@@H]([*])[C@@H](C1)C(=[*])[*] | 0.603 | 2 out of 2 |

| | | | | |
|---|---|---|---|---|
| SCFP_6 | -1379673609 | 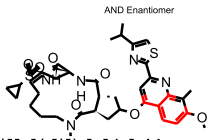<br>[*][c](:[*]):[c]1:[cH<br>]:[cH]:[c]([*]):[*]:<br>[c]:1:[*] | 0.526 | 11 out of 19 |
| SCFP_6 | 1310748454 | 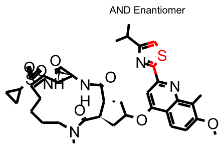<br>[*][c]1:[*]:[*]:[cH]:<br>s:1 | 0.437 | 7 out of 13 |
| **Top Features for negative contribution** | | | | |
| **Fingerprint** | **Bit/Smiles** | **Feature Structure** | **Score** | **Carcinogen in training set** |
| SCFP_6 | 182902497 | 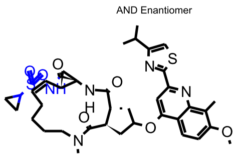<br>[*]NS(=O)(=O)C1[*][*]<br>1 | -0.825 | 0 out of 4 |
| SCFP_6 | 306578635 | 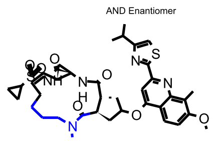<br>[*]CCCN(C)C(=[*])[*] | -0.825 | 0 out of 4 |
| SCFP_6 | 1260369147 | 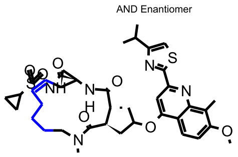<br>[*]CCC\C=C/[*] | -0.674 | 0 out of 3 |

# Flavonoid-1

# TOPKAT_Skin_Irritancy_None_vs_Irritant

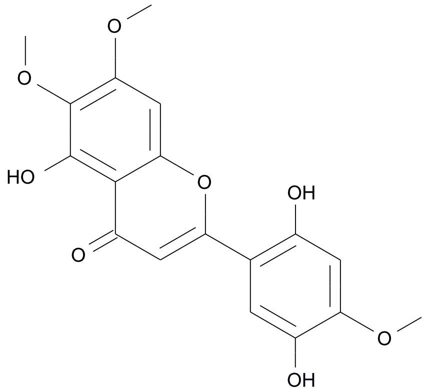

$C_{18}H_{16}O_8$

Molecular Weight: 360.31483

ALogP: 2.361

Rotatable Bonds: 4

Acceptors: 8

Donors: 3

## Structural Similar Compounds

| Name | Benzenesulfonamide, 4-amino-N-(5,6-dimethoxy-4-pyrimidinyl)- | 2-Anthracenesulfonic acid, 1-amino-9,10-dihydro-9,10-dioxo-4-(2,4,6 -trimethylanilino)-, monosodium salt | Benzenesulfonic acid, 2-anilino-5-nitro- |
|---|---|---|---|
| Structure | 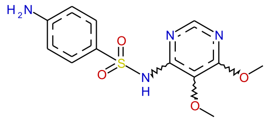 | 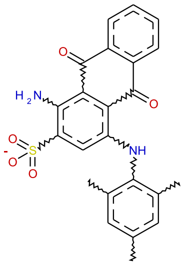 | 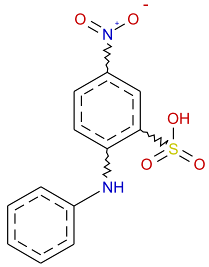 |
| Actual Endpoint | Irritant | Irritant | Irritant |
| Predicted Endpoint | Non-Irritant | Non-Irritant | Non-Irritant |
| Distance | 0.769 | 0.782 | 0.828 |
| Reference | FCTXAV 14,307,76 | 85JCAE "Prehled Prumyslove Toxikologie; Organicke Latky," Marhold, J., Prague , Czechoslovakia, Avicenum, 1986 Volume(issue)/page/year: -,1327,1986 | 85JCAE "Prehled Prumyslove Toxikologie; Organicke Latky," Marhold, J., Prague , Czechoslovakia, Avicenum, 1986 Volume(issue)/page/year: -,1061,1986 |

## Model Prediction

Prediction: Non-Irritant

Probability: 0.968

Enrichment: 1.05

Bayesian Score: -1.14

Mahalanobis Distance: 8.88

Mahalanobis Distance p-value: 0.487

Prediction: Positive if the Bayesian score is above the estimated best cutoff value from minimizing the false positive and false negative rate.
Probability: The esimated probability that the sample is in the positive category. This assumes that the Bayesian score follows a normal distribution and is different from the prediction using a cutoff.
Enrichment: An estimate of enrichment, that is, the increased likelihood (versus random) of this sample being in the category.
Bayesian Score: The standard Laplacian-modified Bayesian score.
Mahalanobis Distance: The Mahalanobis distance (MD) is the distance to the center of the training data. The larger the MD, the less trustworthy the prediction.
Mahalanobis Distance p-value: The p-value gives the fraction of training data with an MD greater than or equal to the one for the given sample, assuming normally distributed data. The smaller the p-value, the less trustworthy the prediciton. For highly non-normal X properties (e.g., fingerprints), the MD p-value is wildly inaccurate.

## Model Applicability

Unknown features are fingerprint features in the query molecule, but not found or appearing too infrequently in the training set.

1. All properties and OPS components are within expected ranges.

## Feature Contribution

| Top features for positive contribution | | | | |
|---|---|---|---|---|
| Fingerprint | Bit/Smiles | Feature Structure | Score | Irritant in training set |

| | | | | |
|---|---|---|---|---|
| FCFP_12 | 523826990 | 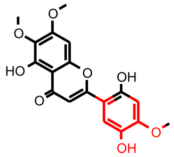 [*]O[c]1:[cH]:[*]:[c]([*]):[cH]:[c]:1O | 0.0756 | 6 out of 6 |
| FCFP_12 | 451847724 | 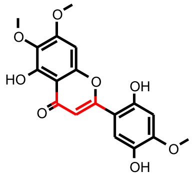 [*]C(=CC(=[*])[*])[*] | 0.0737 | 270 out of 274 |
| FCFP_12 | 301073077 | 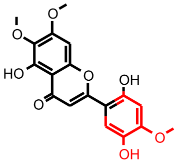 [*][c]1:[*]:[cH]:[c](O):[c](OC):[cH]:1 | 0.0734 | 5 out of 5 |

## Top Features for negative contribution

| Fingerprint | Bit/Smiles | Feature Structure | Score | Irritant in training set |
|---|---|---|---|---|
| FCFP_12 | 949015626 | 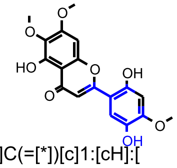 [*]C(=[*])[c]1:[cH]:[c](O):[c]([*]):[*]:[c]:1[*] | -0.222 | 2 out of 3 |
| FCFP_12 | -1604301295 | 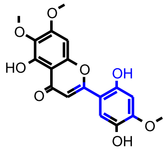 [*]C(=[*])[c]1:[cH]:[*]:[c]([*]):[cH]:[c]:1O | -0.18 | 22 out of 29 |

| | | | | |
|---|---|---|---|---|
| FCFP_12 | 7 | 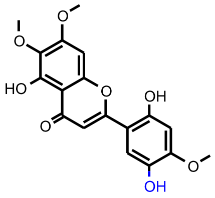<br>[*]O | -0.118 | 104 out of 128 |

# Simeprevir

# TOPKAT_Skin_Irritancy_None_vs_Irritant

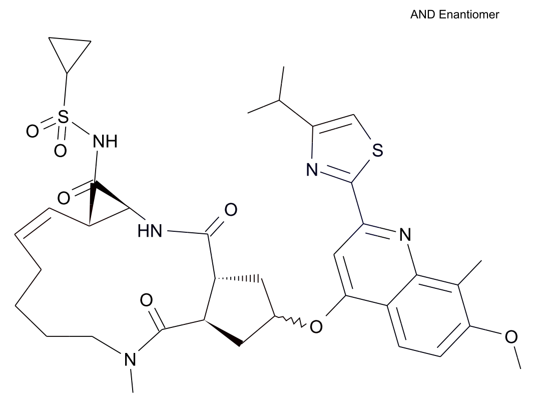


$C_{38}H_{47}N_5O_7S_2$
Molecular Weight: 749.93908
ALogP: 4.796
Rotatable Bonds: 8
Acceptors: 9
Donors: 2

## Model Prediction

Prediction: Non-Irritant
Probability: 0.969
Enrichment: 1.05
Bayesian Score: -1.05
Mahalanobis Distance: 16.8
Mahalanobis Distance p-value: 1.98e-021

Prediction: Positive if the Bayesian score is above the estimated best cutoff value from minimizing the false positive and false negative rate.
Probability: The esimated probability that the sample is in the positive category. This assumes that the Bayesian score follows a normal distribution and is different from the prediction using a cutoff.
Enrichment: An estimate of enrichment, that is, the increased likelihood (versus random) of this sample being in the category.
Bayesian Score: The standard Laplacian-modified Bayesian score.
Mahalanobis Distance: The Mahalanobis distance (MD) is the distance to the center of the training data. The larger the MD, the less trustworthy the prediction.
Mahalanobis Distance p-value: The p-value gives the fraction of training data with an MD greater than or equal to the one for the given sample, assuming normally distributed data. The smaller the p-value, the less trustworthy the prediciton. For highly non-normal X properties (e.g., fingerprints), the MD p-value is wildly inaccurate.

## Structural Similar Compounds

| Name | Anthraquinone, 3-methoxy-5,4'-iminobis(1-benzamido- | Benzenesulfonic acid, 2,2'-(1,4-anthraquinonylenediimino)bis(5-meth yl-, disodium salt | Butanamide, 2,2'-((3,3'-dichloro(1,1'-biphenyl)-4,4'-diyl)bis(azo)) bis(N-(2,4- dimethylphenyl)-3-oxo- |
|---|---|---|---|
| Structure | 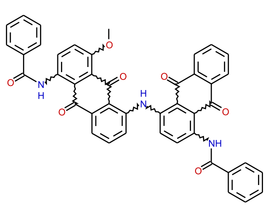 | 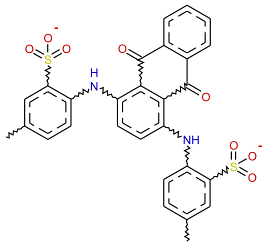 | 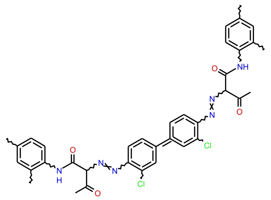 |
| Actual Endpoint | Irritant | Irritant | Irritant |
| Predicted Endpoint | Non-Irritant | Non-Irritant | Non-Irritant |
| Distance | 0.883 | 1.016 | 1.027 |
| Reference | 28ZPAK "Sbornik Vysledku Toxixologickeho Vysetreni Latek A Pripravku," Marhol d, J.V., Institut Pro Vychovu Vedoucicn Pracovniku Chemickeho Prumyclu Praha, Cz echoslovakia, 1972 Volume(issue)/page/year: -,114,1 | 85JCAE "Prehled Prumyslove Toxikologie; Organicke Latky," Marhold, J., Prague , Czechoslovakia, Avicenum, 1986 Volume(issue)/page/year: -,1330,1986 | NTIS** National Technical Information Service. (Springfield, VA 22161) Forme rly U.S. Clearinghouse for Scientific & Technical Information. Volume(issue)/pag e/year: OTS0555058 |

## Model Applicability

Unknown features are fingerprint features in the query molecule, but not found or appearing too infrequently in the training set.

1. OPS PC6 out of range. Value: 7.6964. Training min, max, SD, explained variance: -5.7225, 6.867, 1.786, 0.0403.
2. OPS PC22 out of range. Value: 5.13. Training min, max, SD, explained variance: -2.9568, 3.7845, 1.016, 0.0131.

## Feature Contribution

**Top features for positive contribution**

| Fingerprint | Bit/Smiles | Feature Structure | Score | Irritant in training set |
|---|---|---|---|---|

| | | | | |
|---|---|---|---|---|
| FCFP_12 | -415156552 | 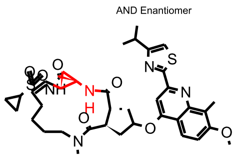 [*]N[C@]1(C[C@H]1[*])C(=[*])[*] | 0.0854 | 27 out of 27 |
| FCFP_12 | -53728878 | 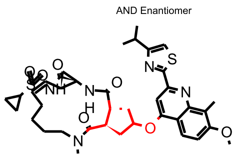 [*]OC1C[C@@H]([*])[C@@H](C1)C(=[*])[*] | 0.0845 | 21 out of 21 |
| FCFP_12 | 1186303932 | 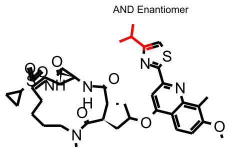 [*]:[c](:[*])C(C)C | 0.0838 | 18 out of 18 |

## Top Features for negative contribution

| Fingerprint | Bit/Smiles | Feature Structure | Score | Irritant in training set |
|---|---|---|---|---|
| FCFP_12 | 690481386 | 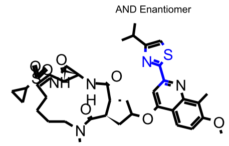 [*]:[c](:[*])[c]1:n:[*]:[*]:s:1 | -0.65 | 0 out of 1 |
| FCFP_12 | 566058135 | 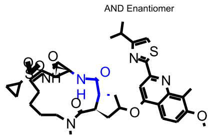 [*]NC(=O)C([*])[*] | -0.367 | 13 out of 21 |

| | | | | |
|---|---|---|---|---|
| FCFP_12 | -1320007763 | 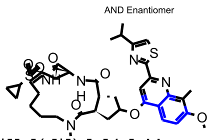 [*][c](:[*]):[c]1:[cH]:[cH]:[c]([*]):[*]:[c]:1:[*] | -0.0893 | 20 out of 24 |

# Flavonoid-1

# TOPKAT_Carcinogenic_Potency_TD50_Mouse

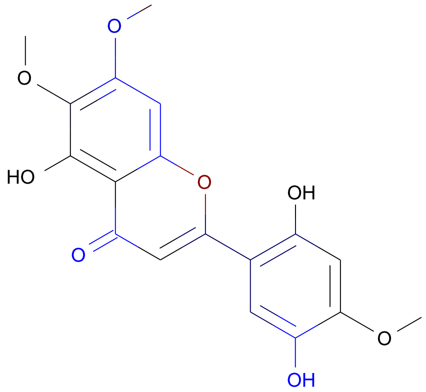

$C_{18}H_{16}O_8$

Molecular Weight: 360.31483

ALogP: 2.361

Rotatable Bonds: 4

Acceptors: 8

Donors: 3

## Model Prediction

Prediction: 111

Unit: mg/kg_body_weight/day

Mahalanobis Distance: 10.7

Mahalanobis Distance p-value: 0.00393

Mahalanobis Distance: The Mahalanobis distance (MD) is a generalization of the Euclidean distance that accounts for correlations among the X properties. It is calculated as the distance to the center of the training data. The larger the MD, the less trustworthy the prediction.

Mahalanobis Distance p-value: The p-value gives the fraction of training data with an MD greater than or equal to the one for the given sample, assuming normally distributed data. The smaller the p-value, the less trustworthy the prediciton. For highly non-normal X properties (e.g., fingerprints), the MD p-value is wildly inaccurate.

## Structural Similar Compounds

| Name | 542 | Ochratoxin A | Salicylazosulfapyridine |
|---|---|---|---|
| Structure | 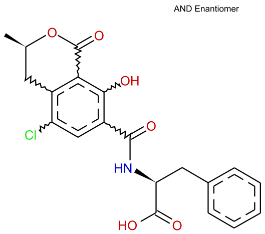  | 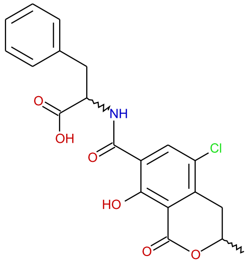 | 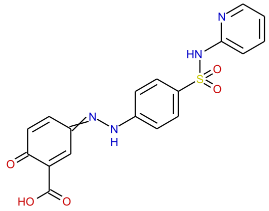 |
| Actual Endpoint (-log C) | 4.79932 | 4.79932 | 2.5034 |
| Predicted Endpoint (-log C) | 3.6353 | 3.6353 | 3.54214 |
| Distance | 0.621 | 0.621 | 0.655 |
| Reference | CPDB | CPDB | CPDB |

## Model Applicability

Unknown features are fingerprint features in the query molecule, but not found or appearing too infrequently in the training set.

1. All properties and OPS components are within expected ranges.
2. Unknown ECFP_2 feature: 1573564860: [*]O\C(=C/[*])\[c](:[*]):[*]

## Feature Contribution

### Top features for positive contribution

| Fingerprint | Bit/Smiles | Feature Structure | Score |
|---|---|---|---|
| ECFP_6 | 683445015 | 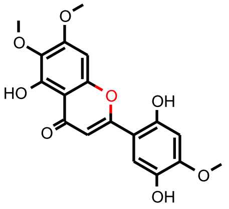 [*]O[*] | 0.136 |

| | | | |
|---|---|---|---|
| ECFP_6 | 734603939 | 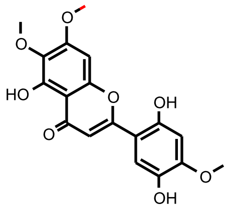 [*]C | 0.0424 |
| ECFP_6 | 1307307440 | 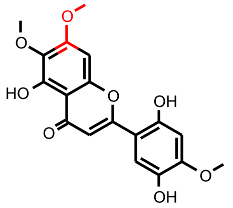 [*]:[c](:[*])OC | 0.0156 |
| **Top Features for negative contribution** | | | |
| **Fingerprint** | **Bit/Smiles** | **Feature Structure** | **Score** |
| ECFP_6 | 2106656448 | 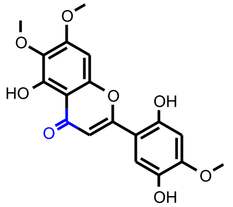 [*]C(=O)[*] | -0.275 |
| ECFP_6 | 2019062761 | 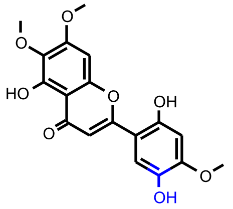 [*]:[c](:[*])O | -0.258 |
| ECFP_6 | 642810091 | 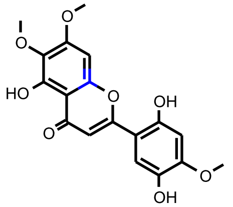 [*][c](:[*]):[*] | -0.247 |

# Simeprevir

# TOPKAT_Carcinogenic_Potency_TD50_Mouse

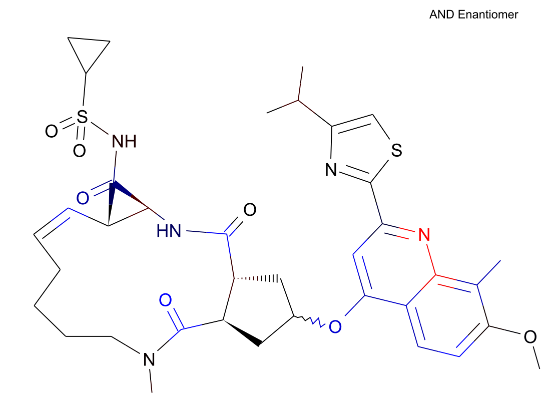


$C_{38}H_{47}N_5O_7S_2$

Molecular Weight: 749.93908

ALogP: 4.796

Rotatable Bonds: 8

Acceptors: 9

Donors: 2

## Structural Similar Compounds

| Name | 223 | D & C red no. 5 | Salicylazosulfapyridine |
|---|---|---|---|
| Structure | 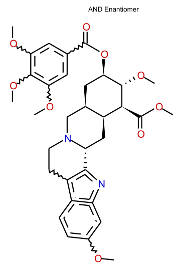 | 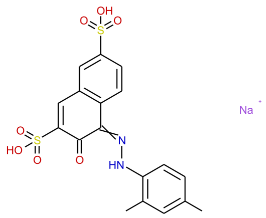 | 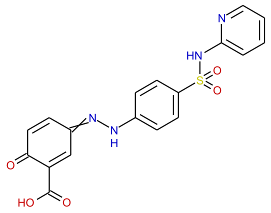 |
| Actual Endpoint (-log C) | 5.08368 | 2.80732 | 2.5034 |
| Predicted Endpoint (-log C) | 5.08273 | 3.78615 | 3.54214 |
| Distance | 0.926 | 1.173 | 1.177 |
| Reference | CPDB | CPDB | CPDB |

## Model Prediction

Prediction: 2.01

Unit: mg/kg_body_weight/day

Mahalanobis Distance: 16.8

Mahalanobis Distance p-value: 5.81e-019

Mahalanobis Distance: The Mahalanobis distance (MD) is a generalization of the Euclidean distance that accounts for correlations among the X properties. It is calculated as the distance to the center of the training data. The larger the MD, the less trustworthy the prediction.
Mahalanobis Distance p-value: The p-value gives the fraction of training data with an MD greater than or equal to the one for the given sample, assuming normally distributed data. The smaller the p-value, the less trustworthy the prediciton. For highly non-normal X properties (e.g., fingerprints), the MD p-value is wildly inaccurate.

## Model Applicability

Unknown features are fingerprint features in the query molecule, but not found or appearing too infrequently in the training set.

1. OPS PC14 out of range. Value: -4.1139. Training min, max, SD, explained variance: -3.6133, 3.7483, 1.312, 0.0215.
2. OPS PC16 out of range. Value: 4.4468. Training min, max, SD, explained variance: -3.1026, 4.016, 1.245, 0.0193.
3. Unknown ECFP_2 feature: -309676563: [*]N[C@]1(C[C@H]1[*])C(=[*])[*]
4. Unknown ECFP_2 feature: -1049290660: [*]C1([*])C[C@H]1C=[*]
5. Unknown ECFP_2 feature: -1818486371: [*]NC(=O)C1([*])[*][*]1
6. Unknown ECFP_2 feature: 946167604: [*]C(=[*])NS(=[*])(=[*])[*]
7. Unknown ECFP_2 feature: 1310940530: [*]C([*])O[c](:[*]):[*]
8. Unknown ECFP_2 feature: 1411720546: [*]C([*])[c]1:[cH]:[*]:[*]:n:1
9. Unknown ECFP_2 feature: 733491677: [*]:[c](:[*])C(C)C
10. Unknown ECFP_2 feature: 2121941848: [*]NS(=O)(=O)C1[*][*]1
11. Unknown ECFP_2 feature: -622223421: [*]S(=[*])(=[*])C1CC1

## Feature Contribution

### Top features for positive contribution

| Fingerprint | Bit/Smiles | Feature Structure | Score |
|---|---|---|---|

| | | | |
|---|---|---|---|
| ECFP_6 | 655739385 | 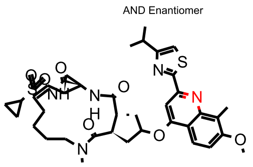<br>[*]:n:[*] | 0.229 |
| ECFP_6 | 834876373 | 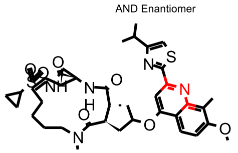<br>[*][c](:[*]):n:[c](:[<br>*]):[*] | 0.163 |
| ECFP_6 | 657586427 | 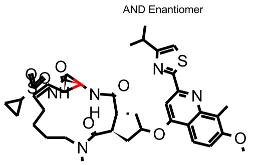<br>[*]C1([*])[*][*]1 | 0.0789 |
| **Top Features for negative contribution** | | | |
| **Fingerprint** | **Bit/Smiles** | **Feature Structure** | **Score** |
| ECFP_6 | 2106656448 | 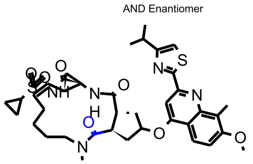<br>[*]C(=O)[*] | -0.275 |
| ECFP_6 | 1996767644 | 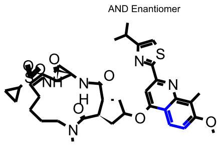<br>[*]:[cH]:[cH]:[c](:[*<br>]):[*] | -0.251 |

| | | | |
|---|---|---|---|
| ECFP_6 | 642810091 | 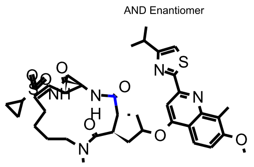<br>[*][c](:[*]):[*] | -0.247 |

# Flavonoid-1

# TOPKAT_Carcinogenic_Potency_TD50_Rat

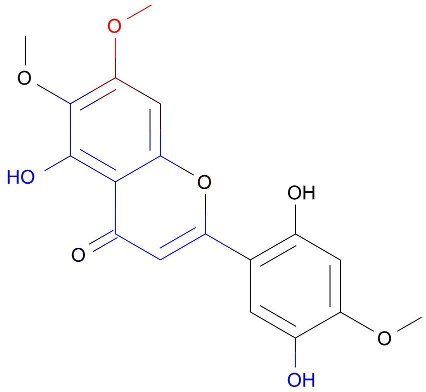


$C_{18}H_{16}O_8$

Molecular Weight: 360.31483

ALogP: 2.361

Rotatable Bonds: 4

Acceptors: 8

Donors: 3

## Structural Similar Compounds

| Name | 542 | Ochratoxin A | Salicylazosulfapyridine |
|---|---|---|---|
| Structure | 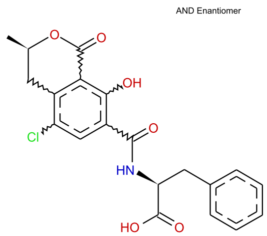 | 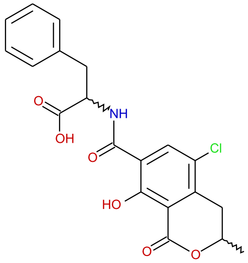 | 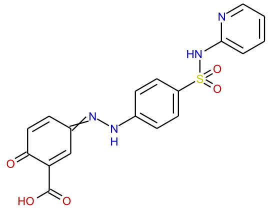 |
| Actual Endpoint (-log C) | 6.59334 | 6.47264 | 2.39891 |
| Predicted Endpoint (-log C) | 5.06501 | 5.06501 | 3.17598 |
| Distance | 0.601 | 0.601 | 0.623 |
| Reference | CPDB | CPDB | CPDB |

## Model Applicability

Unknown features are fingerprint features in the query molecule, but not found or appearing too infrequently in the training set.

1. All properties and OPS components are within expected ranges.

## Model Prediction

Prediction: 13.6

Unit: mg/kg_body_weight/day

Mahalanobis Distance: 11.7

Mahalanobis Distance p-value: 0.00204

Mahalanobis Distance: The Mahalanobis distance (MD) is a generalization of the Euclidean distance that accounts for correlations among the X properties. It is calculated as the distance to the center of the training data. The larger the MD, the less trustworthy the prediction.
Mahalanobis Distance p-value: The p-value gives the fraction of training data with an MD greater than or equal to the one for the given sample, assuming normally distributed data. The smaller the p-value, the less trustworthy the prediciton. For highly non-normal X properties (e.g., fingerprints), the MD p-value is wildly inaccurate.

## Feature Contribution

### Top features for positive contribution

| Fingerprint | Bit/Smiles | Feature Structure | Score |
|---|---|---|---|
| FCFP_6 | 136627117 | 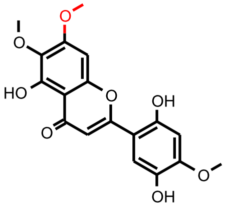 [*]OC | 0.69 |

| | | | |
|---|---|---|---|
| FCFP_6 | 1 | 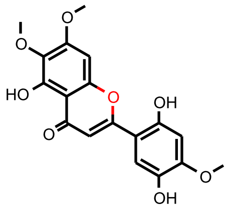<br>[*]O[*] | 0.234 |
| FCFP_6 | 203677720 | 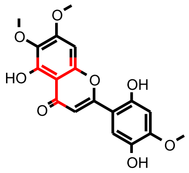<br>[*]C(=[*])[c](:[c]([*]):[*]):[c]([*]):[*] | 0.137 |
| **Top Features for negative contribution** | | | |
| **Fingerprint** | **Bit/Smiles** | **Feature Structure** | **Score** |
| FCFP_6 | 451847724 | 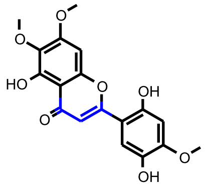<br>[*]C(=CC(=[*])[*])[*] | -0.436 |
| FCFP_6 | 7 | 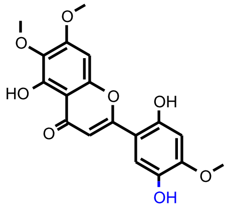<br>[*]O | -0.372 |
| FCFP_6 | 16 | 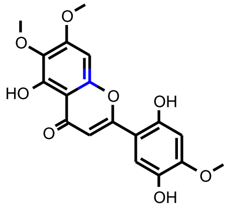<br>[*][c](:[*]):[*] | -0.354 |

# Simeprevir

# TOPKAT_Carcinogenic_Potency_TD50_Rat

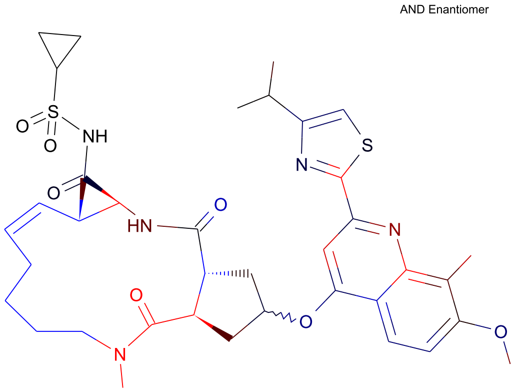


$C_{38}H_{47}N_5O_7S_2$

Molecular Weight: 749.93908

ALogP: 4.796

Rotatable Bonds: 8

Acceptors: 9

Donors: 2

## Model Prediction

Prediction: 0.28

Unit: mg/kg_body_weight/day

Mahalanobis Distance: 24.1

Mahalanobis Distance p-value: 1.44e-046

Mahalanobis Distance: The Mahalanobis distance (MD) is a generalization of the Euclidean distance that accounts for correlations among the X properties. It is calculated as the distance to the center of the training data. The larger the MD, the less trustworthy the prediction.
Mahalanobis Distance p-value:  The p-value gives the fraction of training data with an MD greater than or equal to the one for the given sample, assuming normally distributed data. The smaller the p-value, the less trustworthy the prediciton. For highly non-normal X properties (e.g., fingerprints), the MD p-value is wildly inaccurate.

## Structural Similar Compounds

| Name | 411 | 223 | 188 |
|---|---|---|---|
| Structure | 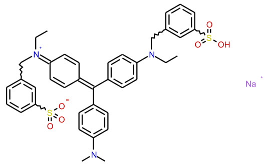 | 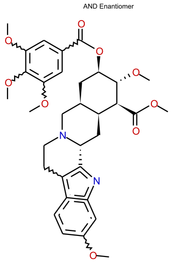 | 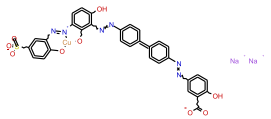 |
| Actual Endpoint (-log C) | 3.06566 | 6.29867 | 5.5378 |
| Predicted Endpoint (-log C) | 4.8672 | 7.5657 | 5.71925 |
| Distance | 0.846 | 0.861 | 0.891 |
| Reference | CPDB | CPDB | CPDB |

## Model Applicability

Unknown features are fingerprint features in the query molecule, but not found or appearing too infrequently in the training set.

1. OPS PC27 out of range. Value: -3.3094. Training min, max, SD, explained variance: -2.8642, 4.2058, 1.002, 0.0096.

## Feature Contribution

**Top features for positive contribution**

| Fingerprint | Bit/Smiles | Feature Structure | Score |
|---|---|---|---|
| FCFP_6 | 136627117 | 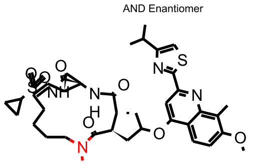 [*]OC | 0.69 |

| | | | |
|---|---|---|---|
| FCFP_6 | 565998553 | 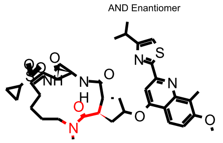 [*]C([*])C(=O)N([*])[*] | 0.357 |
| FCFP_6 | 690511177 | 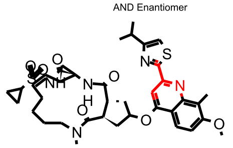 [*]:[cH]:[c](:n:[*])[c](:[*]):[*] | 0.293 |
| **Top Features for negative contribution** | | | |
| **Fingerprint** | **Bit/Smiles** | **Feature Structure** | **Score** |
| FCFP_6 | 1175638033 | 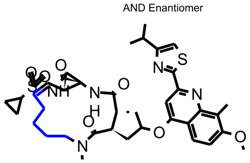 [*]CCCCC=[*] | -0.512 |
| FCFP_6 | 451847724 | 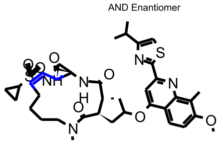 [*]C(=CC(=[*])[*])[*] | -0.436 |
| FCFP_6 | 16 | 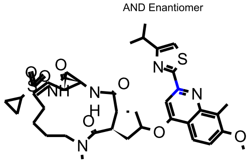 [*][c](:[*]):[*] | -0.354 |

# Flavonoid-1

# TOPKAT_Chronic_LOAEL

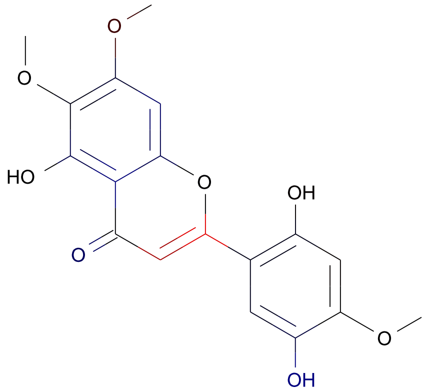


$C_{18}H_{16}O_8$

Molecular Weight: 360.31483

ALogP: 2.361

Rotatable Bonds: 4

Acceptors: 8

Donors: 3

## Model Prediction

Prediction: 0.0624

Unit: g/kg_body_weight

Mahalanobis Distance: 24.3

Mahalanobis Distance p-value: 2.58e-014

Mahalanobis Distance: The Mahalanobis distance (MD) is a generalization of the Euclidean distance that accounts for correlations among the X properties. It is calculated as the distance to the center of the training data. The larger the MD, the less trustworthy the prediction.

Mahalanobis Distance p-value: The p-value gives the fraction of training data with an MD greater than or equal to the one for the given sample, assuming normally distributed data. The smaller the p-value, the less trustworthy the prediciton. For highly non-normal X properties (e.g., fingerprints), the MD p-value is wildly inaccurate.

## Structural Similar Compounds

| Name | CHLORSULFURON | OLSALAZINE.NA | QUERCETIN |
|---|---|---|---|
| Structure | 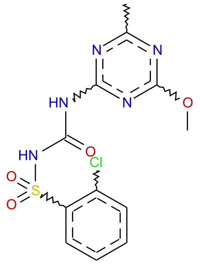 | 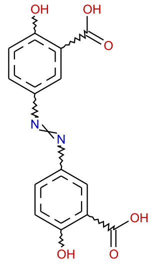 | 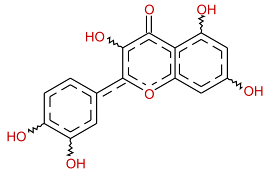 |
| Actual Endpoint (-log C) | 4.15566 | 3.17932 | 2.87829 |
| Predicted Endpoint (-log C) | 3.79771 | 2.89417 | 3.12498 |
| Distance | 0.595 | 0.618 | 0.651 |
| Reference | EPA COVER SHEET 0027;880301;(1) | NDA-19715 | NTP 409 79 |

## Model Applicability

Unknown features are fingerprint features in the query molecule, but not found or appearing too infrequently in the training set.

1. All properties and OPS components are within expected ranges.
2. Unknown ECFP_6 feature: -570915357: [*]O[c](:[cH]:[*]):[c]([*]):[*]
3. Unknown ECFP_6 feature: -813997308: [*]C(=[*])[c](:[c]([*]):[*]):[c]([*]):[*]
4. Unknown ECFP_6 feature: -1660913849: [*][c](:[*]):[c](O):[c]([*]):[*]
5. Unknown ECFP_6 feature: -1531301414: [*]O[c](:[c]([*]):[*]):[c]([*]):[*]
6. Unknown ECFP_6 feature: -560785749: [*]C(=[*])O[c](:[*]):[*]
7. Unknown ECFP_6 feature: 1573564860: [*]O\C(=C/[*])\[c](:[*]):[*]
8. Unknown ECFP_6 feature: 1299558496: [*]=CC(=O)[c](:[*]):[*]
9. Unknown ECFP_6 feature: 464808839: [*]C(=CC(=[*])[*])[*]
10. Unknown ECFP_6 feature: 1307307440: [*]:[c](:[*])OC
11. Unknown ECFP_6 feature: 2019062761: [*]:[c](:[*])O

## Feature Contribution

**Top features for positive contribution**

| Fingerprint | Bit/Smiles | Feature Structure | Score |
|---|---|---|---|

| | | | |
|---|---|---|---|
| FCFP_6 | 451847724 | 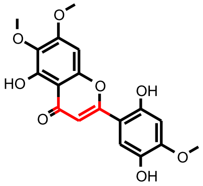<br>[*]C(=CC(=[*])[*])[*] | 0.16 |
| ECFP_6 | 683445015 | 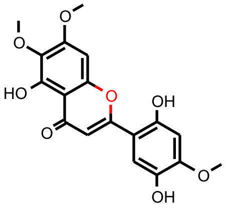<br>[*]O[*] | 0.0734 |
| FCFP_6 | 1036089772 | 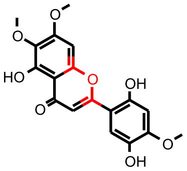<br>[*]C(=[*])O[c](:[*]):<br>[*] | 0.073 |
| **Top Features for negative contribution** | | | |
| **Fingerprint** | **Bit/Smiles** | **Feature Structure** | **Score** |
| ECFP_6 | 2106656448 | 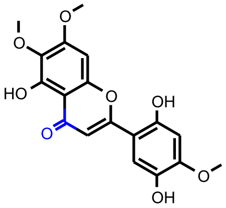<br>[*]C(=O)[*] | -0.11 |
| FCFP_6 | 1 | 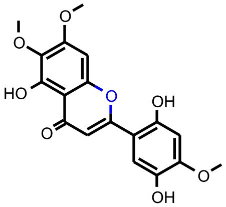<br>[*]O[*] | -0.102 |

| | | | |
|---|---|---|---|
| FCFP_6 | 203677720 | 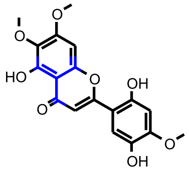 [*]C(=[*])[c](:[c]([*]):[*]):[c]([*]):[*] | -0.0713 |

# Simeprevir

# TOPKAT_Chronic_LOAEL

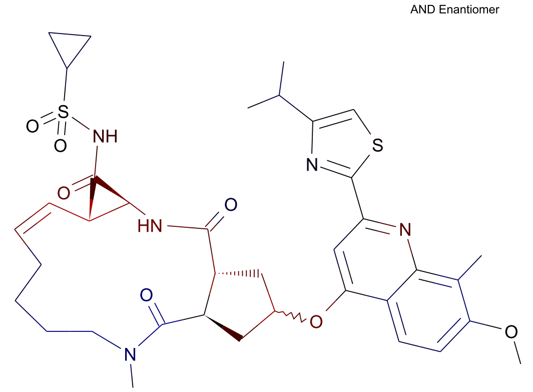


$C_{38}H_{47}N_5O_7S_2$

Molecular Weight: 749.93908

ALogP: 4.796

Rotatable Bonds: 8

Acceptors: 9

Donors: 2

## Model Prediction

Prediction: 0.00211

Unit: g/kg_body_weight

Mahalanobis Distance: 55.7

Mahalanobis Distance p-value: 1.58e-065

Mahalanobis Distance: The Mahalanobis distance (MD) is a generalization of the Euclidean distance that accounts for correlations among the X properties. It is calculated as the distance to the center of the training data. The larger the MD, the less trustworthy the prediction.

Mahalanobis Distance p-value:  The p-value gives the fraction of training data with an MD greater than or equal to the one for the given sample, assuming normally distributed data. The smaller the p-value, the less trustworthy the prediciton. For highly non-normal X properties (e.g., fingerprints), the MD p-value is wildly inaccurate.

## Structural Similar Compounds

| Name | RESERPINE | DIARYLANILIDE YELLOW | C.I.PIGMENT RED 23 |
|---|---|---|---|
| Structure | 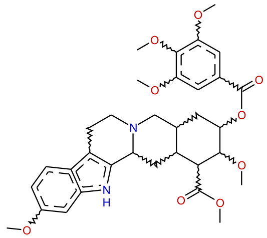 | 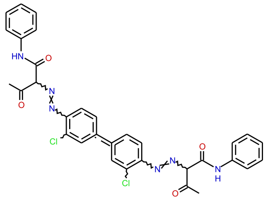 | 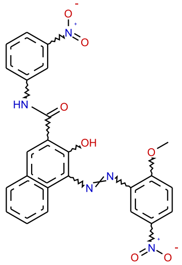 |
| Actual Endpoint (-log C) | 6.38645 | 2.70208 | 2.28997 |
| Predicted Endpoint (-log C) | 5.548 | 3.76154 | 3.52921 |
| Distance | 0.885 | 0.887 | 0.922 |
| Reference | NTP 193 22 | NTP 30 C-4 | NTP 411 146 |

## Model Applicability

Unknown features are fingerprint features in the query molecule, but not found or appearing too infrequently in the training set.

1. OPS PC1 out of range. Value: 18.64. Training min, max, SD, explained variance: -9.2986, 15.594, 5, 0.1094.
2. Unknown FCFP_2 feature: 1018942292: [*]NS(=O)(=O)C1[*][*]1
3. Unknown ECFP_6 feature: -154530762: [*]N[*]
4. Unknown ECFP_6 feature: 914325265: [*]:s:[*]
5. Unknown ECFP_6 feature: -797085356: [*]S(=[*])(=[*])[*]
6. Unknown ECFP_6 feature: -2095963820: [*][C@@H]1[*][*]C[C@H]1C(=[*])[*]
7. Unknown ECFP_6 feature: -867777309: [*]NC(=O)C([*])[*]
8. Unknown ECFP_6 feature: -1338907019: [*]C(=[*])NC1([*])[*][*]1
9. Unknown ECFP_6 feature: -309676563: [*]N[C@]1(C[C@H]1[*])C(=[*])[*]
10. Unknown ECFP_6 feature: 413587124: [*][C@@H]1CC1([*])[*]
11. Unknown ECFP_6 feature: -1049290660: [*]C1([*])C[C@H]1C=[*]
12. Unknown ECFP_6 feature: 890368401: [*]\C=C/C1[*][*]1
13. Unknown ECFP_6 feature: 360408239: [*]C\C=C/[*]
14. Unknown ECFP_6 feature: -1331088410: [*]CCC=[*]
15. Unknown ECFP_6 feature: -1332781180: [*]CCC[*]
16. Unknown ECFP_6 feature: -757679000: [*]CCN([*])[*]
17. Unknown ECFP_6 feature: 1616402542: [*]CN(C)C(=[*])[*]
18. Unknown ECFP_6 feature: 1526862590: [*]C([*])C(=O)N([*])[*]
19. Unknown ECFP_6 feature: -801490360: [*][C@H]1[*][*]C([*])C1

20. Unknown ECFP_6 feature: 1480368712: [*]OC1C[*][*]C1
21. Unknown ECFP_6 feature: -1818486371: [*]NC(=O)C1([*])[*][*]1
22. Unknown ECFP_6 feature: 946167604: [*]C(=[*])NS(=[*])(=[*])[*]
23. Unknown ECFP_6 feature: 866343404: [*]N([*])C
24. Unknown ECFP_6 feature: 1310940530: [*]C([*])O[c](:[*]):[*]
25. Unknown ECFP_6 feature: -428002189: [*]:[cH]:[c](:n:[*])[c](:[*]):[*]
26. Unknown ECFP_6 feature: 1333660716: [*][c](:[*]):[c](:[cH]:[*]):[c](:[*]):[*]
27. Unknown ECFP_6 feature: 1576255326: [*][c](:[*]):[c](C):[c](:[*]):[*]
28. Unknown ECFP_6 feature: 1048320787: [*][c](:[*]):[c](:n:[*]):[c](:[*]):[*]
29. Unknown ECFP_6 feature: -1426923364: [*][c]1:[*]:[*]:[cH]:s:1
30. Unknown ECFP_6 feature: -253227249: [*]:[c](:[*])[c]1:n:[*]:[*]:s:1
31. Unknown ECFP_6 feature: 1411720546: [*]C([*])[c]1:[cH]:[*]:[*]:n:1
32. Unknown ECFP_6 feature: -224638920: [*][c]1:[*]:[*]:s:[cH]:1
33. Unknown ECFP_6 feature: 733491677: [*]:[c](:[*])C(C)C
34. Unknown ECFP_6 feature: 1307307440: [*]:[c](:[*])OC
35. Unknown ECFP_6 feature: 2121941848: [*]NS(=O)(=O)C1[*][*]1
36. Unknown ECFP_6 feature: 2102150379: [*]S(=[*])(=O)[*]
37. Unknown ECFP_6 feature: -622223421: [*]S(=[*])(=[*])C1CC1

# Feature Contribution

| Top features for positive contribution | | | |
|---|---|---|---|
| **Fingerprint** | **Bit/Smiles** | **Feature Structure** | **Score** |
| FCFP_6 | 451847724 | 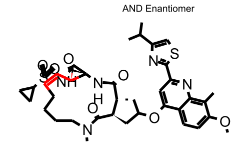  [*]C(=CC(=[*])[*])[*] | 0.16 |
| ECFP_6 | -167460056 | 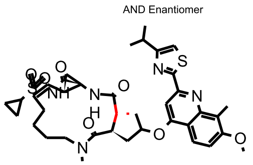  [*]C([*])[*] | 0.136 |

| | | | |
|---|---|---|---|
| FCFP_6 | 3 | 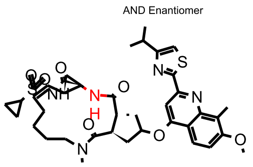 [*]N[*] | 0.0924 |
| **Top Features for negative contribution** | | | |
| **Fingerprint** | **Bit/Smiles** | **Feature Structure** | **Score** |
| ECFP_6 | 2106656448 | 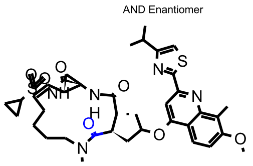 [*]C(=O)[*] | -0.11 |
| FCFP_6 | 1 | 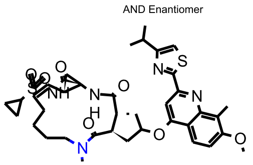 [*]O[*] | -0.102 |
| FCFP_6 | 136597326 | 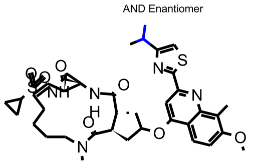 [*]C([*])C | -0.0815 |

# Flavonoid-1

# TOPKAT_Rat_Maximum_Tolerated_Dose_Feed

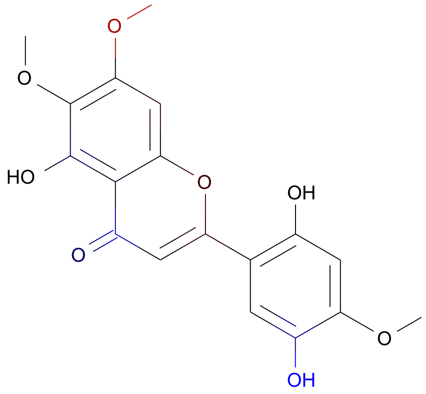

$C_{18}H_{16}O_8$

Molecular Weight: 360.31483

ALogP: 2.361

Rotatable Bonds: 4

Acceptors: 8

Donors: 3

## Model Prediction

Prediction: 0.35

Unit: g/kg_body_weight

Mahalanobis Distance: 5.99

Mahalanobis Distance p-value: 0.645

Mahalanobis Distance: The Mahalanobis distance (MD) is a generalization of the Euclidean distance that accounts for correlations among the X properties. It is calculated as the distance to the center of the training data. The larger the MD, the less trustworthy the prediction.
Mahalanobis Distance p-value: The p-value gives the fraction of training data with an MD greater than or equal to the one for the given sample, assuming normally distributed data. The smaller the p-value, the less trustworthy the prediciton. For highly non-normal X properties (e.g., fingerprints), the MD p-value is wildly inaccurate.

## Structural Similar Compounds

| Name | QUERCETIN | SALICYLAZOSULFAPYRIDINE | FUROSEMIDE |
|---|---|---|---|
| Structure | 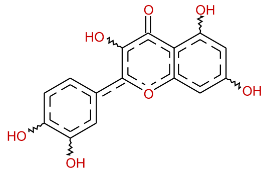 | 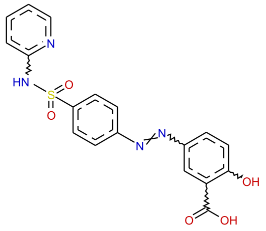 | 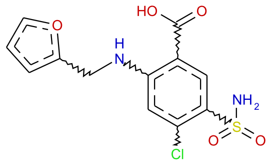 |
| Actual Endpoint (-log C) | 2.2016 | 3.375 | 4.04236 |
| Predicted Endpoint (-log C) | 2.27782 | 2.80292 | 2.8614 |
| Distance | 0.513 | 0.639 | 0.698 |
| Reference | NCI/NTP TR-409 | NCI/NTP TR-457 | NCI/NTP TR-356 |

## Model Applicability

Unknown features are fingerprint features in the query molecule, but not found or appearing too infrequently in the training set.

1. All properties and OPS components are within expected ranges.

## Feature Contribution

| Top features for positive contribution | | | |
|---|---|---|---|
| **Fingerprint** | **Bit/Smiles** | **Feature Structure** | **Score** |
| FCFP_2 | 136627117 | 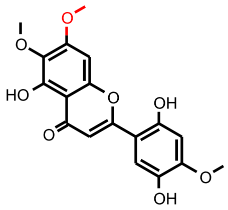 [*]OC | 0.173 |

| | | | |
|---|---|---|---|
| FCFP_2 | 1036089772 | 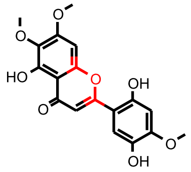 [*]C(=[*])O[c](:[*]):[*] | 0.0749 |
| FCFP_2 | 332760439 | 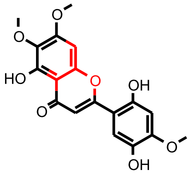 [*]O[c](:[cH]:[*]):[c]([*]):[*] | 0.0611 |

## Top Features for negative contribution

| **Fingerprint** | **Bit/Smiles** | **Feature Structure** | **Score** |
|---|---|---|---|
| FCFP_2 | 7 | 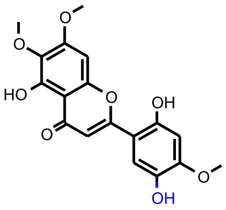 [*]O | -0.214 |
| FCFP_2 | -549108873 | 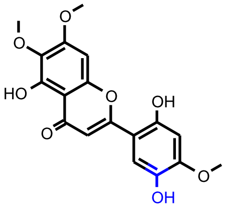 [*]:[c](:[*])O | -0.127 |
| FCFP_2 | 1872154524 | 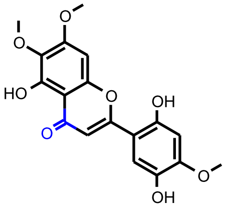 [*]C(=O)[*] | -0.105 |

# Simeprevir

# TOPKAT_Rat_Maximum_Tolerated_Dose_Feed

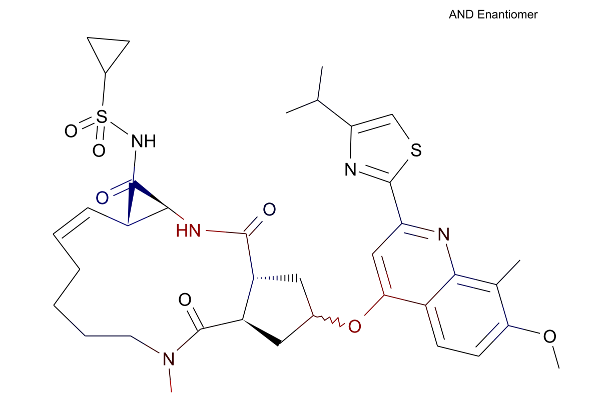


$C_{38}H_{47}N_5O_7S_2$

Molecular Weight: 749.93908

ALogP: 4.796

Rotatable Bonds: 8

Acceptors: 9

Donors: 2

## Model Prediction

Prediction: 0.00297

Unit: g/kg_body_weight

Mahalanobis Distance: 14.2

Mahalanobis Distance p-value: 5.46e-013

Mahalanobis Distance: The Mahalanobis distance (MD) is a generalization of the Euclidean distance that accounts for correlations among the X properties. It is calculated as the distance to the center of the training data. The larger the MD, the less trustworthy the prediction.
Mahalanobis Distance p-value: The p-value gives the fraction of training data with an MD greater than or equal to the one for the given sample, assuming normally distributed data. The smaller the p-value, the less trustworthy the prediciton. For highly non-normal X properties (e.g., fingerprints), the MD p-value is wildly inaccurate.

## Structural Similar Compounds

| Name | C.I.PIGMENT RED 23 | RESERPINE | SALICYLAZOSULFAPYRIDINE |
|---|---|---|---|
| Structure | 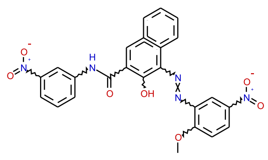 | 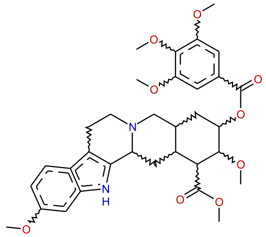 | 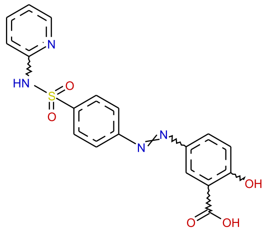 |
| Actual Endpoint (-log C) | 2.30052 | 6.13118 | 3.375 |
| Predicted Endpoint (-log C) | 3.55333 | 4.38304 | 2.80292 |
| Distance | 0.861 | 0.934 | 1.035 |
| Reference | NCI/NTP TR-411 | NCI/NTP TR-193 | NCI/NTP TR-457 |

## Model Applicability

Unknown features are fingerprint features in the query molecule, but not found or appearing too infrequently in the training set.

1. Molecular_Weight out of range. Value: 749.94. Training min, max, mean, SD: 74.122, 731.95, 245.25, 106.4.
2. OPS PC9 out of range. Value: 5.11. Training min, max, SD, explained variance: -2.8548, 3.3954, 1.263, 0.0360.
3. OPS PC12 out of range. Value: -3.0299. Training min, max, SD, explained variance: -2.364, 2.9228, 1.079, 0.0263.
4. OPS PC14 out of range. Value: 4.236. Training min, max, SD, explained variance: -2.0656, 3.3808, 1.011, 0.0231.
5. Unknown FCFP_2 feature: 690481386: [*]:[c](:[*])[c]1:n:[*]:[*]:s:1
6. Unknown FCFP_2 feature: 1018942292: [*]NS(=O)(=O)C1[*][*]1

## Feature Contribution

**Top features for positive contribution**

| Fingerprint | Bit/Smiles | Feature Structure | Score |
|---|---|---|---|

| | | | |
|---|---|---|---|
| FCFP_2 | 136627117 | 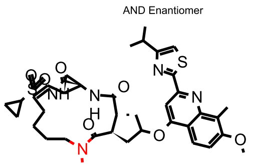 [*]OC | 0.173 |
| FCFP_2 | -885550502 | 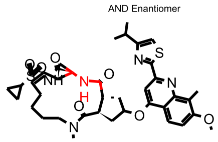 [*]C(=[*])NC1([*])[*] [*]1 | 0.115 |
| FCFP_2 | 1036089772 | 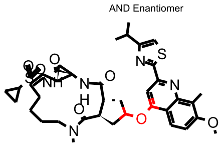 [*]C(=[*])O[c](:[*]): [*] | 0.0749 |

## Top Features for negative contribution

| **Fingerprint** | **Bit/Smiles** | **Feature Structure** | **Score** |
|---|---|---|---|
| FCFP_2 | -1272798659 | 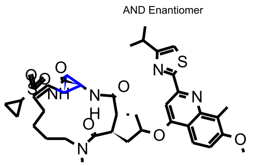 [*][C@@H]1CC1([*])[*] | -0.111 |
| FCFP_2 | 1872154524 | 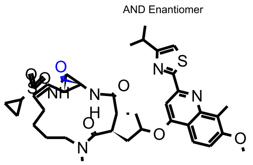 [*]C(=O)[*] | -0.105 |

| | | | |
|---|---|---|---|
| FCFP_2 | 203677720 | 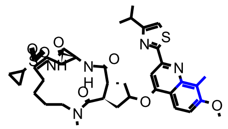[*]C(=[*])[c](:[c]([* ]):[*]):[c]([*]):[*] | -0.0829 |

# Flavonoid-1 TOPKAT_Rat_Maximum_Tolerated_Dose_Gavage

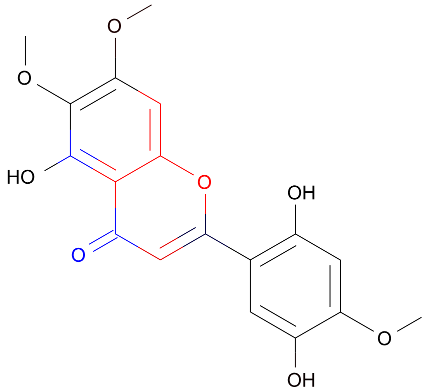

$C_{18}H_{16}O_8$
Molecular Weight: 360.31483
ALogP: 2.361
Rotatable Bonds: 4
Acceptors: 8
Donors: 3

## Model Prediction

Prediction: 5.44e-005
Unit: g/kg_body_weight
Mahalanobis Distance: 11.3
Mahalanobis Distance p-value: 9.31e-008

Mahalanobis Distance: The Mahalanobis distance (MD) is a generalization of the Euclidean distance that accounts for correlations among the X properties. It is calculated as the distance to the center of the training data. The larger the MD, the less trustworthy the prediction.
Mahalanobis Distance p-value: The p-value gives the fraction of training data with an MD greater than or equal to the one for the given sample, assuming normally distributed data. The smaller the p-value, the less trustworthy the prediciton. For highly non-normal X properties (e.g., fingerprints), the MD p-value is wildly inaccurate.

## Structural Similar Compounds

| Name | OCHRATOXIN | PENICILLIN VK | SULFISOOXAZOLE |
|---|---|---|---|
| Structure | 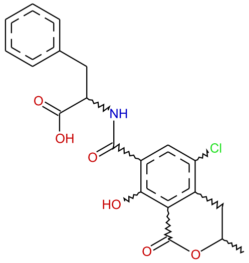 | 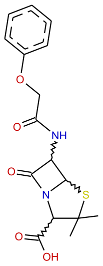 | 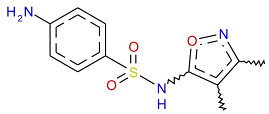 |
| Actual Endpoint (-log C) | 6.28396 | 2.54455 | 2.82494 |
| Predicted Endpoint (-log C) | 5.12358 | 3.9702 | 3.0705 |
| Distance | 0.613 | 0.796 | 0.929 |
| Reference | NCI/NTP TR-358 | NCI/NTP TR-336 | NCI/NTP TR-138 |

## Model Applicability

Unknown features are fingerprint features in the query molecule, but not found or appearing too infrequently in the training set.

1. Num_H_Acceptors out of range. Value: 8. Training min, max, mean, SD: 0, 6, 1.6146, 1.644.
2. OPS PC9 out of range. Value: 4.6121. Training min, max, SD, explained variance: -2.7086, 2.9267, 1.019, 0.0321.
3. Unknown FCFP_2 feature: -1678245750: [*]O\C(=C/[*])\[c](:[*]):[*]
4. Unknown FCFP_2 feature: -1549192822: [*]=CC(=O)[c](:[*]):[*]

## Feature Contribution

**Top features for positive contribution**

| Fingerprint | Bit/Smiles | Feature Structure | Score |
|---|---|---|---|
| FCFP_2 | 332760439 | 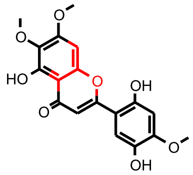 [*]O[c](:[cH]:[*]):[c]([*]):[*] | 0.672 |

| | | | |
|---|---|---|---|
| FCFP_2 | 1 | 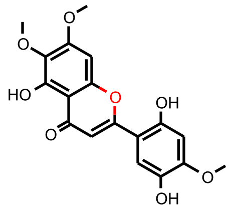 [*]O[*] | 0.511 |
| FCFP_2 | 451847724 | 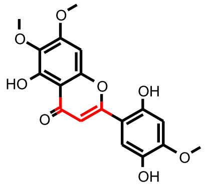 [*]C(=CC(=[*])[*])[*] | 0.225 |

**Top Features for negative contribution**

| Fingerprint | Bit/Smiles | Feature Structure | Score |
|---|---|---|---|
| FCFP_2 | 203677720 | 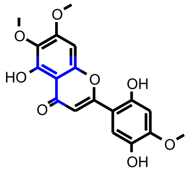 [*]C(=[*])[c](:[c]([* ]):[*]):[c]([*]):[*] | -0.406 |
| FCFP_2 | 1872154524 | 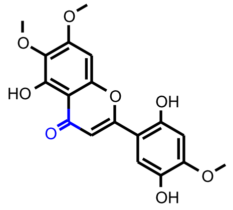 [*]C(=O)[*] | -0.307 |
| FCFP_2 | 0 | 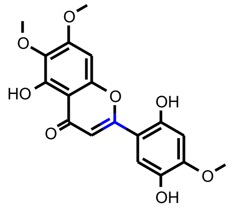 [*]C(=[*])[*] | -0.29 |

# Simeprevir

# TOPKAT_Rat_Maximum_Tolerated_Dose_Gavage

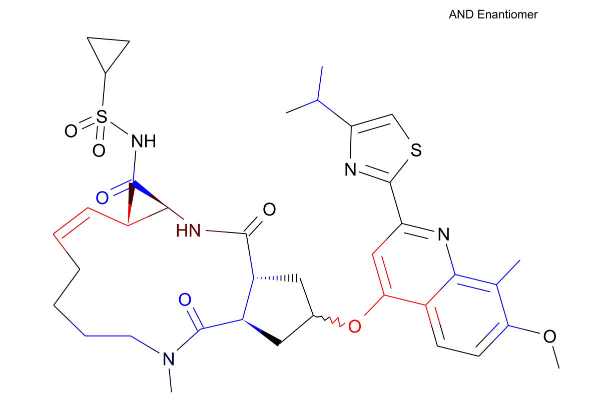


$C_{38}H_{47}N_5O_7S_2$

Molecular Weight: 749.93908

ALogP: 4.796

Rotatable Bonds: 8

Acceptors: 9

Donors: 2

## Model Prediction

Prediction: 0.000856

Unit: g/kg_body_weight

Mahalanobis Distance: 16.8

Mahalanobis Distance p-value: 1.77e-015

Mahalanobis Distance: The Mahalanobis distance (MD) is a generalization of the Euclidean distance that accounts for correlations among the X properties. It is calculated as the distance to the center of the training data. The larger the MD, the less trustworthy the prediction.
Mahalanobis Distance p-value: The p-value gives the fraction of training data with an MD greater than or equal to the one for the given sample, assuming normally distributed data. The smaller the p-value, the less trustworthy the prediciton. For highly non-normal X properties (e.g., fingerprints), the MD p-value is wildly inaccurate.

## Structural Similar Compounds

| Name | OCHRATOXIN | PENICILLIN VK | AMPICILLIN TRIHYDRATE |
|---|---|---|---|
| Structure | 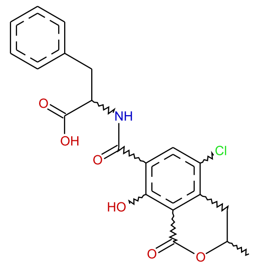 | 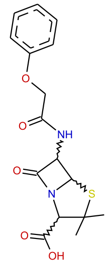 | 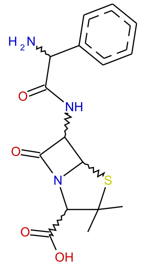 |
| Actual Endpoint (-log C) | 6.28396 | 2.54455 | 2.36724 |
| Predicted Endpoint (-log C) | 5.12358 | 3.9702 | 2.27651 |
| Distance | 1.425 | 1.545 | 1.716 |
| Reference | NCI/NTP TR-358 | NCI/NTP TR-336 | NCI/NTP TR-318 |

## Model Applicability

Unknown features are fingerprint features in the query molecule, but not found or appearing too infrequently in the training set.

1. Molecular_Weight out of range. Value: 749.94. Training min, max, mean, SD: 68.074, 434.63, 171.13, 85.06.
2. Num_H_Acceptors out of range. Value: 9. Training min, max, mean, SD: 0, 6, 1.6146, 1.644.
3. Num_AromaticRings out of range. Value: 3. Training min, max, mean, SD: 0, 2, 0.5625, 0.693.
4. Molecular_PolarSASA out of range. Value: 272.1. Training min, max, mean, SD: 0, 223.97, 50.816, 55.15.
5. Molecular_PolarSurfaceArea out of range. Value: 193.5. Training min, max, mean, SD: 0, 138.03, 28.978, 32.1.
6. OPS PC1 out of range. Value: 9.6287. Training min, max, SD, explained variance: -4.0008, 7.9165, 2.861, 0.2531.
7. OPS PC6 out of range. Value: -3.4118. Training min, max, SD, explained variance: -2.4321, 2.9885, 1.256, 0.0488.
8. Unknown FCFP_2 feature: -415156552: [*]N[C@]1(C[C@H]1[*])C(=[*])[*]
9. Unknown FCFP_2 feature: 690511177: [*]:[cH]:[c](:n:[*])[c](:[*]):[*]
10. Unknown FCFP_2 feature: 690481386: [*]:[c](:[*])[c]1:n:[*]:[*]:s:1
11. Unknown FCFP_2 feature: 1018942292: [*]NS(=O)(=O)C1[*][*]1

## Feature Contribution

**Top features for positive contribution**

| Fingerprint | Bit/Smiles | Feature Structure | Score |
|---|---|---|---|

| | | | |
|---|---|---|---|
| FCFP_2 | 332760439 | 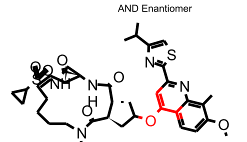  [*]O[c](:[cH]:[*]):[c ]([*]):[*] | 0.672 |
| FCFP_2 | 1 | 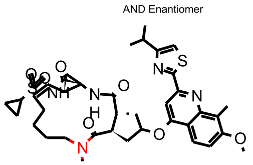  [*]O[*] | 0.511 |
| FCFP_2 | 451847724 | 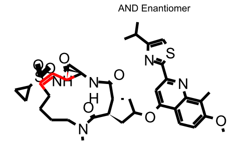  [*]C(=CC(=[*])[*])[*] | 0.225 |

## Top Features for negative contribution

| Fingerprint | Bit/Smiles | Feature Structure | Score |
|---|---|---|---|
| FCFP_2 | 136597326 | 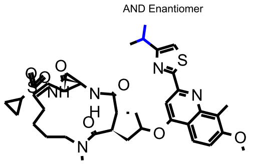  [*]C([*])C | -0.489 |
| FCFP_2 | 203677720 | 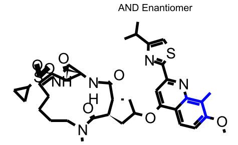  [*]C(=[*])[c](:[c]([* ]):[*]):[c]([*]):[*] | -0.406 |

| | | | |
|---|---|---|---|
| FCFP_2 | 565998553 | AND Enantiomer 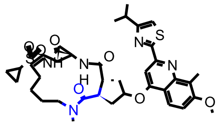 [*]C([*])C(=O)N([*])[<br>*] | -0.348 |

# Flavonoid-1

# TOPKAT_Rat_Oral_LD50

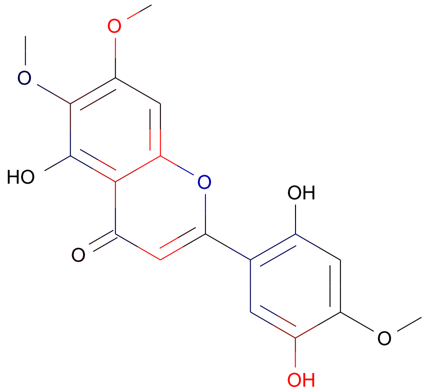


$C_{18}H_{16}O_8$
Molecular Weight: 360.31483
ALogP: 2.361
Rotatable Bonds: 4
Acceptors: 8
Donors: 3

## Model Prediction

Prediction: 0.462
Unit: g/kg_body_weight
Mahalanobis Distance: 17.2
Mahalanobis Distance p-value: 0.0159

Mahalanobis Distance: The Mahalanobis distance (MD) is a generalization of the Euclidean distance that accounts for correlations among the X properties. It is calculated as the distance to the center of the training data. The larger the MD, the less trustworthy the prediction.
Mahalanobis Distance p-value: The p-value gives the fraction of training data with an MD greater than or equal to the one for the given sample, assuming normally distributed data. The smaller the p-value, the less trustworthy the prediciton. For highly non-normal X properties (e.g., fingerprints), the MD p-value is wildly inaccurate.

## Structural Similar Compounds

| Name | ETHYL-bis-COUMACETATE | OCHRATOXIN A | TRIMETHOPRIM |
|---|---|---|---|
| Structure | 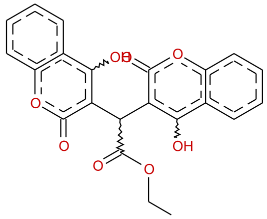 | 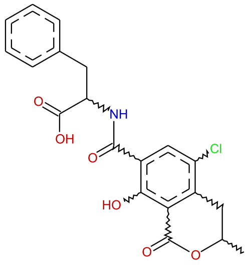 | 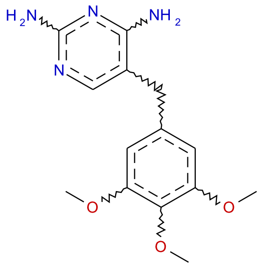 |
| Actual Endpoint (-log C) | 2.687 | 4.305 | 3.162 |
| Predicted Endpoint (-log C) | 2.7054 | 3.03558 | 2.44545 |
| Distance | 0.555 | 0.625 | 0.630 |
| Reference | FEPRA7 10;303;51 | FCTXAV 6;479;68 | 14XBAV -;367;64 |

## Model Applicability

Unknown features are fingerprint features in the query molecule, but not found or appearing too infrequently in the training set.

1. All properties and OPS components are within expected ranges.
2. Unknown FCFP_6 feature: 16: [*][c](:[*]):[*]
3. Unknown FCFP_6 feature: 1618154665: [*][c](:[*]):[cH]:[c]([*]):[*]
4. Unknown FCFP_6 feature: 74595001: [*][c](:[*]):[c](O):[c]([*]):[*]
5. Unknown FCFP_6 feature: -1678245750: [*]O\C(=C/[*])\[c](:[*]):[*]
6. Unknown FCFP_6 feature: -549108873: [*]:[c](:[*])O

## Feature Contribution

| Top features for positive contribution | | | |
|---|---|---|---|
| Fingerprint | Bit/Smiles | Feature Structure | Score |
| ECFP_6 | 642810091 | 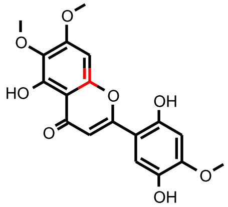 [*][c](:[*]):[*] | 0.281 |

| | | | |
|---|---|---|---|
| ECFP_6 | -560785749 | 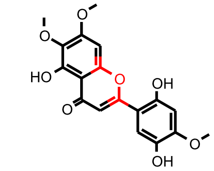 [*]C(=[*])O[c](:[*]):[*] | 0.259 |
| FCFP_6 | 136627117 | 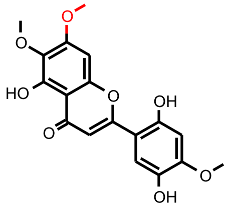 [*]OC | 0.17 |

## Top Features for negative contribution

| Fingerprint | Bit/Smiles | Feature Structure | Score |
|---|---|---|---|
| ECFP_6 | 2106656448 | 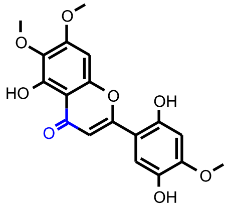 [*]C(=O)[*] | -0.352 |
| ECFP_6 | 683445015 | 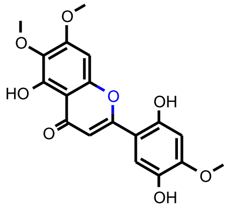 [*]O[*] | -0.266 |
| ECFP_6 | 734603939 | 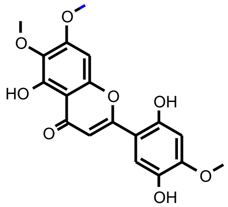 [*]C | -0.201 |

# Simeprevir

# TOPKAT_Rat_Oral_LD50

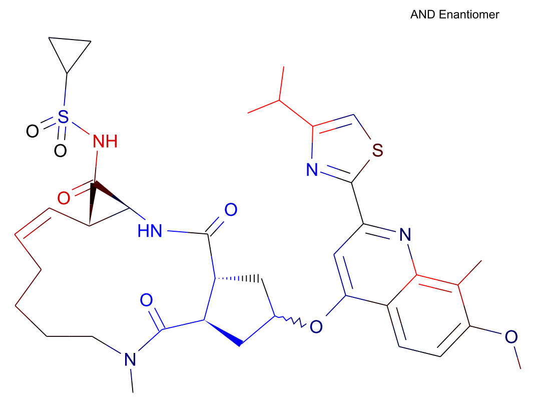


$C_{38}H_{47}N_5O_7S_2$

Molecular Weight: 749.93908

ALogP: 4.796

Rotatable Bonds: 8

Acceptors: 9

Donors: 2

## Structural Similar Compounds

| Name | ANAPREL | RESERPINE | PHOSPHOROTHIOIC ACID; O;O'-(SULFONYLDI-p-PHENYLENE) O;O;O';O'-TETRAMETHYL ESTER |
|---|---|---|---|
| Structure | 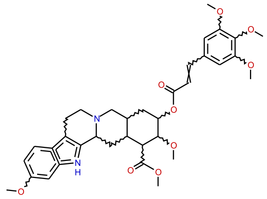 | 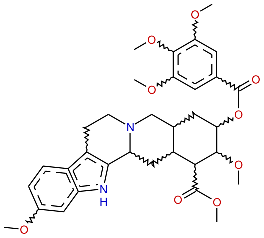 | 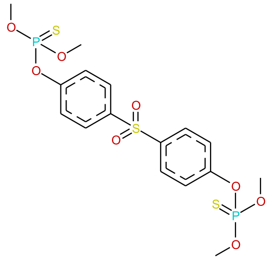 |
| Actual Endpoint (-log C) | 2.803 | 3.161 | 2.397 |
| Predicted Endpoint (-log C) | 2.99154 | 2.72801 | 3.8515 |
| Distance | 1.032 | 1.042 | 1.057 |
| Reference | NIIRDN 6;898;82 | PSSCBG 11;555;80 | TXAPA9 21;315;72 |

## Model Prediction

Prediction: 0.209

Unit: g/kg_body_weight

Mahalanobis Distance: 37.6

Mahalanobis Distance p-value: 1.1e-129

Mahalanobis Distance: The Mahalanobis distance (MD) is a generalization of the Euclidean distance that accounts for correlations among the X properties. It is calculated as the distance to the center of the training data. The larger the MD, the less trustworthy the prediction.

Mahalanobis Distance p-value: The p-value gives the fraction of training data with an MD greater than or equal to the one for the given sample, assuming normally distributed data. The smaller the p-value, the less trustworthy the prediciton. For highly non-normal X properties (e.g., fingerprints), the MD p-value is wildly inaccurate.

## Model Applicability

Unknown features are fingerprint features in the query molecule, but not found or appearing too infrequently in the training set.

1. All properties and OPS components are within expected ranges.
2. Unknown ECFP_2 feature: -309676563: [*]N[C@]1(C[C@H]1[*])C(=[*])[*]
3. Unknown ECFP_2 feature: -1049290660: [*]C1([*])C[C@H]1C=[*]
4. Unknown ECFP_2 feature: 2121941848: [*]NS(=O)(=O)C1[*][*]1
5. Unknown FCFP_6 feature: 16: [*][c](:[*]):[*]
6. Unknown FCFP_6 feature: 1747237384: [*][c](:[*]):n:[c](:[*]):[*]
7. Unknown FCFP_6 feature: 690511177: [*]:[cH]:[c](:n:[*])[c](:[*]):[*]
8. Unknown FCFP_6 feature: 1618154665: [*][c](:[*]):[cH]:[c]([*]):[*]
9. Unknown FCFP_6 feature: 690481386: [*]:[c](:[*])[c]1:n:[*]:[*]:s:1
10. Unknown FCFP_6 feature: 1018942292: [*]NS(=O)(=O)C1[*][*]1

## Feature Contribution

### Top features for positive contribution

| Fingerprint | Bit/Smiles | Feature Structure | Score |
|---|---|---|---|

| | | | |
|---|---|---|---|
| FCFP_6 | 1186303932 | 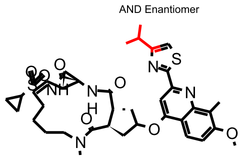 [*]:[c](:[*])C(C)C | 0.375 |
| ECFP_6 | 642810091 | 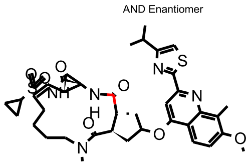 [*][c](:[*]):[*] | 0.281 |
| ECFP_6 | -1897341097 | 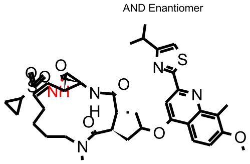 [*]N[*] | 0.216 |

## Top Features for negative contribution

| Fingerprint | Bit/Smiles | Feature Structure | Score |
|---|---|---|---|
| ECFP_6 | 2106656448 | 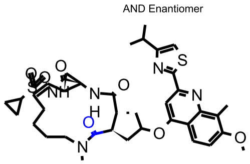 [*]C(=O)[*] | -0.352 |
| ECFP_6 | 2077607946 | 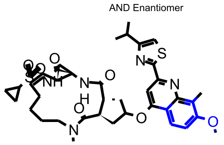 [*]O[c]1:[cH]:[cH]:[c ](:[*]):[*]:[c]:1[*] | -0.252 |

| | | | |
|---|---|---|---|
| ECFP_6 | 655739385 | 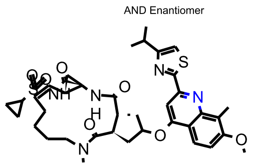 [*]:n:[*] | -0.239 |
